# Solar Gas-Phase CO_2_ Hydrogenation by Multifunctional UiO-66 Photocatalysts

**DOI:** 10.1021/acscatal.4c00266

**Published:** 2024-04-12

**Authors:** Celia M. Rueda-Navarro, Zahraa Abou Khalil, Arianna Melillo, Belén Ferrer, Raúl Montero, Asier Longarte, Marco Daturi, Ignacio Vayá, Mohamad El-Roz, Virginia Martínez-Martínez, Herme G. Baldoví, Sergio Navalón

**Affiliations:** † Departamento de Química, Universitat Politècnica de València, Camino de Vera s/n ,Valencia 46022, Spain; ‡ ENSICAEN, UNICAEN, CNRS, Laboratoire Catalyse et Spectrochimie, Normandie Université, Caen 14000, France; § SGIker Laser Facility, UPV/EHU, Sarriena, s/n ,Leioa 48940, Spain; ∥ Facultad de Ciencia y Tecnología, Departamento de Química Física, Universidad del País Vasco (UPV/EHU), Apart. 644 ,Bilbao 48080, Spain; ⊥ Instituto de Tecnología Química (UPV-CSIC), Universitat Politècnica de València, C/Avenida de los Naranjos s/n ,Valencia 46022, Spain

**Keywords:** heterogeneous photocatalysis, multifunctional metal−organic frameworks: UiO-66 topology, CO_2_ methanation, solar light

## Abstract

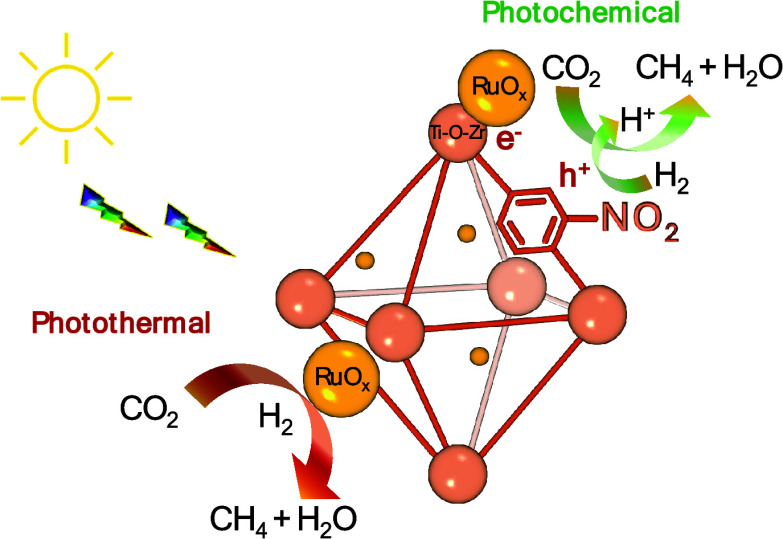

Solar-assisted CO_2_ conversion into fuels and chemical products involves a range of technologies aimed at driving industrial decarbonization methods. In this work, we report on the development of a series of multifunctional metal–organic frameworks (MOFs) based on nitro- or amino-functionalized UiO-66(M) (M: Zr or Zr/Ti) supported RuO_*x*_ NPs as photocatalysts, having different energy band level diagrams, for CO_2_ hydrogenation under simulated concentrated sunlight irradiation. RuO_*x*_(1 wt %; 2.2 ± 0.9 nm)@UiO-66(Zr/Ti)-NO_2_ was found to be a reusable photocatalyst, to be selective for CO_2_ methanation (5.03 mmol g^–1^ after 22 h;, apparent quantum yield at 350, 400, and 600 nm of 1.67, 0.25, and 0.01%, respectively), and to show about 3–6 times activity compared with previous investigations. The photocatalysts were characterized by advanced spectroscopic techniques like femto- and nanosecond transient absorption, spin electron resonance, and photoluminescence spectroscopies together with (photo)electrochemical measurements. The photocatalytic CO_2_ methanation mechanism was assessed by operando FTIR spectroscopy. The results indicate that the most active photocatalyst operates under a dual photochemical and photothermal mechanism. This investigation shows the potential of multifunctional MOFs as photocatalysts for solar-driven CO_2_ recycling.

## 1 Introduction

The present level of burning fossil fuels to meet the world’s energy requirements is steadily raising the CO_2_ emissions released into the atmosphere and is responsible for global warming and climate change.^1,2^ There is thus an urgent need to shift from these fuels to renewable energy obtained from natural resources like the sun, wind, water, or biomass.^3,4^ The development of technologies based on carbon-free energy carriers like green hydrogen is considered vital to help decarbonize the world’s economies,^5,6^ whereas carbon capture, storage, and utilization (CCSU) are some processes that can minimize the negative effects of CO_2_ emissions.^7,8^ Even though certain CCS processes have achieved relative success, most of the technologies used to convert CO_2_ into valuable products or fuels are still under development,^7−13^ including solar-assisted photocatalysis, which is considered to be a promising cost-efficient and sustainable process for recycling CO_2_.^14−19^ In 1978, a pioneering study reported on the possibility of reducing CO_2_ using GaP as the photoelectrocatalyst.^20^ Since then, many other inorganic semiconductors^18,21−24^ and, more recently, perovskites,^23,25^ carbon-based materials similar to graphenes,^23,26,27^ or carbon nitrides,^23,28^ among others,^23,29^ have been used for this purpose. H_2_ as the reducing agent seems to be more suitable for achieving better performance than H_2_O.^30^ Because it is expected that green hydrogen will be economically feasible in the medium and long term, this innovation will boost the large-scale production of compounds and fuels from CO_2_ hydrogenation.^31^ Of these, the photocatalytic solar-driven reduction of CO_2_ by H_2_ to CH_4_, a process also termed as the *photocatalytic Sabatier reaction*, is attracting increasing interest for the transition to zero net emissions.^32−34^ This process considerably improves the efficiency of the thermocatalytic reaction even when working under mild reaction conditions.^32^ For example, photocatalytic CO_2_ methanation can be carried out at much lower reaction temperatures (∼200 °C)^25^ than the thermocatalytic version (300–350 °C) while achieving similar results.^25,32^ The synthetic methane thus obtained can then be directed to the existing natural gas infrastructures to minimize its implementation costs.^33^ To a lesser extent, other related studies have also shown the possibility of performing the photocatalytic CO_2_^30^ or CO^35^ hydrogenation into C_2+_ and even C_5+_ value-added chemicals and fuels.

A relatively new emerging research field for solar-driven photocatalytic Sabatier reaction using metal–organic frameworks (MOFs).^36^ is under development. MOFs are porous crystalline materials built from multitopic organic ligands coordinated to metal ions, metal clusters, or metal-oxo chains.^37−39^ For about 20 years, MOFs have been considered as highly tunable photocatalysts for many organic and inorganic reactions.^31,40−42^ In the field of CO_2_ photoreduction, most of the knowledge achieved so far has come from the liquid-phase reaction using organic solvents in the presence of sacrificial electron donors under UV–vis or visible light irradiation.^40^ Acetonitrile is frequently used as a solvent to favor CO_2_ dissolution, whereas triethanolamine is employed as the electron donor to recover photogenerated holes, minimize electron–hole recombination, and thus increase the efficiency of the reduction process.^40^ These studies on MOFs represent an interesting area of research in understanding the theoretical and practical aspects of CO_2_ conversion.

A series of recent studies have reported on using MOFs as photocatalysts for gas-phase CO_2_ reduction by H_2_ under interesting reaction conditions for large-scale processes. The possibility of using MOF-based materials for the photocatalytic gas-phase Sabatier reaction under UV–vis at 215 °C^36^ was reported for the first time in 2019. Since then, other studies have described a process with MOF-based photocatalysts modified with RuO_*x*_ NPs for solar-assisted CO_2_ methanation at 200 °C. RuO_*x*_ NPs are the benchmark cocatalyst in achieving high efficiency during CO_2_ (photo)methanation.^32^ Some of these photocatalysts include Ti-MOFs, such as MIP-208(Ti)^43^ or MIL-125(Ti)-NH_2_^44^ functionalized with NH_2_ groups. The presence of amino groups determines the MOF energy band level, i.e., a band gap reduction and a negative shift of the lowest unoccupied crystal orbital (LUCO) with respect to the nonfunctionalized parent MOF, and favors the thermodynamics of the reduction processes.^45,46^ Other studies have reported that amino groups in MOFs favor the stabilization of photogenerated holes and, in turn, the photoinduced charge separation efficiency.^47,48^ Amino-MOFs like UiO-66(Zr)-NH_2_ have a higher CO_2_ adsorption capacity than the analogous UiO-66(Zr)-NO_2_ due to the bonding capacity of the amino groups.^49^ Despite the research on the possibility of tuning the energy band diagram of MOFs with functional groups other than amino groups, such as nitro, bromo, or methyl groups, and their resulting photocatalytic activity, few studies have to date addressed its influence on photocatalytic CO_2_ hydrogenation.^45,50,51^ Other related studies have shown that mixed-metal MOFs involve higher photocatalytic activity in CO_2_ reduction.^45,52^ For example, the better performance of the UiO-66(Zr/Ti)-based photocatalyst than UiO-66(Zr) is associated with the role of Ti(IV) as the electron mediator that favors photoinduced ligand-to-metal charge transfer (LMCT) processes from the organic ligand to the metal node.^52,53^ Despite these important findings, as far as we know, no studies have yet explored the possibility of developing multifunctional MOF-based materials with a unique energy band diagram determined by the presence of specific functional groups, e.g., the amino or nitro groups, simultaneously containing mixed-metal nodes for more effective photoinduced charge separation and cocatalysts to boost the solar-assisted photocatalytic Sabatier reaction.

In this context, we report here the development of multifunctional nitro- or amino functionalized Zr(IV)- or Zr(IV)/Ti(IV)-based-MOFs with a UiO-66 topology-supported RuO_*x*_ NPs for the solar-driven solid–gas phase Sabatier reaction. The materials were characterized by powder X-ray diffraction (PXRD), analytical, spectroscopic, and electron microscopy techniques, and their photocatalytic activities were tested under simulated concentrated sunlight irradiation. Femto- and nanosecond transient absorption (TAS), photoluminescence (PL), electron spin resonance (ESR), and electrochemical impedance (EIS) spectroscopies together with transient photocurrent measurements and additional specific photocatalytic experiments were used to determine the role of MOF counterparts during CO_2_ photomethanation via a likely dual photochemical and photochemical mechanism. The photocatalytic CO_2_ hydrogenation pathway was studied by operando FTIR spectroscopy.

## 2 Experimental Section

Details of the materials, preparation, characterization, and photocatalytic procedures used in the study can be found in the Supporting Information (Sections S1–S3).

### 2.1 Materials, Preparation Methods, and Characterization

All the materials employed in this study were of analytical or HPLC grade and supplied by Merck. UiO-66(Zr)-NH_2_ and UiO-66(Zr)-NO_2_ were prepared according to previous procedures^54−56^ and were postsynthetically modified by a titanium(IV) chloride tetrahydrofuran complex [TiCl_4_(THF)_2_] complex to obtain UiO-66(Zr/Ti)-NH_2_ and UiO-66(Zr/Ti)-NO_2_ as reported.^57,58^ RuO_*x*_ NPs were supported on these four UiO-66 solids using the photodeposition method.^44^

The solids were characterized by PXRD, UV–vis diffuse reflectance (UV–vis DRS), X-ray photoelectron (XPS), electron spin resonance (ESR), steady-state PL, EIS, femto- and nanosecond TAS spectroscopies, and electron microscopy, including transmission electron microscopy (TEM) or scanning transmission electron microscopy (SEM) coupled with an energy-dispersive X-ray electron (EDX) detector. Isothermal N_2_ adsorption, thermogravimetric, and photoelectrochemical measurements were also used.

### 2.3 Photocatalytic Activity

Photocatalytic reactions were carried out under batch reaction conditions (Section S3), and the data given here are the average of at least three separate experiments.

## 3 Results and Discussion

### 3.1 Photocatalyst Characterization

**Figure 1:**
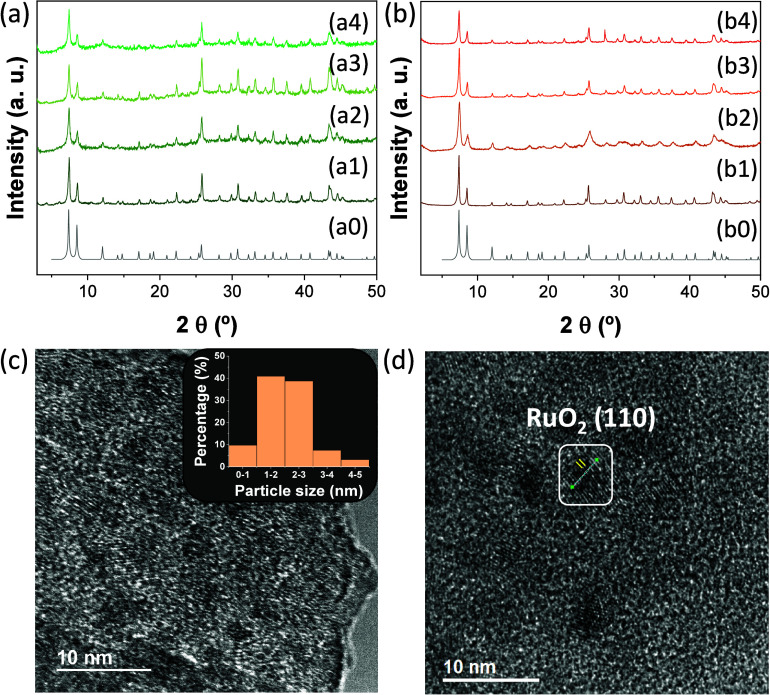
XRD of simulated UiO-66 (a0, b0) or PXRD of UiO-66(Zr)-NH_2_ (a) or UiO-66(Zr)-NO_2_ (b) materials. Legend panel a: UiO-66(Zr)-NH_2_ (a1), RuO_*x*_@UiO-66(Zr)-NH_2_ (a2), UiO-66(Zr/Ti)-NH_2_ (a3), and RuO_*x*_@UiO-66(Zr/Ti)-NH_2_ (a4). Legend panel b: UiO-66(Zr)-NO_2_ (b1), RuO_*x*_@UiO-66(Zr)-NO_2_ (b2), UiO-66(Zr/Ti)-NO_2_ (b3), and RuO_*x*_@UiO-66(Zr/Ti)-NO_2_ (b4). (c) HRTEM image and RuO_*x*_ particle size distribution of RuO_*x*_@UiO-66(Zr/Ti)-NO_2_; RuO_*x*_ average particle size and standard deviation of 2.08 ± 0.82 nm. (d) *d*-spacing is determined (0.32 nm) from the HRTEM image of RuO_*x*_@UiO-66(Zr/Ti)-NO_2_.

The MOF-based materials prepared, i.e., UiO-66(M)-X (M: Zr and/or Ti; X: NH_2_ or NO_2_), both loaded or unloaded with RuO_*x*_ NPs, were characterized by different techniques. PXRD analyses revealed that these solids had the expected UiO-66 topology (Figure 1).^56^ The ICP-OES analyses of acid-digested MOFs were used to quantify the zirconium and/or titanium elements, either loaded or not loaded with RuO_*x*_ NPs at 1 wt % of ruthenium. UiO-66(Zr/Ti)-NH_2_ and UiO-66(Zr/Ti)-NO_2_ have a titanium content of 0.9 and 1.3 wt %, respectively. In this regard, previous studies reported that postsynthetic modification (PSM) of UiO-66(Zr) based materials with TiCl_4_(THF)_2_ complex results in the incorporation of Ti(IV) in the solid by metal exchange and/or grafting onto the metal node at the linker vacancy.^59^ Partial replacement of Zr(IV) by Ti(IV) ions with smaller ionic radii contracts the unit cell reflected in PXRD by a small negative shift of the position of the diffraction peaks. In the present work, UiO-66(Zr/Ti)-X (X: NH_2_ or NO_2_) solids showed similar PXRD peak positions to those in zirconium, indicating that Ti(IV) ions are mostly grafted onto the MOF metal nodes.^57,59^ The PXRD of UiO-66 solids loaded with RuO_*x*_ NPs have similar features to those of the parent MOFs. The absence of RuO_*x*_ diffraction peaks was attributed to the low ruthenium loading (1 wt %) in the MOF and/or good dispersion of small NPs.^44^

The HR-SEM analyses showed that UiO-66 crystals are characterized by the agglomeration of small cubes with average particle sizes and standard deviations of 118 ± 57 nm (Figure S1). HR-SEM in combination with EDX analyses (Figures S2–S10) showed a good distribution of MOF elements within the particles. The relatively low intensity of ruthenium due to its low loading (1 wt % Ru) was within the instrument’s detection limit. DF-STEM coupled with EDX and HR-TEM measurements characterized RuO_*x*_ NPs (2.14 ± 0.86 nm) supported on UiO-66 particles. HRTEM measurements (Figures S11–S14) indicated the presence of 0.32 nm lattice spacings (Figures S15–S17), characteristic of the (110) facet of RuO_2_.^60^

**Figure 2:**
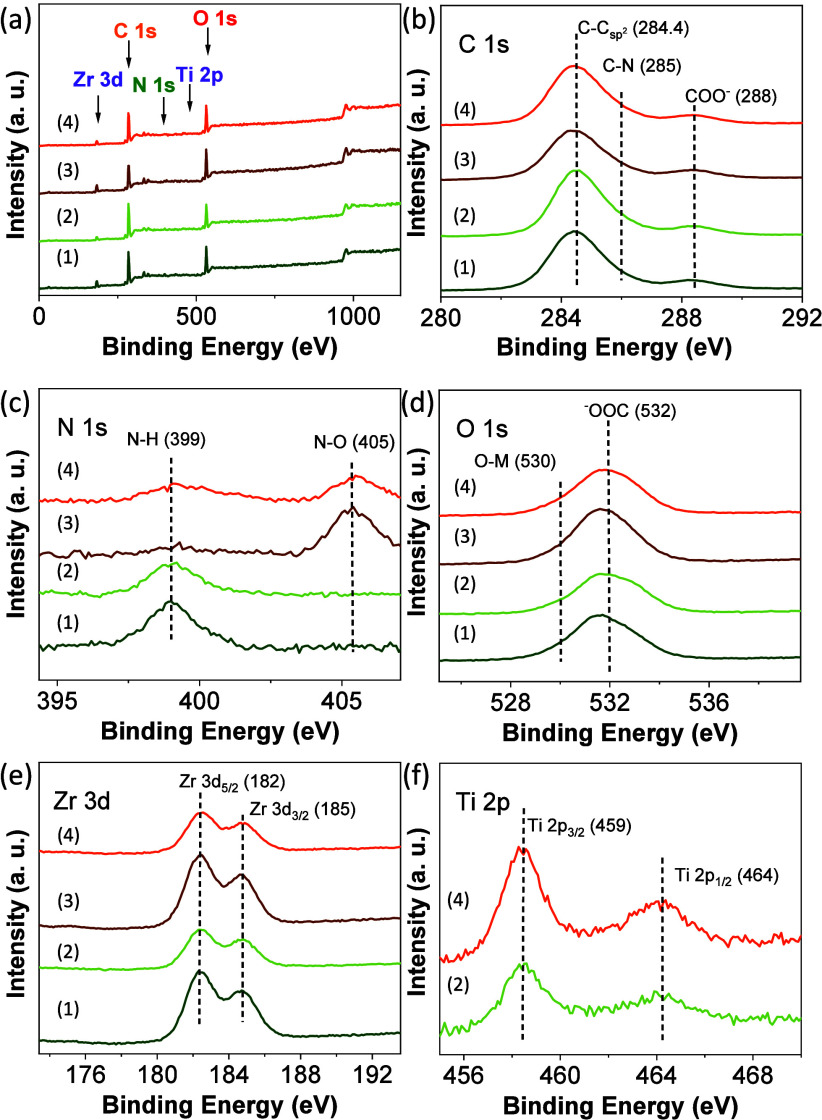
XPS survey (a), C 1s (b), O 1s (c), N 1s (d), Zr 3d (e), and Ti 3p (f) of UiO-66(Zr)-NH_2_ (1), UiO-66(Zr/Ti)-NH_2_ (2), UiO-66(Zr)-NO_2_ (3), and UiO-66(Zr/Ti)-NO_2_ (4).

The UiO-66 samples were also characterized by XPS (Figure 2 and Figures S18–S21) to determine the oxidation state of the elements within the solids. The XPS spectra of the C 1s region are associated with the presence of the 2-amino or 2-nitroterephthalates ligands of the MOFs: C–C sp^2^ bonds (284.4 eV), COO^–^ groups (288 eV), and C–N bonds of amino or nitro (∼285 eV) groups. The N 1s XPS of amino-functionalized UiO-66 solids shows the expected C–N signal at about 399 eV. In the case of nitro-functionalized UiO-66 materials, N 1s XPS spectra are dominated by a main band at 405 eV due to the nitro group, whereas a signal associated with the presence of an amino group can also be detected. This situation, i.e., the presence of a small band assigned to the amino group when preparing nitro-functionalized UiO-66 solids, has previously been reported.^51^ For the series of RuO_*x*_ NPs supported UiO-66 solids, the XPS Ru 3d spectra showed a weak band centered at about 282 eV (Figures S22 and S25), partially overlapping with C–C sp^2^ bond signals (284.4 eV), which can be assigned to the presence of RuO_2_ NPs.^44^ Supported RuO_2_ NPs were further characterized by Ru 3p XPS, where the expected two bands could be seen at about 462.5 and 485 eV characteristic of Ru 3p_3/2_ and Ru 3 p_1/2_, respectively. The O 1s XPS signal was assigned to the presence of COO^–^ groups (532 eV) and M–O bonds (M: Zr, Ti or Ru) (530 eV). Zr 3d and Ti 2p XPS spectra showed the expected signals of Zr(IV) and Ti(IV) ions in the UiO-66 structure. Zr 3d XPS spectra had two bands centered at about 182 and 185 eV due to Zr 3d_5/2_ and Zr 3d_3/2_, respectively. The XPS spectra of the Ti 2p region for mixed-metal UiO-66(Zr/Ti)-X (X: NH_2_ or NO_2_) confirmed the presence of Ti(IV) indicated by two bands at 459 and 464 eV due to Ti 2p_3/2_ and Ti 2p_1/2_, respectively.

The UiO-66 solids were analyzed by FTIR spectroscopy (Figure S26). In all cases, COO^–^ groups were characterized by stretching vibrations at about 1574 and 1423 cm^–1^, respectively. Amino-functionalized UiO-66 solids showed two bands at 3488 and 3374 cm^–1^ due to the asymmetric and symmetric vibrations of −NH_2_, respectively, together with another band at 1255 cm^–1^ due to C–N stretching vibration. In the case of nitro-functionalized UiO-66 solids, two bands could be seen at about 1543 and 1496 cm^–1^ due to the characteristic asymmetric and symmetric vibration bands of this group, respectively. These spectra also showed small bands attributable to the presence of amino groups, in good agreement with the XPS analyses. These XPS and FTIR results indicate a need for the development of new synthetic methodologies to prepare UiO-66 solids with only 2-nitroterephthalte ligands in their structure.

Isothermal N_2_ adsorption measurements were used to estimate the BET surface areas (Figure S27) and pore volumes of pristine mono- and bimetallic UiO-66 solids with values ranging from 600 to 700 m^2^/g and 0.23 to 0.26 cm^3^/g, respectively, in agreement with previous studies.^57^ TGA analyses under oxidant (air) or inert (nitrogen) atmospheres further confirmed that these UiO-66 samples are thermally stable at temperatures of about 300 °C, and these observations are in agreement with previous reports (Figure S28).^57,61,62^ It should be commented that the stability observed below 300 °C under these atmospheres might differ somehow the stability under the reaction conditions of photocatalytic CO_2_ hydrogenation (H_2_/CO_2_ molar ratio 4:1 at 200 °C). Additionally, a control experiment revealed that the TGA of UiO-66(Zr/Ti)-NO_2_ solid previously submitted to these reaction conditions exhibited a very similar TGA profile under air than the fresh sample, thus confirming its relative stability under studied reaction conditions.

**Figure 3:**
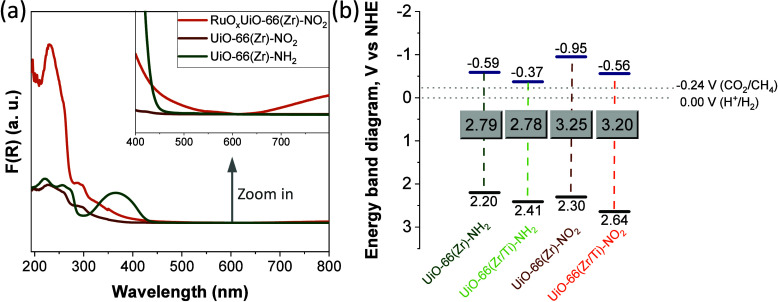
(a) UV–vis DRS and (b) energy band level diagram of UiO-66 solids as indicated.

The optical properties of the UiO-66 materials were studied by UV–vis DRS measurements. Figure 3 shows that the presence of NO_2_ and especially NH_2_ groups in the MOF organic ligand favors visible light absorption with absorption onsets at about 400 and 450 nm, respectively. In the case of amino-functionalized UiO-66 solids, the band centered at about 365 nm is due to the interaction of the lone pair of electrons of amino group with the π*-orbital of aromatic ring, and this situation results in a new higher HOCO level that favors visible light absorption.^63^ Tauc plot analyses using the UV–vis DRS data (Figure S29) confirmed that the optical band gaps of amino-functionalized UiO-66 solids were lower than those of the nitro-functionalized UiO-66 solids.^64^ Besides, mixed-metal UiO-66 solids exhibit somehow lower optical band gaps associated with the role of Ti(IV) ions as electron mediators in agreement with previous experimental^48,58^ and theoretical studies.^65^ XPS valence band measurements (Figure S30) were used to estimate the UiO-66 energy band diagrams together with the optical band gaps. In general, all the solids possessed the thermodynamic requirements for photocatalytic CO_2_ hydrogenation under sunlight irradiation, whereas the UV–vis DRS of RuO_*x*_ NPs on UiO-66 solids showed an extra weak absorption band in the visible region associated with the resonance plasmon band of these NPs (Figure S31).

### 3.2 Photocatalytic CO_2_ Hydrogenation

**Figure 4:**
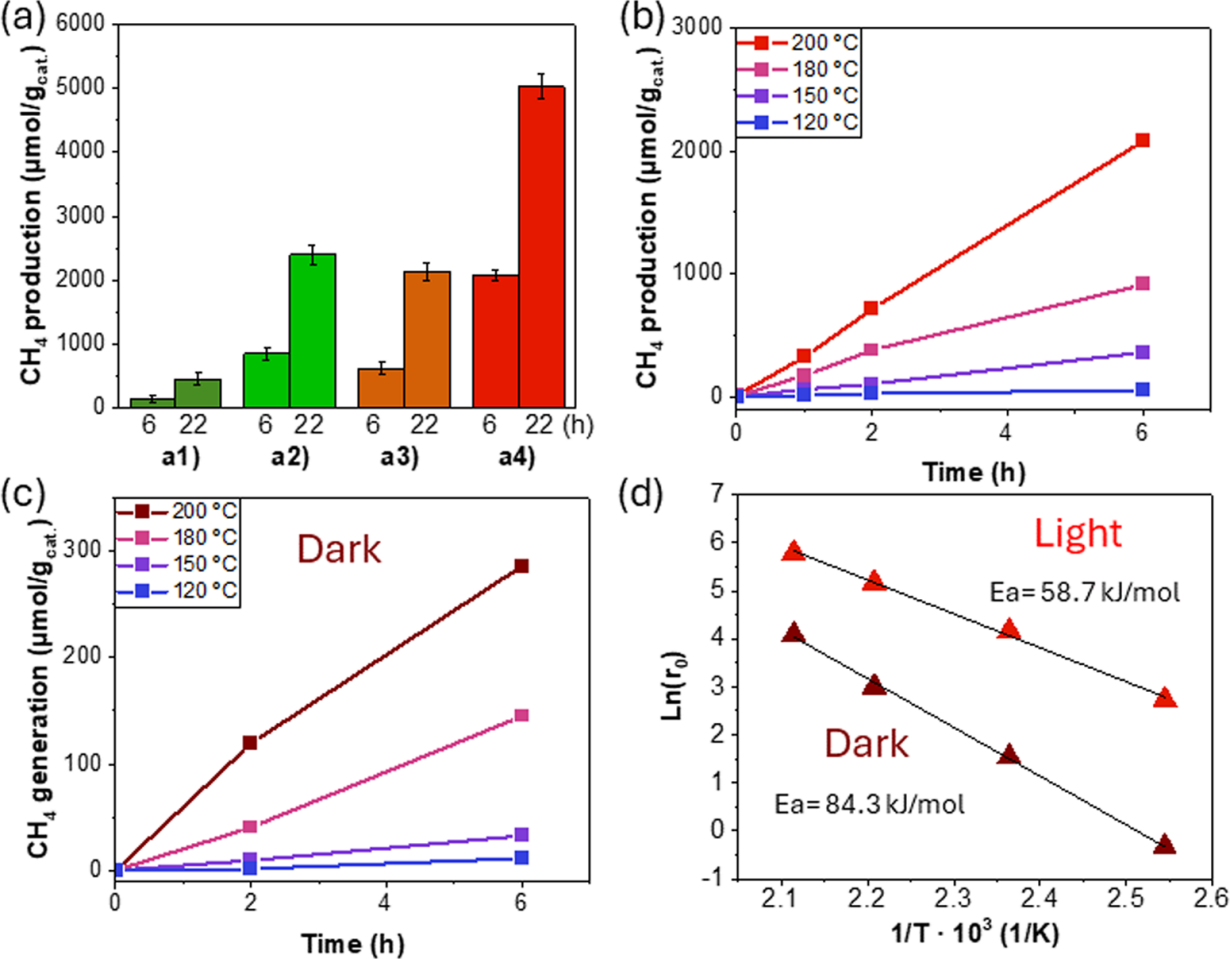
(a) Photocatalytic CO_2_ methanation using RuO_*x*_@UiO-66 solids under simulated concentrated sunlight irradiation. Legend: (a1) UiO-66(Zr)-NH_2_, (a2) UiO-66(Zr/Ti)-NH_2_, (a3) UiO-66(Zr)-NO_2_, and (a4) UiO-66(Zr/Ti)-NO_2_. Influence of reaction temperature on methane generation during photocatalytic CO_2_ reduction under light (b) or dark (c) conditions. (d) The Arrhenius plot obtained from initial reaction rates of methane generation as a function of the reaction temperature under dark or light conditions as indicated. Reaction conditions: photocatalyst (15 mg), CO_2_/H_2_ (1:4), 200 °C, simulated concentrated sunlight (200 mW/cm^2^) irradiation.

UiO-66-based solids were first tested as photocatalysts for CO_2_ hydrogenation at 200 °C under simulated concentrated sunlight irradiation (200 mW/cm^2^). For this purpose, the quartz reactor is heated with a mantle, and then the system was irradiated (see details in Section 2). It should be remembered that 1 sun is defined as 100 mW/cm^2^ of irradiance. From the point of view of practical applications, solar concentrators could be used to reach the simulated concentrated sunlight irradiations used in this study. Pristine UiO-66 solids showed little activity, and methane was the only product detected (<30 μmol g^–1^). Specifically, to illustrate the importance of supported RuO_*x*_ NPs in enhancing the photocatalytic activity, the performance of UiO-66(Zr)-NH_2_, UiO-66(Zr/Ti)-NH_2_, UiO-66(Zr)-NO_2_, and UiO-66(Zr/Ti)-NO_2_ was evaluated and observing only 2, 13, 3, and 4 μmol·g^–1^ after 22 h, respectively. However, RuO_*x*_ NPs supported UiO-66 materials boosted activities toward methane generation by various degrees, in agreement with the role of RuO_*x*_ NPs as benchmark cocatalysts for selective CO_2_ (photo)catalytic methanation.^32^ RuO_*x*_ NPs have the ability to favor chemisorption CO_2_ and its reaction intermediates like CO or H_2_CO with sufficient strength to be completely hydrogenated to methane.^34^ Even though our analyses allow identification and quantification of several carbon products such as CO or short-chain hydrocarbons (see Supporting Information Section S3), methane was the main product together with small amounts of ethane detected for all tested photocatalysts. In other words, all (photo)catalytic tests carried out in this study resulted in methane selectivities higher than 99%. Control experiments in which CO_2_ was replaced by Ar did not indicate the formation of methane or any other product. Because of the similar particle size distribution of RuO_*x*_ NPs supported on UiO-66 solids, i.e., a mean average particle size and standard deviations of 2.14 ± 0.04 nm, we consider that the composition of the UiO-66 photocatalysts determines the resulting activities. Furthermore, it was found that product selectivity is not influenced by the use of UiO-66 composition loaded or not with RuO_*x*_ NPs. As an example, the product selectivity distribution of the most active RuO_*x*_@UiO-66(Zr/Ti)-NO_2_ indicates a CH_4_ selectivity higher than 99% accompanied by ethane. Figure 4 shows that nitro-functionalized UiO-66 photocatalysts are more active than amino-functionalized UiO-66 photocatalysts. This is an important finding because, as commented in the introduction, amino-functionalized MOFs like UiO-66 are among the preferred solids for photocatalytic applications, including CO_2_ reduction. Regardless of UiO-66(Zr)-NO_2_’s higher optical band gap than UiO-66(Zr)-NH_2_ (3.16 vs 2.79 eV), its better reduction and oxidation capacity than those of the amino group seems to determine its photocatalytic activity (see Figure 3). Figure 4 also shows that the photocatalytic activities of RuO_*x*_ NPs supported UiO-66(Zr)-X (X: NH_2_ or NO_2_) are further increased by the preparation of analogous mixed-metal Zr/Ti materials. Previous studies have demonstrated the role of Ti(IV) ions in the metal node of UiO-66(Zr/Ti)-NH_2_ as photoinduced electron transfer mediators.^48,58^ As will be shown below, the better performance of mixed-metal UiO-66 photocatalysts supported by RuO_*x*_ NPs than those analogous monometallic ones can be attributed to the increased photoinduced charge separation efficiency, as shown by the spectroscopic and electrochemical characterization.

To further verify the role of nitro or amino groups in UiO-66(Zr)-X (X: NO_2_ or NH_2_) on the resulting photocatalytic activity, an analogous photocatalyst termed as UiO-66(Zr) was prepared using terephthalic acid as organic ligand and further modified with RuO_*x*_ NPs by the photodeposition method. The samples were characterized by PXRD, spectroscopic (UV–vis, XPS), analytical (TGA), textural (isothermal N_2_ adsorption), and electron microscopic techniques (Figures S32–S37). PXRD confirmed that RuO_*x*_@UiO-66(Zr) and UiO-66(Zr) samples are isostructural crystalline materials with UiO-66 topology (Figure S32). XPS analyses revealed the general expected features of XPS C 1s, O 1s, Zr Ru 3d, and 3p (Figure S33). These solids are constituted by particles of 98 ± 63 nm as revealed by SEM analyses (Figure S34). TEM measurements revealed the presence of supported RuO_*x*_ NPs with sizes of 2.4 ± 0.8 nm (Figure S35). The sample exhibited good porosity (1008 m^2^/g and 0.38 cm^3^/g) and thermal stability under air atmosphere (>400 °C) (Figure S36). The energy band level diagram of UiO-66(Zr) is characterized by a wide optical band gap (3.7 eV) with HOCO and LUCO positions of +1.81 and −2.15 V, respectively (Figure S37). The use of RuO_*x*_@UiO-66(Zr) and pristine UiO-66(Zr) as photocatalysts under conditions described in Figure 4 showed a selective methane production of 500 and 2 μmol g^–1^, respectively, after 22 h. The activity of this RuO_*x*_@UiO-66(Zr) photocatalyst is slightly lower than that of RuOx@UiO-66(Zr)-NH_2_ and about three times lower than that achieved using the RuO_*x*_@UiO-66(Zr)-NO_2_ photocatalyst. Regardless of the lower CO_2_ adsorption capacity and higher optical band gap of UiO-66(Zr) compared to UiO-66(Zr)-NH_2_, their photocatalytic activities are similar to each other. In contrast, as previously commented, RuO_*x*_@UiO-66(Zr)-NO_2_ exhibits higher activity associated with its unique structure due to the presence of nitro functional groups. The performance of the most active RuO_*x*_@UiO-66(Zr/Ti)-NO_2_ sample (∼13% CO_2_ conversion; 5.03 mmol_CH4_·g^–1^ after 22 h) during photocatalytic CO_2_ hydrogenation to CH_4_ was further studied. A photocatalytic experiment using labeled ^13^CO_2_ and gas-phase aliquot analysis by GC coupled to mass spectrometer using an electron ionization method confirmed the formation of ^13^CH_4_ (*m*/*z* 17) after 22 h of reaction at 200 °C (Figure S38). It should be noted, however, that the characteristic ionization profile of methane differs to some extent to the one obtained and associated with the contribution of other molecules like H_2_O and air from ambient during the injection that are not chromatographically separated in our system. As will be shown later in Section 3.3.2, the transformation of CO_2_ into CH_4_ has been further confirmed by using operando FTIR analyses. A control experiment under dark reaction conditions at 200 °C also revealed lower CH_4_ production (1.9 mmol g^–1^ after 22 h) than that achieved under simulated concentrated sunlight irradiation. The observation of some activity under dark reaction conditions was not unexpected because previous studies have reported that RuO_*x*_ NPs are an active and selective cocatalyst during thermal catalytic processes.^32^ Quantitative information on the performance of RuO_*x*_@UiO-66(Zr/Ti)-NO_2_ as a photocatalyst at 200 °C was obtained by estimating the apparent quantum yield (AQY) at specific wavelengths. After deducting the activity observed under dark reaction conditions, the AQYs achieved by irradiation at 350, 400, and 600 nm were 1.67, 0.25, and 0.01%, respectively. The influence of the reaction temperature on the photocatalytic activity of RuO_*x*_@UiO-66(Zr/Ti)-NO_2_ was then studied (see results in Figure 4b). As can be seen, photocatalytic methane generation as a function of the reaction temperature follows the Arrhenius law and allowed us to estimate an apparent activation energy (Ea) of 58.7 kJ/mol. In a series of analogous experiments carried out in the absence of irradiation (thermal catalysis), the estimated Ea resulted to be 84.3 kJ/mol. Based on analogous studies^66−68^ and as will be further studied in Section 3.3, this significant decrease in Ea can be attributed to the operation of a photothermal reaction pathway.

The photocatalytic activity of RuO_*x*_@UiO-66(Zr/Ti)-NO_2_ was compared with those MOF-based photocatalysts reported in previous studies, and the results are summarized in Table S1. The use of the same reaction conditions than most of the studies in Table S1, i.e., *P*_H2_ = 1.05 bar and *P*_CO2_ = 0.25 bar instead the previous *P*_H2_ = 1.2 bar and *P*_CO2_ = 0.3 bar, resulted in a methane production decrease of about 5% in agreement with Chatelier’s principle. RuO_*x*_ NPs supported trimetallic UiO-66(Zr/Ce/Ti) was recently reported as one of the most active MOF-based photocatalysts for CO_2_ methanation under simulated concentrated sunlight irradiation (1.8 mmol g^–1^ CH_4_ after 22 h at 200 °C) (Table S1, entry 2), showing that the activity of RuO_*x*_@UiO-66(Zr/Ti)-NO_2_ is about 3 times higher than this photocatalyst under similar reaction conditions. Furthermore, RuO_*x*_@UiO-66(Zr/Ti)-NO_2_ exhibits an activity 3–6 times higher than that achieved using analogous solids based on RuO_*x*_ NPs supported on Ti-based MOFs, such as MIL-125(Ti)-NH_2_ (Table S1, entries 3 and 4) or MIP-208(Ti) (Table S1, entry 5). It is remarkable that the activity of RuO_*x*_@UiO-66(Zr/Ti)-NO_2_ (Table S1, entry 1) is more than two times compared with RuO_*x*_@MIL-125(Ti)-NH_2_ (Table S1, entry 4) having double the amount of ruthenium (2 wt %). It should be noted that all these photocatalysts have a similar RuO_*x*_ NP loading (1 wt % of ruthenium) and an average particle size (∼ 2 nm). The higher activity of RuO_*x*_@UiO-66(Zr/Ti)-NO_2_ thus appears to be related to the energy band diagram level of the photocatalyst determined by the combination of 2-nitroterephthalates ligands and mixed-metal Zr(IV)/Ti(IV) metal nodes, which boosts the efficiency of the reaction. Regardless of these comments, it is pertinent to mention that the state-of-the-art in current photocatalytic gaseous methanation has reported activities, in some cases, greater than 100 mmol g^–1^ h^–1^. In one of these examples, ultrathin Mg–Al layered double hydroxide nanosheet supported Ru NPs were found to achieve efficient photothermal CO_2_ methanation (277 mmol h^–1^ g^–1^; 300 W Xe lamp) under continuous flow operation.^69^

**Figure 5:**
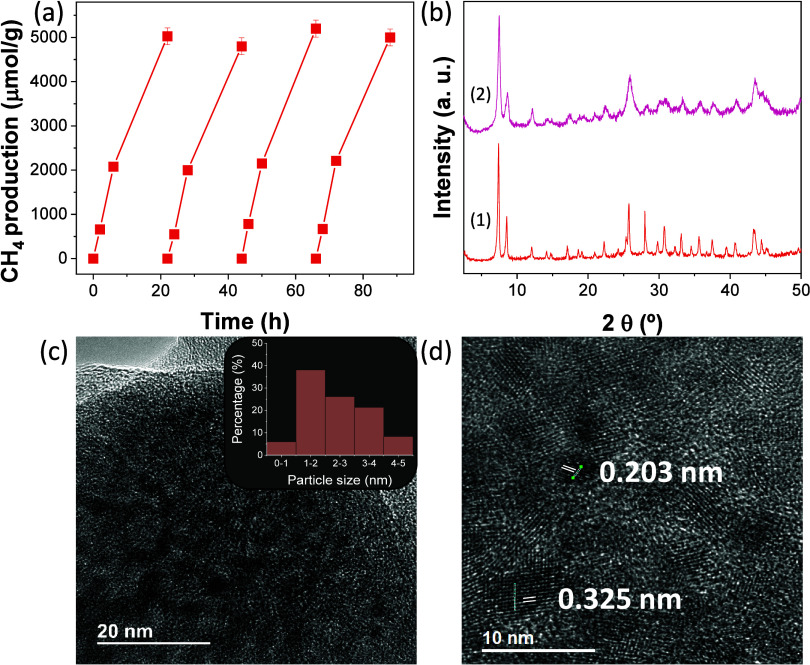
(a) Reusability of RuO_*x*_@UiO-66(Zr/Ti)-NO_2_ during photocatalytic CO_2_ methanation. (b) PXRD of RuO_*x*_@UiO-66(Zr/Ti)-NO_2_ fresh (1) and used (2). (c) TEM image and particle size distribution of used photocatalyst. (d) HRTEM for interplanar distance.

The activity and stability of RuO_*x*_@UiO-66(Zr/Ti)-NO_2_ were studied by performing consecutive reuse experiments. Figure 5 shows that the photocatalyst can be reused without significant loss of activity for four consecutive times with an accumulated reaction time of 90 h. According to PXRD analysis, the crystallinity of the four-times used photocatalyst is preserved. TEM analyses of the reused photocatalyst confirmed that RuO_*x*_ average particle size and standard deviation (2.32 ± 0.90 nm) are similar compared to the fresh sample (2.08 ± 0.82 nm). Besides, HR-TEM characterization of the used photocatalyst revealed the presence of lattice fringes with spacings of about 0.203 and 0.32 nm, which were ascribed to the crystal planes (101) and (110) of Ru(0) and RuO_2_, respectively (Figures 5d and S39).

**Figure 6:**
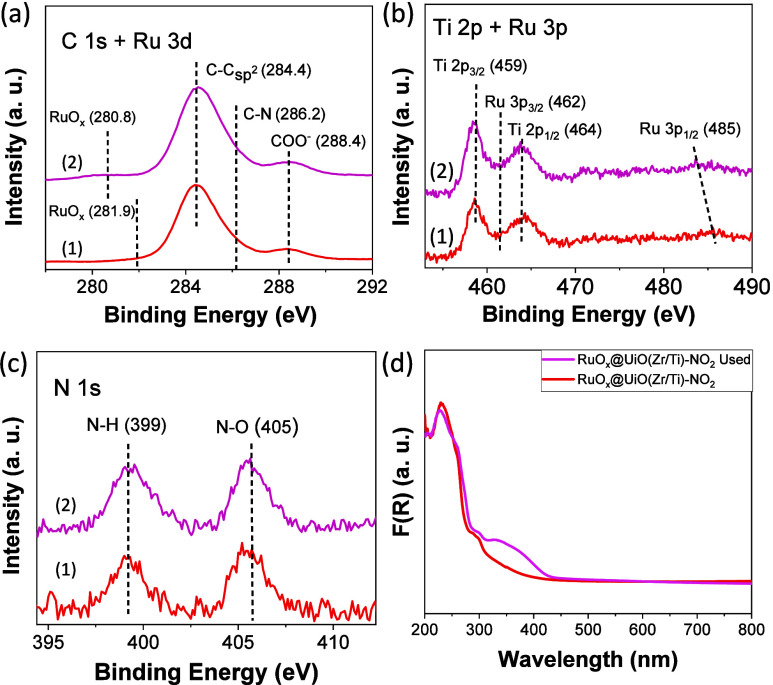
(a) C 1s + Ru 3d, (b) Ti 2p + Ru 3p, (c) N 1s XPS of fresh (1) and used (2) photocatalyst, and (d) UV–vis of fresh and used RuO_*x*_@UiO-66(Zr/Ti)-NO_2_.

C 1s, O 1s, Zr 3d, and Ti 2p XPS analyses of the four-times used photocatalyst (Figure S40) showed similar features to those of the fresh material, whereas N 1s and Ru 3d XPS showed small but appreciable differences with respect to the fresh sample (Figure 6 and Figure S41). N 1s XPS of the used photocatalyst revealed slight hydrogenation of the nitro group to the amino group (Figure 5). Specifically, the fresh and used RuO_*x*_@UiO-66(Zr/Ti)-NO_2_ photocatalysts have a proportion in weight percent of NO_2_ versus NH_2_ of 55.2/44.8 and 46.8/53.2, respectively. Although partial reduction of NO_2_ to NH_2_ is observed in the used RuO_*x*_@UiO-66(Zr/Ti)-NO_2_ photocatalyst by XPS, the structural integrity of the used photocatalyst still contains enough NO_2_ groups (46.8 at%) to promote the photocatalyst activity without much significant difference (Figure 6). Furthermore, UV–vis DRS of the used sample showed an extra absorption band with onset absorption at about 430 nm, which agrees with the partial nitro hydrogenation to the amino group (Figure 5). In the case of Ru 3d XPS, a small shift of the Ru 3 d_5/2_ was seen toward lower binding energies with respect to the fresh sample (281.9 vs 280.8 eV). These results agree with previous studies that also showed the supported RuO_*x*_ NPs employed as cocatalysts during (photo)catalytic hydrogenations at temperatures of about 200 °C can be converted to some extent to the metallic phase.^44,69−72^

In the present study, additional *in situ* XPS experiments in which the fresh RuO_*x*_@UiO-66(Zr/Ti)-NO_2_ sample is submitted to a H_2_ thermal treatment at 200 °C also revealed that supported RuO_*x*_ NPs are susceptible to be partially reduced to metallic NPs under the studied reaction conditions (Figure S42). It should be noted that metallic ruthenium species have been proposed as responsible species to activate molecular H_2_ and initiate CO_2_ hydrogenation.^69,70,72,73^ Besides, as will be shown later, RuO_*x*_ and Ru species also favor CO_2_ and CO chemisorption as evidenced by FTIR spectroscopy. Overall, these results demonstrate that RuO_*x*_ NPs supported on UiO-66(Zr/Ti)-NO_2_ are partially reduced during the photocatalytic CO_2_ hydrogenation process, leading to the coexistence of supported RuOx and Ru(0) species within the photocatalyst.

In the area of photocatalysis using MOFs, some studies have reported UV–vis irradiation of carboxylate-based MOFs at 200 °C that resulted in partial decarboxylation.^74^ To address this issue, a photocatalytic control experiment in which CO_2_ was replaced by Ar revealed the presence of CO_2_, attributed to the partial decarboxylation of the terephthalate MOF ligand during the reaction (1.8 wt % with respect to the amount of the initial carboxylate). These results indicate a need to develop active MOF-based photocatalysts that can operate under milder reaction conditions with operational stabilities.

### 3.3 Photocatalytic Reaction Pathways

#### 3.3.1 Exploration of Photochemical and Photothermal Reaction Mechanisms

Based on previous reports, photocatalytic CO_2_ reduction using metal/metal oxide NPs supported on MOFs or other materials can occur via photochemical^24,34^ and/or photothermal reaction mechanisms.^24,34,75−77^ During the photochemical pathway, the irradiation of the photocatalysts results in the formation of reducing and oxidizing electron and hole pairs, respectively. This is a common reaction mechanism found when using MOFs as photocatalysts when their irradiation by appropriate wavelengths produces photoinduced electron transfer from the organic ligand to the metal node.^43^ The presence of MNPs like RuO_*x*_ as cocatalysts can also favor photochemical pathway efficiency by opening new channels for charge carrier separation and enhancing photocatalytic activity.^44^ RuO_*x*_ NPs have also been reported to promote the photothermal reaction pathway in which light energy is transformed into heat, which favors CO_2_ methanation.^75^

Several characterization techniques were used to further study these possible reaction pathways using RuO_*x*_ NPs supported UiO-66(Zr and/or Ti)-X (X: NH_2_ or NO_2_). It should be noted that, as shown in Figure 6, the RuO_*x*_@UiO-66(Zr/Ti)-NO_2_ photocatalyst used exhibits a partial reduction of supported RuO_*x*_ NPs with respect to the fresh sample. To consider the possible influence of the RuO_*x*_ oxidation state on the subsequent characterization data, some comparative measurements were carried out using both fresh and used photocatalysts.

**Figure 7:**
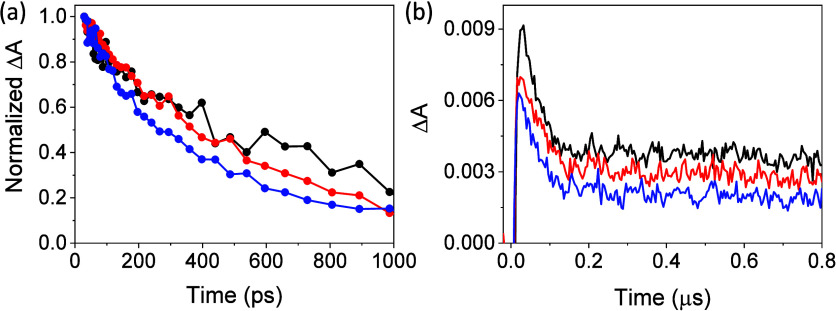
(a) Femtosecond transient absorption recorded at 586 nm and (b) LFP decay traces recorded at 520 nm for UiO-66(Zr)-NO_2_ (black), UiO-66(Zr/Ti)-NO_2_ (red), and RuO_*x*_UiO-66(Zr/Ti)-NO_2_ (blue). fs-TAS measurements were performed at λ_exc_ = 267 nm in aerated MeCN, whereas those of LFP were done at λ_exc_ = 266 nm in MeCN under an Ar atmosphere.

To evaluate the photoinduced processes arising from the excitation of the different UiO-66(Zr/Ti)-X (X: NH_2_ or NO_2_) photocatalysts at 267 nm,^30,75^ these were first studied by femtosecond TAS (fs-TAS). This technique has been shown to be sensitive and precise for investigating processes occurring at a very early stage after excitation, including ultrafast electron transfer or charge separation.^78^ The recorded transient absorption spectra (Figure S43) and kinetics (Figure S44) of UiO-66(Zr)-NH_2_ showed good agreement with previously reported observations,^79^ whereas notable differences were found in the transient absorption spectra when using NO_2_ (Figure S45). The transient absorbance of the latter samples covers the entire visible spectrum and does not exhibit any remarkable band/feature (Figure S45). A set of the kinetic traces ranging from 550 to 750 nm were analyzed by means of a global fit, including two-time constants, to describe the dynamics during the first nanoseconds after photoexcitation. Table S2 includes the resulting time constants for all the species studied. The fastest components (of the order of a few tens of picoseconds) were associated with electron transfer processes from HOCO to LUCO of MOFs,^79^ wheresa the longer-lived components, which remained up to the nanosecond time scale, were assigned to a deep trap state.^80^Figures 7a shows for nitro-functionalized UiO-66 solids a comparison of the transients together with the average lifetimes calculated for each probe wavelength on the basis of the time constants derived from the global fit. The data reveal that the fastest relaxation dynamics is that of RuO_*x*_@UiO-66(Zr/Ti)-NO_2_ followed by an analogous mixed-metal UiO-66(Zr/Ti)-NO_2_ parent sample, whereas monometallic UiO-66(Zr)-NO_2_ exhibited longer-lived components. Similar conclusions can be drawn for amino-functionalized UiO-66 materials (Figure S44). In this regard, kinetic traces have been used as indicators to evaluate electron–hole separation efficiency of the photocatalysts. It is therefore proposed, by means of comparisons with previous ultrafast results from related MOFs,^79^ that the faster the relaxation dynamics is, the higher is the charge-separation efficiency. In fact, the order of photocatalytic activity in our case agrees, to some extent, with the relaxation trace kinetics using ultrafast TAS measurements.

Long-lived trap states for UiO-66 photocatalysts were further investigated on longer time scales by the laser flash photolysis (LFP) technique at λ_exc_ = 266 nm. The spectra obtained for the different nitro- (Figure 7b and Figure S46) and amino- (Figure S47) functionalized UiO-66 photocatalysts in an Ar atmosphere on the nanosecond time scale were characterized by a continuous absorption band from 300 to 750 nm. Previous TAS studies by some of us using UiO-66(Zr)-X (X: NH_2_ or NO_2_) assigned these transient absorption bands to photogenerated electron and holes based on selective quenching experiments.^51,54^ Similar conclusions have been obtained in the present case using methanol as hole quencher for the series of amino-functionalized UiO-66 solids. Figure S48 shows that methanol quenches the region from 300 to 400 nm, resulting in a parallel increase of the transient signals around 600 nm, which indicates that hole deactivation enhances the yield of photogenerated electrons, an effect previously found in other related MOF-based photocatalysts.^81,82^ These results agree with those obtained from ultrafast TAS and demonstrate the photogeneration of charge separation species as electrons and holes. In line with the ultrafast results, LFP decay traces at 400 and 680 nm show that the faster the decay components are (see Table S2), the higher is the photocatalytic activity of all the studied RuO_*x*_ NPs supported UiO-66(Zr/Ti)-X (X: NH_2_ or NO_2_) in their series. In short, in terms of photocatalyst decay relaxation dynamics, both fs- and ns-TAS serve as indicators of charge separation efficiency and agree with the order observed in their photocatalytic activity.

**Figure 8:**
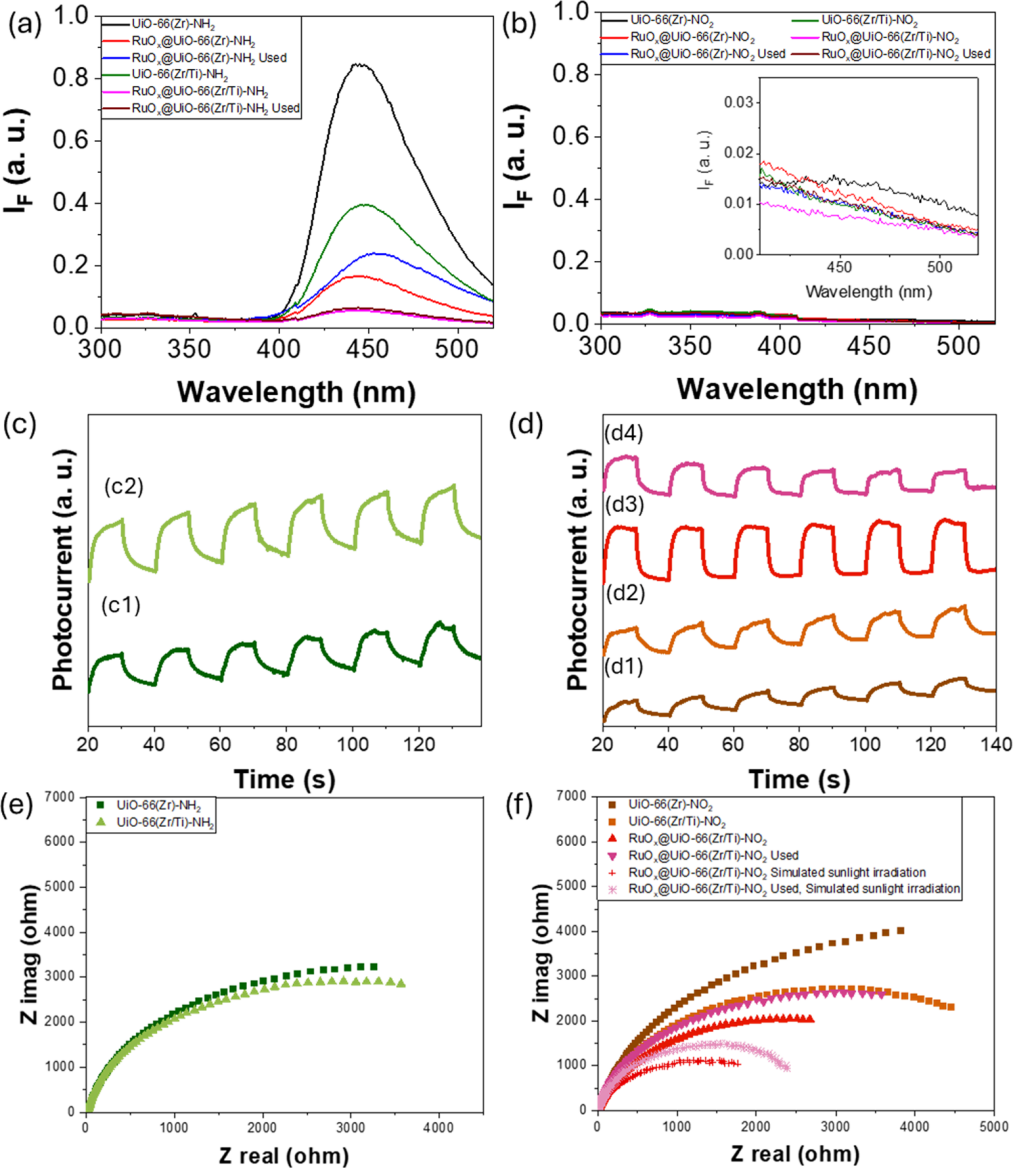
(a) PL measurements performed in acetonitrile MOF suspension having the same optical absorption (*ca.* 35 au) at 266 nm corresponding with the monochromatic excitation wavelength of the MOF organic. (b) Current intensity response of amino- (c) or nitro-based (d) UiO-66 solids. Nyquist plots of amino- (e) or nitro-based (f) UiO-66 solids under dark or simulated concentrated sunlight irradiation as indicated. Legend: (c1) UiO-66(Zr)-NH_2_, (c2) UiO-66(Zr/Ti)-NH_2_, (d1) UiO-66(Zr)-NO_2_, (d2) UiO-66(Zr/Ti)-NO_2_, (d3) RuO_*x*_@UiO-66(Zr/Ti)-NO_2_ fresh, and (d4) RuO_*x*_@UiO-66(Zr/Ti)-NO_2_ used.

To further evaluate the photoinduced charge separation efficiency of UiO-66 solids and their relationship with their photocatalytic activities, photocatalysts were characterized by PL spectroscopy and transient photocurrent and EIS measurements. PL spectroscopy is commonly used in heterogeneous photocatalysis, including MOFs, to evaluate the photoexcited charge transfer and recombination processes.^83,84^ Amino functionalized UiO-66 solids have a different degree of fluorescence, whereas negligible emission was found when using the nitro-functionalized solids. These results agree with some of our previous results showing that acetonitrile solutions of 2-aminoterephthalate emit much more on excitation at 266 nm than the analogous 2-nitroterephthalate acetonitrile solutions.^54^Figure 8a shows that the UiO-66(Zr/Ti)-NH_2_ suspension has lower emissions than UiO-66(Zr)-NH_2_, which agrees with similar studies that highlighted the higher efficiency of photoinduced charge separation of mixed-metal UiO-66(Zr/Ti)-NH_2_ solids, in which Ti(IV) atoms act as the electron mediator during the process.^48^ Similar measurements using fresh or used RuO_*x*_ NPs supported UiO-66(Zr)-NH_2_, and specially UiO-66(Zr/Ti)-NH_2_ solids, produced considerably less fluorescence emission intensity. Regardless of the much lower fluorescence emission intensity observed when using nitro-functionalized UiO-66-based solids compared to amino ones, analogous conclusions about the fluorescence quenching in mixed-metal solids with or without fresh and used RuOx with respect to the parent sample can be drawn (Figure 8b). These results indicate that the presence of RuO_*x*_ NPs in the UiO-66 solids reduces the recombination rate of photogenerated electron–hole pairs and thus increases the efficiency of photoinduced charge separation.

The transient photocurrent results using UiO-66 solids under several on/off illumination cycles are shown in Figure 8. For these measurements, UiO-based photocatalysts were supported on a carbon substrate electrode and used in a standard three-electrode electrochemical cell as a working electrode previously polarized at +0.9 V. The results show that mixed-metal UiO-66 solids have higher photocurrent intensities than monometallic ones (Figure 8). Analogous measurements using used and fresh RuO_*x*_@UiO-66(Zr/Ti)-NO_2_ photocatalysts found higher current intensities in simulated concentrated sunlight illumination and indicated an improvement in charge separation efficiency. An additional experiment using fresh RuO_*x*_@UiO-66(Zr/Ti)-NO_2_ in the presence of methanol gave a fivefold enhancement of current intensity (Figure S49). This was due to the oxidation of methanol in the photogenerated holes that partially avoided electron recombination so that a higher current intensity was measured than in the experiment with pure acetonitrile as solvent.

PL and transient photocurrent conclusions were complemented by EIS measurements (Figure 8). The smallest Nyquist arc radii were obtained from the most active samples of the series with the lowest charge transfer resistance. PL, transient photocurrent, and EIS measurements showed that titanium ions in the metal nodes of UiO-66(Zr/Ti) and/or RuO_*x*_@UiO-66 solids acted as electron mediators during the photoinduced electron transfer from the organic ligand to the metal node and increased the process efficiency.^48,84^

**Figure 9:**
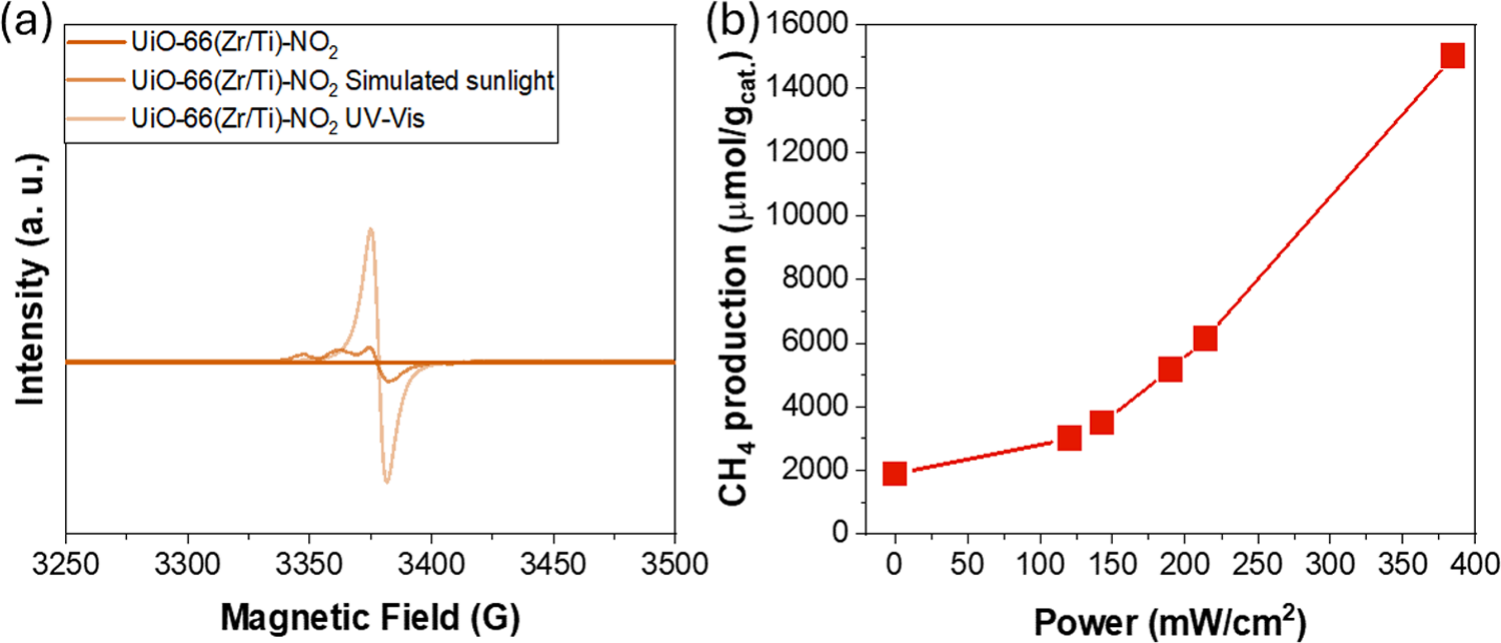
(a) EPR of solid UiO-66(Zr/Ti)-NO_2_ after different irradiation conditions as indicated. (b) Influence of simulated light intensity into photocatalytic CH_4_ formation.

Previous studies reported the use of solid-state ESR spectroscopy to characterize the formation of photoactive reductive sites in MOFs like UiO-66(Zr)-NH_2_^54,58^ or MIL-125(Ti)-NH_2_. For example, it has been reported that irradiation of UiO-66(Zr)-NH_2_ results in photoinduced charge separation from the organic ligand to the metal node and the transformation of Zr(IV) species into Zr(III) species while the holes are located in the organic ligand.^54,58,85^ Other studies have proposed that the irradiation of mixed-metal UiO-66(Zr/Ti)-NH_2_ produces an LMCT mechanism with the initial reduction of Ti(IV) to Ti(III) in Ti(III)-O-Zr(IV) metal nodes, which are later transformed into Ti(IV)-O-Zr(III).^48^ These studies highlight the role of Ti(IV) species in mixed-metal UiO-66 solids as electron mediators from excited organic ligands that favor charge separation. In the present study, solid-state ESR experiments were carried out using UiO-66(Zr)-X (X: NH_2_ or NO_2_) and the analogous mixed-metals UiO-66(Zr/Ti)-X (X: NH_2_ or NO_2_) (Figure 9 and Figure S50). Control solid-state ESR experiments in dark conditions revealed the presence of some paramagnetic signals in amino-functionalized UiO-66 associated with the presence of Zr(III) species that, however, are absent in analogous nitro solids, in agreement with previous related studies.^54^ Irradiation of mono- or bimetallic UiO-66 solids functionalized with either amino or nitro groups in all cases produced the formation of an ESR band with *g* value of 2.004, characteristic of the Zr(III) species. These experiments indicate the occurrence of LMCT mechanisms in MOFs, whereas the absence of ESR Ti(III) signals could be associated with the previously proposed fast kinetics of metal electron transfer from Ti(III) as electron mediator to geminal Zr(IV).^48^

Based on previous studies and due to the use of RuO_*x*_ NPs as cocatalysts during photocatalytic CO_2_ reduction, the occurrence of a photothermal pathway can be hypothesized in which light energy is transformed into heat energy.^75−77^ An indirect experiment to determine this possible pathway was conducted by evaluating photocatalytic CO_2_ methanation as a function of the simulated sunlight intensity. Figure 9 shows that photocatalytic methane production increases linearly as a function of irradiance intensity up to about 125 mW/cm^2^, and then an exponential relationship can be seen. These results are interpreted as the occurrence of a photothermal reaction pathway, especially at high irradiance intensities, in which light irradiation is transformed into local heat in RuO_*x*_ NPs, promoting CO_2_ hydrogenation to CH_4_.

The measurement of catalyst temperature during the photothermal reaction is of great importance to understand the thermal- and nonthermal contributions of the whole process.^86^ To address this challenging measurement, several techniques have been reported like direct measurement with a thermocouple or noncontact techniques with infrared sensors or thermal cameras.^86^ Another common method to assess catalyst local heating is based on the use of supported inorganic quantum dots (QDs) as temperature sensors with optical readout.^26,87^ Specifically, the measurement of PL emission decrease of supported QDs on a photocatalyst is a function of the local temperature.^26,43^ In this work, commercially available CdSe-ZnS QDs were employed as local nanothermometers. A series of PL experiments upon CdSe-ZnS QDs excitation at 450 nm were performed at temperatures from 200 to 280 °C under dark or upon simulated sunlight irradiation intensities from 85 to 385 mW/cm^2^. Figure S51 shows that the characteristic PL emission band of CdSe-ZnS QDs centered about 540 nm gradually decreases as the temperature increases. These experiments confirmed the possibility of using these CdSe-ZnS QDs as local nanothermometers, in agreement with previous reports.^26,43^ Additionally, the PL emission intensity of CdSe-ZnS QDs recorded at 200 °C also decreased upon irradiation, the highest the irradiation intensity the highest the PL quenching, a fact associated with the local heating of CdSe-ZnS QDs upon irradiation. Analogous PL results were obtained in the case of used RuO_*x*_@UiO-66(Zr/Ti)-NO_2_ photocatalyst supported CdSe/ZnS QDs deposited on a quartz holder as a function of either the temperature or the simulated sunlight irradiation at different irradiances. It should be noted that during these PL experiments, negligible temperature changes of the sample upon different irradiations were measured using an infrared thermometer. Therefore, it is likely to propose that the observed PL quenching upon irradiation might be associated with a photocatalyst local heating (*ca.* to about 220 or 280 °C as a function of the irradiance; Figure S52) due to irradiation. Overall, these PL results together with those ones shown in Figure 9 about the influence of simulated light intensity into photocatalytic CH_4_ formation would agree with the occurrence of a photothermal reaction pathway during CO_2_ reduction.

**Figure 10:**
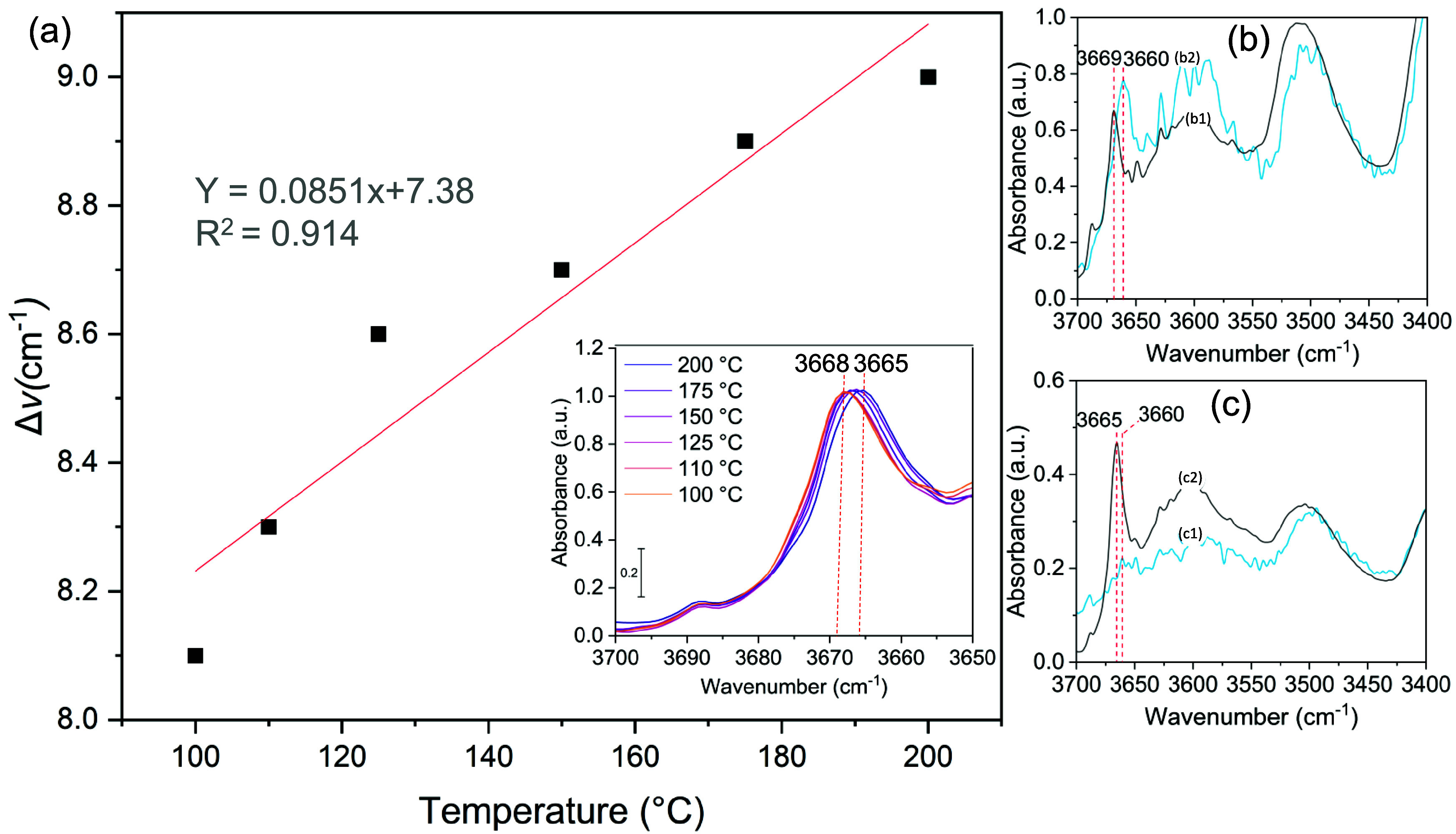
(a) Evolution of the structural band shift as a function of temperature (inset: direct surface FTIR spectra of RuO_*x*_@UiO-66(Zr/Ti)-NO_2_ in the 3700–3650 cm^–1^ region at different temperatures in the dark). (b, c) FTIR spectra in the same region (1) in the dark and (2) under irradtion at 30 and 200 °C, respectively. Spectra collected under continuous flow of Ar (20 cm^3^/min).

Further investigation of the photothermal behavior was conducted by monitoring the IR band shift of the structural bands using operando FTIR experiments under different temperatures given the fact that as temperature increases, the molecular vibration of the different species increases, resulting in a shift in IR bands.^88,89^ Results confirm this behavior with RuO_*x*_@UiO-66(Zr/Ti)-NO_2_ showing a structural band shift from 3668 to 3664 cm^–1^ (assigned to OH vibration) as temperature increased from 100 to 200 °C under dark conditions (inset of Figure 10a). This effect was then investigated for the reaction under irradiation at room temperature. Interestingly, a significant band shift from 3369 to 3360 cm^–1^ (Figure 10b) is observed indicating a potential localized temperature increase (estimated at around 145 °C). However, at an elevated temperature (200 °C), this effect was diminished, whereby a lower band shift is observed (Figure 10c). This result is consistent with that of the PL quenching with QDs.

**Figure 11:**
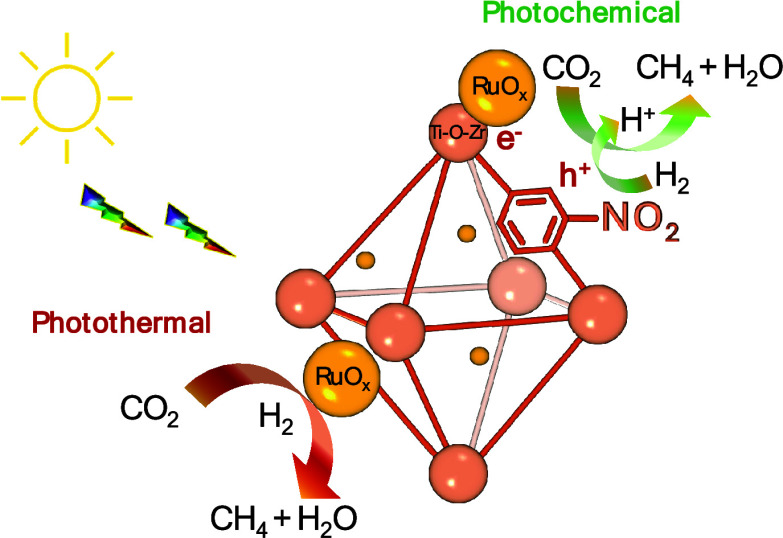
Proposed reaction mechanism during CO_2_ methanation using RuO_*x*_@UiO-66(Zr/Ti)-NO_2_ as multifunctional photocatalyst.

Overall, RuO_*x*_ NPs supported UiO-66(Zr/Ti)-NO_2_ generally acts as a multifunctional photocatalyst during CO_2_ methanation under simulated concentrated sunlight irradiation (Figure 11). During the photochemical pathway irradiation of the photocatalyst, we consider that a photoinduced electron transfer from the organic ligand to the metal-oxo cluster takes place. These electrons can be further transferred to RuO_*x*_ NPs, where CO_2_ methanation occurs. Irradiation can also promote the heating of RuO_*x*_ NP and CO_2_ hydrogenation to methane.

#### 3.3.2 Evaluation of Photocatalytic CO_2_ Hydrogenation to CH_4_

**Figure 12:**
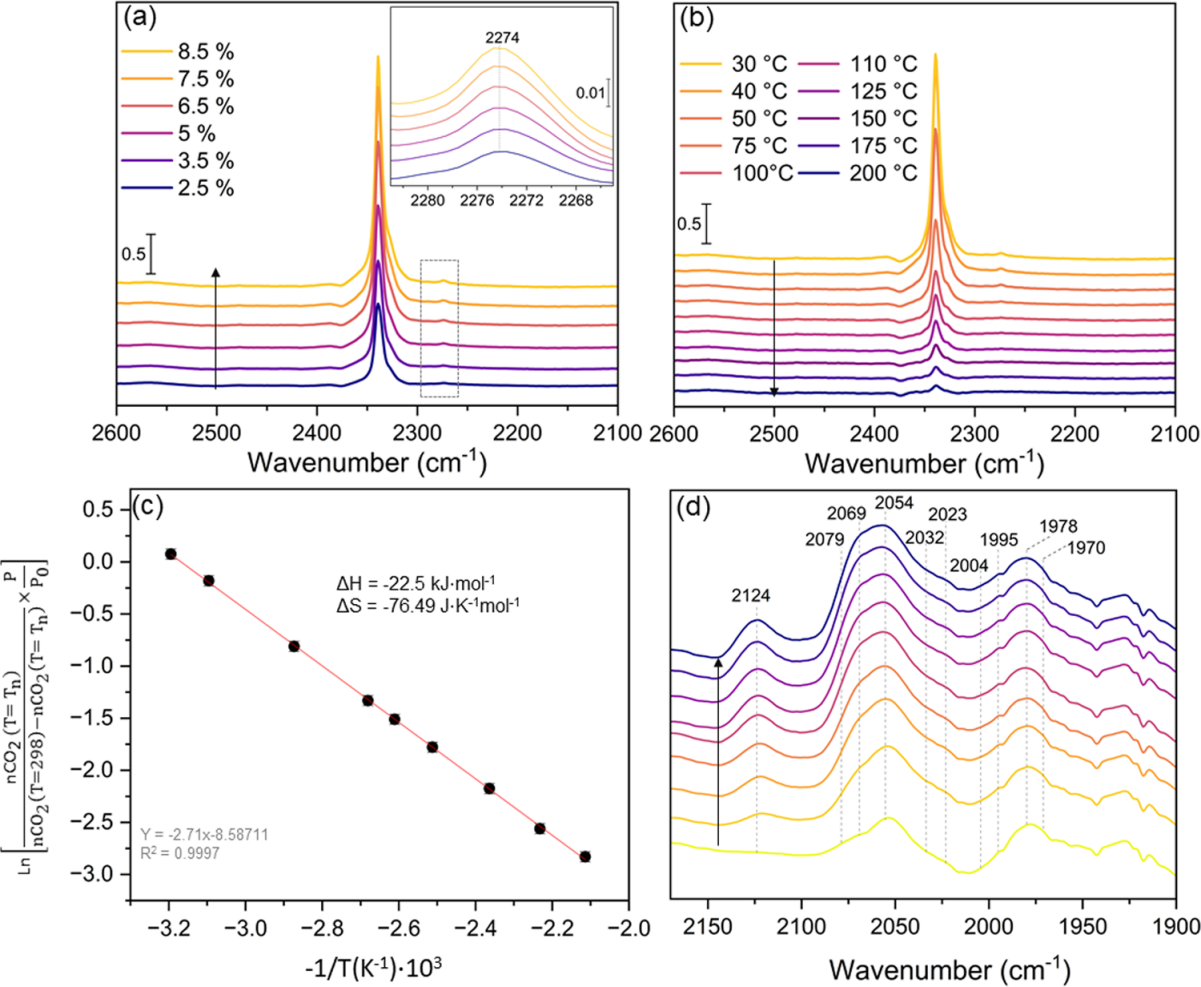
(a, b) FTIR spectra of chemisorbed CO_2_ on RuO_*x*_@UiO-66(Zr/Ti)-NO_2_ versus different concentrations of CO_2_ in argon and temperatures, respectively (total flow rate = 20 cm^3^·min^–1^); (c) the corresponding enthalpy and the entropy of the CO_2_ chemisorption; and (d) evolution of FTIR spectra of adsorbed CO on RuO_*x*_@UiO-66(Zr/Ti)-NO_2_ in 2150–1900 cm^–1^.

The previous photocatalytic results using UiO-66-based materials have shown a selective CO_2_ hydrogenation to CH_4_. These results agree with analogous studies reporting that the use of supported RuO_*x*_ NPs facilitates chemisorption of CO_2_ and their reaction intermediates and promote the (photo)catalytic hydrogenation to methane.^90−92^ To shed some light on the CO_2_ and CO adsorption capacity aver RuO_*x*_@UiO-66(Zr/Ti)-NO_2_ topic, CO_2_ and CO adsorption experiments were conducted under continuous flow of CO_2_/Ar and CO/Ar under operando conditions, and the results were analyzed through FTIR^93,94^ Initially, different concentrations of CO_2_ were introduced in Ar with a total flow rate of 20 cm^3^·min^–1^. It is witnessed that as the concentration of CO_2_ increases, the band centered at 2238 cm^–1^ characteristic of chemisorbed CO_2_ increases in a linear way, as illustrated in Figure 12a. The results are presented after subtraction of gaseous CO_2_ phase. Direct spectra can be found in the SI (Figure S53a). Upon reaching saturation, CO_2_ adsorption was investigated as a function of the temperature (as depicted in Figure 12b). These results demonstrated an exponential decrease in chemisorbed CO_2_ concentration as the temperature increases, until reaching 30 °C. Subsequently, the enthalpy and entropy of this reaction were calculated based on the linear relationship of  (where *n*CO_2_ represents the number of moles of chemisorbed CO_2_, *T*_n_ is the temperature reached at each point, and *P*/*P*_0_ is the relative pressure of CO_2_ in Ar) as a function of the inverse of temperature (−1/*T*), as shown in Figure 12c. The enthalpy of the reaction was determined from the slope of the line and equal to −22.5 kJ/mol, indicating relatively weak and reversible adsorption of CO_2_ on the catalyst surface. The investigation of CO adsorption on supported ruthenium catalysts holds significance not only for understanding the mechanism of CO_2_ methanation reaction (considering that CO is one of the potential intermediates of this reaction) but also for the characterization of their surface properties. CO serves as a prominent probe molecule, unveiling both the oxidation state and coordination environment of the sites to which it binds. Figure 12d shows the evolution of the FTIR spectra of the RuO_*x*_@UiO-66(Zr/Ti)-NO_2_ in the CO vibration spectral region (2200–1800 cm^–1^) upon the introduction of 0.05% CO in Ar to the sample preactivated under hydrogen at 200 °C. The results are subtracted from the spectrum after activation at room temperature, and the direct spectra can be found in the Figure S53b. Different bands appeared on the surface; however, their assignment to specific adsorption sites is not straightforward, as witnessed by the different interpretations found in the literature. This variability likely arises from multiple factors influencing exact band positions, such as the coverage of CO, as well as the oxidation state of the adsorbant. According to literature findings, carbonyl species can predominantly be categorized into two distinct surface complexes: the spectral peaks at 2124 cm^–1^, coupled with a component at 2055 cm^–1^, are attributed to the asymmetric and symmetric stretching vibrations of a Ru^3+^(CO)_2_ species on the proximity of ZrO_2_.^95^ Meanwhile, the spectral peaks at 2070 and 2004 cm^–1^ are associated with those of Ru^2+^(CO)_2_ species.^89^ Furthermore, peaks at lower wavenumbers (1995, 1987, and 1955 cm^–1^) may be attributed to monocarbonyls adsorbed on less oxidized Ru^δ+^ supported on TiO_2_. Furthermore, a less intense peak at 2023 cm^–1^ may be attributed to CO linearly bound to metallic Ru^0^. The approximative assignments of the spectral bands are summarized in Table S3.

**Figure 13:**
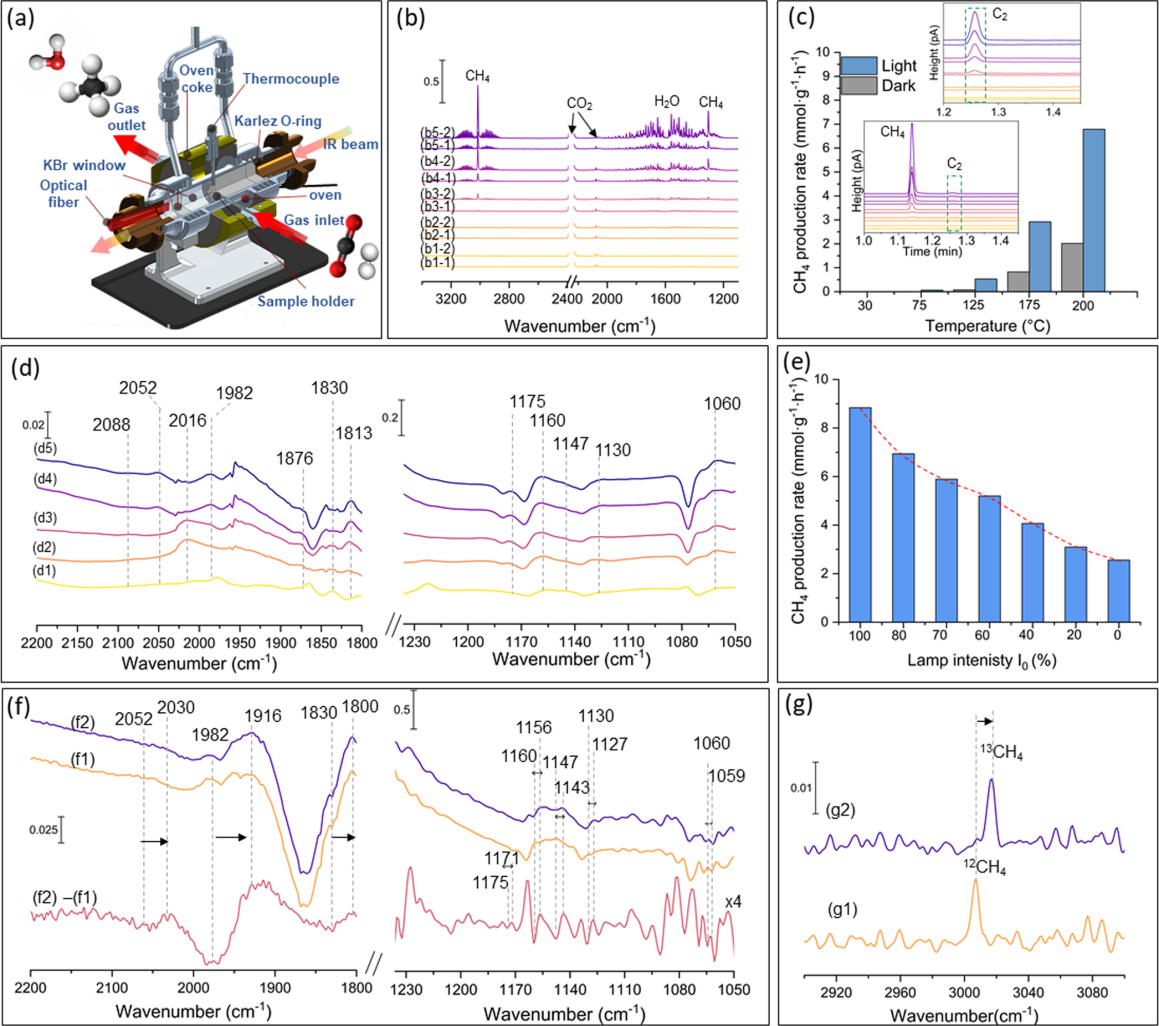
(a) Sandwich IR reactor-cell used for studying the CO_2_ methanation reaction under visible light in continuous flow. (b) The FTIR spectra of the reaction gas phase: (b1) 30, (b2) 75, (b3) 125, (b4) 175, and (b5) 200 °C (-1) in the dark and (-2) after irradiation at steady state. (c) Activity of RuO_*x*_@UiO-66(Zr/Ti)-NO_2_ as a function of temperature in the dark and under visible light irradiation (inset: gas chromatograms of the reaction gas phase under the same conditions). (d) Operando FTIR spectra of RuO_*x*_@UiO-66(Zr/Ti)-NO_2_ versus temperature: (d1) 30, (d2) 75, (d3) 125, (d4) 175, and (d5) 200 °C in the 2200–1800 and the 1200–1000 cm^–1^ vibrational regions. (e) Activity of RuO_*x*_@UiO-66(Zr/Ti)-NO_2_ as a function of lamp intensity. (f) FTIR spectra of the catalyst during the photoassisted methanation at 200 °C of (1) ^13^CO_2_ and (2) ^12^CO_2_; the corresponding subtracted spectrum (^13^CO_2_ – ^12^CO_2_) presented in (d3) (intensity was multiplied by 4 for clarity. (g) FTIR gas phase spectra of gaseous methane produced during the photoassisted methanation of (1) ^12^CO_2_ and (2) ^13^CO_2_ at 200 °C. The arrow corresponds to the shift of the IR bands due to the isotopic exchange from ^12^CO_2_ to ^13^CO_2._ The assignments of the different IR bands are summarized in Tables S3 and S4.

The performance of the most active RuO_*x*_@UiO-66(Zr/Ti)-NO_2_ sample during photocatalytic CO_2_ hydrogenation to CH_4_ was further investigated through operando FTIR experiments where both the gas phase products and the surface of the catalyst are analyzed simultaneously in real time under similar irradiation conditions used previously in batch.^96−98^ The setup is equipped with an online mass spectrometer (MS) and gas chromatography (GC) whereby online injections are taken throughout the reaction. A “Sandwich” cell reactor (Figure 13a) was used to carry out these experiments where the catalyst is fixed in the cell as a self-supported pallet (20 mg). The sample was first activated under hydrogen at 200 °C, and then its activity for the CO_2_ methanation was assessed with a molar ratio of 4:1 of H_2_ to CO_2_ with a total flow rate of 10 cm^3^·min^–1^. The photothermal CO_2_ methanation activity of RuO_*x*_@UiO-66(Zr/Ti)-NO_2_ was tested under different temperatures (Figure 13b,c). No methane production was detected at 30 °C either in darkness or under visible light irradiation, as evidenced by the analysis of the gas phase at the steady state. However, upon reaching 75 °C, methane production increased with rising temperature in the absence of light. Interestingly, under irradiation, methane production exhibited a significant increase with increasing temperature, reaching 8 mmol·g^–1^·h^–1^ at 200 °C, with a selectivity of 98.3%. This observation was further confirmed by FTIR analysis, which revealed only CH_4_ and H_2_O as gas phase products (Figure 13b). Complementary results of the GC analysis showed, in addition to methane, the production of ethane (under the detection limit of our FTIR-gas analysis) as a side product with a selectivity of 1.7% (inset of Figure 13c). Subsequently, an investigation into the impact of lamp intensity on the activity of RuO_*x*_@UiO-66(Zr/Ti)-NO_2_ was carried out at 200 °C, as illustrated in Figure 13e. The sample’s activity decreased in quasi-linear manner from 8.7 to 3.5 mmol·g^–1^·h^–1^ as the relative intensity of the lamp decreased from 100 to 20% I_0._ No significant deactivation was observed, in agreement with the previous experiments that were conducted in batch conditions at different simulated sunlight irradiation intensities. The decline in the sample’s activity with decreasing lamp intensity suggests a diminished prominence of plasmonic effects during the reaction under irradiation. This indicates that the catalytic behavior may be governed by factors beyond predominant plasmonic-mediated mechanisms.

In an attempt to gain more information on the underlying mechanism of the CO_2_ hydrogenation over RuO_*x*_@UiO-66(Zr/Ti)-NO_2_, the surface of the catalyst was simultaneously monitored by FTIR during the reaction. The FTIR spectra of the surface, at steady state, between 30 and 200 °C are shown in Figure 13d. Results are subtracted from the spectrum at 30 °C of the preactivated sample. It is essential to note that the spectral regions corresponding to the stretching vibration of formates and carbonates, specifically between 1600 and 1300 cm^–1^, are saturated. Therefore, for assigning the different possible reaction intermediates, both the CO vibrational region and the unobstructed region between 1200 and 1000 cm^–1^ are taken into consideration. Different bands emerged as temperature increased in the CO region mainly at 2088 and 1985 cm^–1^ possibly attributed to adsorbed CO on Ru ^δ+^ on the proximity of TiO_2_.^89^ Furthermore, a band emerged as temperature increased from 30 to 75 °C at 2016 cm^–1^, after which it diminished. This decrease was accompanied by the start of the methane production at 75 °C (Figure 13b curve b4). This band could be attributed to CO linearly adsorbed on Ru^0^. These results indicate that the formation of CO on the surface is promoted at lower temperatures even if no methane is produced yet. CO in the gas phase was not detected at high temperatures, which emphasizes its key role as an intermediate in the CO_2_ methanation reaction over RuO_*x*_@UiO-66(Zr/Ti)-NO_2_. At higher temperatures, the formation of formyl as an intermediate was suggested by the presence of the band at 1175 cm^–1^.^99^ Furthermore, various bands corresponding to methoxy species are observed in the spectra at 1160 (on-top) and 1060 (doubly bridging) cm^–1^ owing to the stretching vibrations of methoxys.^100^ Also, a band at 1130 cm^–1^ elevated as temperature increased, probably attributed to dioxymethylene adsorbed on the surface.^101^ It is important to mention that increasing the temperature causes a shift in the vibrational bands of species present on the surface,^88^ justifying the negative signals on the subtracted spectra.

To confirm the involvement of the various species in the reaction mechanism and their intermediates role, a steady-state isotopic transient kinetic analysis (SSITKA) experiment using operando FTIR spectroscopy was performed at 200 °C.^102^ It corresponds to replacing ^12^CO_2_ by its isotope ^13^CO_2_ at steady state under the same reaction conditions. The isotopic transient leads to shift of the FTIR bands of the surface intermediates. Additionally, this approach ensures that any observed shift would solely result from isotopic exchange between ^13^CO_2_ and ^12^CO_2_ and not due to the change of temperature and structural band’s shift. However, because of the fact that pure ^13^CO_2_ is very expensive, this experiment was carried out under diluted conditions (1% of ^12^CO_2_ in argon and then exchanged to 1% of ^13^CO_2_ in argon). Interestingly, ^13^CH_4_ was produced selectively with ^13^CO_2_ with a similar quantity to that produced with ^12^CO_2_ under similar conditions (Figure 13g). This was accompanied by the shift (3 or 4 cm^–1^; Table S4) in FTIR bands of different species previously attributed to CO, formyl, methoxy, and dioxymethylene with ^13^CO_2_, as depicted in Figure 13f. Therefore, these observations confirm the role of these species as reaction intermediates. It should be noted that because of the overlap with the CO vibration, it was difficult to distinguish the band related to the hydride bond formed by hydrogen dissociation on reduced Ru.

**Figure 14:**
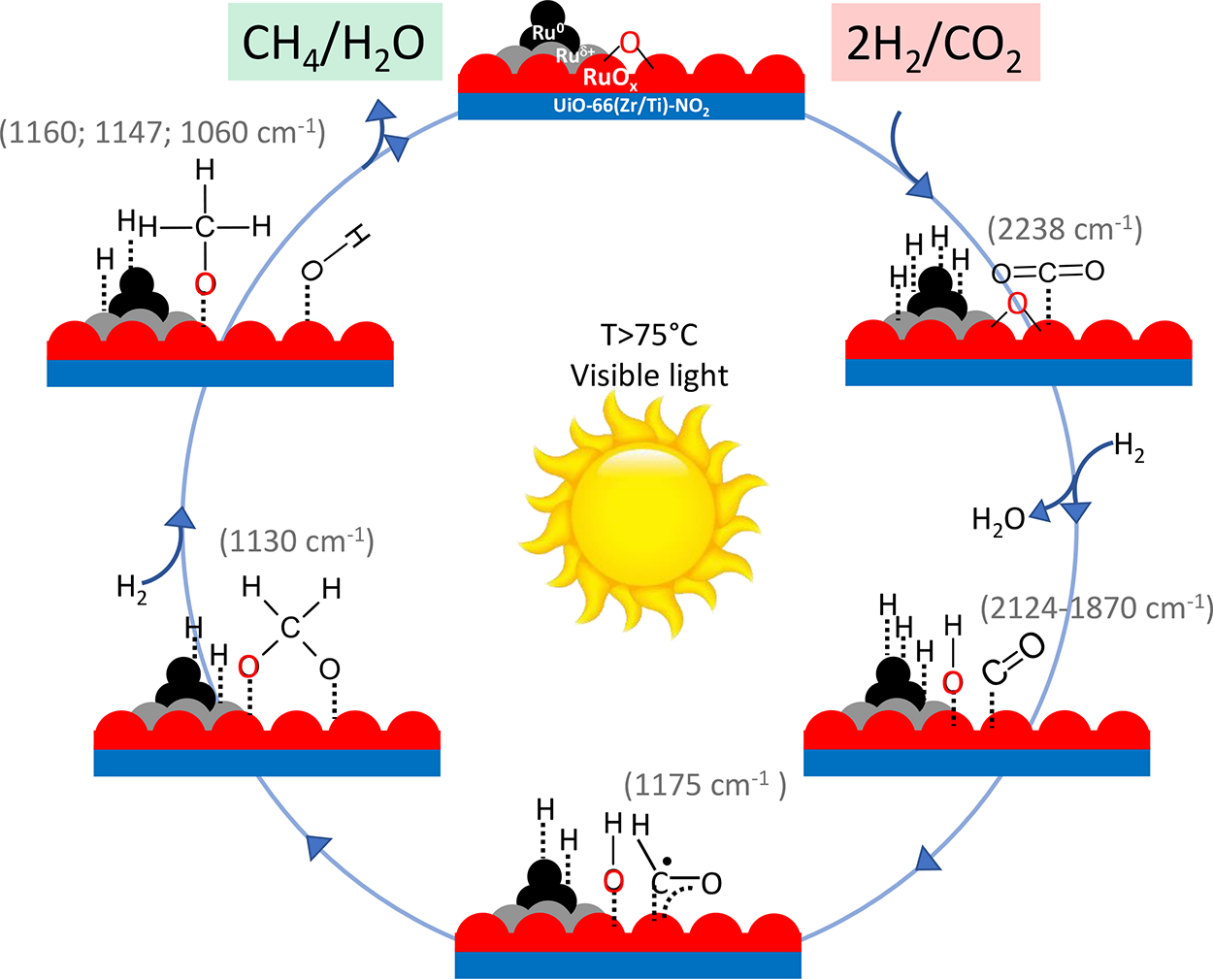
Proposed mechanism of the photoassisted CO_2_ methanation over RuO_*x*_@UiO-66(Zr/Ti)-NO_2_ based on the assignment of the characteristic FTIR bands of the various species.

Based on the spectral investigations mentioned above, an overall mechanism is proposed for the reaction unfolding as illustrated in Figure 14. Initially, CO_2_ is adsorbed on the surface, primarily on Ru species, accompanied by a hydride formation of reduced Ru. Subsequently, CO is generated as the primary intermediate of CO_2_ reduction, which exhibits strong surface adsorption. This is evident from the absence of CO as final product in the gas phase, as confirmed by FTIR and GC analyses (Figure 13b,c). As temperature increases, the photoassisted reduction of CO to formyl is promoted, followed by its conversion to dioxymethylene through interaction with surface oxygen. Then, the photoassisted reduction of dioxymethylene produces methoxy as the final intermediate species before generating methane and water as final products through further reduction. This mechanism emphasizes the dual role of RuO_*x*_ and reduced Ru in the production of methane from CO_2_ and H_2._

## 4 Conclusions

This study describes the development of multifunctional and photocatalytically active UiO-66 solids supported RuO_*x*_ NPs (2 ± 0.1 nm) for CO_2_ methanation at 200 °C under simulated concentrated sunlight irradiation. The photocatalytic activity of the samples followed the order UiO-66(Zr/Ti)-NO_2_ > UiO-66(Zr/Ti)-NH_2_ ∼ UiO-66(Zr)-NO_2_ > UiO-66(Zr)-NH_2_. In contrast to most reports involving UiO-66 photocatalysts based on the use of the 2-aminoterephthalate ligand, the present study highlights the importance of using 2-nitroterephthalate ligands to achieve high activity with Zr(IV) or mixed-metal Zr(IV)/Ti(IV) nodes within UiO-66-based materials and associated with the unique energy band level diagram of these solids. It should be noted that UiO-66(Zr/Ti)-NO_2_ is a reusable photocatalyst that exhibits record activity (5.03 mmol g^–1^ after 22 h; AQY at 350, 400, and 600 nm of 1.67, 0.25, and 0.01%, respectively) compared to previous analogous reports on MOF-based materials. Based on the results of several spectroscopic, electrochemical, and photocatalytic experiments, we consider that RuO_*x*_@ UiO-66(Zr/Ti)-NO_2_ operates in a dual photochemical and photothermal reaction pathway. The photocatalytic CO_2_ hydrogenation pathway was further investigated in flow condition using operando FTIR spectroscopy. The results are in very good agreement with those obtained under batch conditions. Based on the surface analysis and SSITKA experiment, a mechanism involving CO, formyl, dioxomethane, and methoxy, as intermediates, has been illustrated. In summary, we propose an innovative combination of nitro functionalized UiO-66 solids with mixed-metal Zr(IV)/Ti(IV) nodes and supported RuO_*x*_ NPs as cocatalyst to progress toward solar-driven photocatalytic CO_2_ methanation. The authors consider that this work will open new possibilities for the development of multifunctional MOFs as solar-driven photocatalysts for selective CO_2_ transformations.

## Supporting Information Available

Synthesis of the MOF-based materials; characterization techniques; photocatalytic activity; SEM and SEM-EDX mapping images; TEM images; XPS spectra; isothermal N_2_ adsorption graphs; TGA analyses; UV–vis diffuse reflectance spectra; valence band XPS spectra; PXRD; mass spectrum of ^17^CH_4_; femtosecond transient absorption spectra and decay traces; LFP spectra and decay traces; transient photocurrent plot; EPR spectra; direct spectra of chemisorbed CO_2_ and CO over RuO_*x*_@UiO-66(Zr/Ti)-NO_2_; photocatalytic CO_2_ methanation by H_2_ using MOF-based photocatalysts reported in the literature (Table S1); global fit analysis (Table S2); different vibrational modes of adsorbed CO over RuO_*x*_@UiO-66(Zr/Ti)-NO_2_ (Table S3); and vibrational modes of different adsorbed species over RuO_*x*_@UiO-66(Zr/Ti)-NO_2_ during the CO_2_ methanation reaction (Table S4) (PDF)
