# Supplementary material for: Solar Gas-Phase CO2 Hydrogenation by Multifunctional UiO-66 Photocatalysts
Source: ACS Catal. 2024 Apr 12;14(9):6470–87. doi: 10.1021/acscatal.4c00266 (PMC11075451; doi:10.1021/acscatal.4c00266)
Supplement: Supplementary file 1 — cs4c00266_si_001.pdf [file cs4c00266_si_001.pdf]

Supporting Information

Solar gas-phase CO_2 hydrogenation by multifunctional UiO-66 photocatalysts

Celia M. Rueda-Navarro,[1] Zahraa Abou Khalil,[2] Arianna Melillo,[1] Belén Ferrer,[1] Raúl Montero,[3] Asier Longarte,[4] Marco Daturi, [2] Ignacio Vayá,[1,5] Mohamad El-Roz,[2] Virginia Martínez-Martínez,[4] Herme G. Baldoví,[1,*] Sergio Navalón[1,*]

[1] Departamento de Química, Universitat Politècnica de València, Camino de Vera s/n, Valencia 46022, Spain

[2] Normandie Université, ENSICAEN, UNICAEN, CNRS, Laboratoire Catalyse et Spectrochimie, 14000 Caen, France.

[3] SGIker Laser Facility, UPV/EHU, Sarriena, s/n, 48940 Leioa, Spain

[4] Facultad de Ciencia y Tecnología, Departamento de Química Física, Universidad del País Vasco (UPV/EHU), Apart. 644, 48080 Bilbao, Spain.

[5] Instituto de Tecnología Química (UPV-CSIC), Universitat Politècnica de València, C/Avenida de los Naranjos s/n, Valencia 46022, Spain

Emails: hergarba@cam.upv.es (H.G.); sernaol@doctor.upv.es (S.N)

S1. Synthesis of the MOF-based materials

Synthesis of UiO-66(Zr)-NH_2. The solid was prepared using a solvothermal method previously reported [1-3]. Briefly, $ZrCl_4$ (0.862 g, 3.699 mmol), amino-terephthalic acid (0.677 g, 3.701 mmol) and Mili-Q H_2O (0.200 mL, 11.10 mmol) were mixed with dimethylformamide (DMF) (100 mL, 1291 mmol) in 250 ml volumetric flask. The system was heated at 70 °C and stirred during the preparation to ensure complete dissolution of the reagents. Once all reagents were dissolved, the stirring magnet was removed from the system and the flask was closed. Then, it was placed in a pre-heated oven at 120 °C for 72 hours. The resulting precipitate was recovered by filtration. Then, it was washed three times with DMF and three more with methanol. It was further washed in a Soxhlet for 4 h using methanol as the solvent. And finally, the powder was dried under vacuum at 150 °C overnight.

Synthesis of UiO-66(Zr)-NO_2 [1-3]. Briefly, $ZrCl_4$ (0.862 g, 3.699 mmol), nitro-terephthalic acid (0.790 g, 3.701 mmol) and Mili-Q H_2O (0.200 mL, 11.10 mmol) were mixed with DMF (100 mL, 1291 mmol) in 250 ml volumetric flask. The system was heated at 70 °C while stirring to ensure the complete dissolution of the reagents. Once all reagents were well dissolved, the flask was placed in a pre-heated oven at 120 °C for 72 hours after removing the stirring magnet and closing the system. The resulting precipitate was recovered by filtration. Then, it was washed three times with DMF and subsequently, three more with methanol. It was further washed in a Soxhlet for 4 h using methanol as the solvent. Finally, the powder was recovered drying it under vacuum at 150 °C overnight.

UiO-66(Zr/Ti)-NH_2 and UiO-66(Zr/Ti)-NO_2. Both solids were prepared following a post-synthetic method [4, 5] using $TiCl_4(THF)_2$ as Ti source under inert atmosphere. 0.45 mmol of previously synthesized UiO-66(Zr)-NH_2 or UiO-66(Zr)-NO_2, respectively were mixed with

0.135 mmol $TiCl_4(THF)_2$ and suspended in 2.5 mL anhydrous DMF. The mixture was incubated through stirring and under argon atmosphere for 4 days at 120 °C. After cation exchange, the corresponding UiO-66(Zr/Ti) solids were recovered by filtration and sequentially washed with DMF and MeOH. Finally, the photocatalysts were dried at 100 °C for 24 h.

Deposition of Ru nanoparticles on the mentioned solids. Ru NPs were deposited in the as-prepared MOFs using the so-called photodeposition method [6]. Succinctly speaking, the material (50 mg) was introduced in a quartz tube and dispersed in a mixture of Milli-Q water (8 mL) and methanol (13 mL). Subsequently, the corresponding amount of potassium perruthenate (1%wt of Ru) previously dissolved in water (1 mL) was added to the dispersion. Then, the system was purged with Ar for 30 min and immediately irradiated using a UV-vis light lamp for 4 h. Finally, the resulting solid was filtered and washed several times with Milli-Q water, dried in an oven at 100 ºC overnight and then, left under vacuum at 150 ºC, 24 h.

S2. Characterization techniques

Powder X-ray diffraction (PXRD) data was recorded on a Philips XPert diffractometer equipped with a graphite monochromator (40 kV and 45 mA) employing Ni filtered CuKα radiation (0.15418 nm).

UV-Vis diffuse reflectance measurements of the solid samples were performed on a Varian spectrometer model Cary 5000.

X-ray photoelectron spectra (XPS) were acquired on a SPECS spectrometer equipped with an MCD-9 detector using a monochromatic Al X-ray source (K_α= 1486.6 eV). The C 1s peak at 284.4 eV was employed as reference binding energy. CASA software has been

employed for spectra deconvolution. The valence band maximum of the photocatalyst respect to the Fermi level (E^f_υ) was also determined by XPS.[6] The valence band position vs. NHE (E_υ^{NHE}) was calculated from the equation: $E_\upsilon^{NHE} = E_\upsilon^f + \phi_{sp} + E_0^{SHE}$, where ϕ_{sp} is the work function of the spectrometer used for the measurements (4.244 eV) and E_0^{SHE} is the energy of the SHE with respect to vacuum level of the electron with the value of −4.44 eV. The conduction band energy minimum of the photocatalyst was determined by the difference between the E_υ^{NHE} value and the optical band gap.

Scanning electron microscopy (SEM) measurements were carried out using a scanning electron microscope (SEM, Zeiss instrument, AURIGA Compact) equipped with an energy-dispersive X-ray (EDX) detector.

Scanning transmission electron microscopy images in dark field mode (DF-STEM) were collected on a JEOL JEM2100F instrument (200 kW).

Isothermal N_2 adsorption measurements were performed at 77 K using a Micromeritics station (ASAP 2010).

Thermogravimetric analyses were carried out on a TGA/SDTA851e METTLER TOLEDO station.

Photoelectrochemical analysis including electrochemical impedance spectroscopy (EIS) and photocurrent measurements were performed using a Gamry Instruments potentiostat (model Interface 5000E). A standard three-electrode configuration was used in a home-made quartz electrochemical cell with a platinum wire as counter electrode and a saturated Ag/AgCl electrode as the reference. The working electrode was prepared as follows. Firstly, a paste of each material was obtained by mixing 20 mg of photocatalyst with

0.2 mL of terpineol and 0.5 mL of acetone. Secondly, the mixture was left stirring until complete dispersion. Afterwards, the mixture was left under stirring and incubation at 90 ºC overnight. After letting cool down, 25 µL of each sample was spread onto an area of 1.0 x 1.0 cm^2 using doctor blade technique though a conductive carbon Toray paper with dimensions of 2.0 x 1.0 cm^2. Finally, the electrode was thermally treated at 150 °C for 1 hour.

The photocurrent generated by the electrodes was measured by chopped Linear sweep voltammetry (LSV) with an electrolyte of 0.1 M Tetrabutylammonium hexafluorophosphate (NBu_4PF_6) acetonitrile solution. The applied potential was 0.9 V. And for some LSV measurements, MeOH (0.3 mL) was added as a hole scavenger.

Electrochemical impedance spectroscopy (EIS) was carried out with frequencies ranging from 0.1 Hz to 100 kHz at 0.2 V. Prior to the measurements, the electrolyte solutions were purged with argon for 10 minutes. UV-Vis irradiation of the working electrodes was carried out with a spot light Hamamatzu Xe lamp (Lightnincure LC8 model, 800–200 nm, 1000 W/m^2, fiber optic light guide with a spot size of 0.5 cm).

Femtosecond transient absorption spectroscopy. Ultrashort laser pulses are generated in an oscillator-regenerative amplifier laser system (Coherent, Mantis-Legend) providing of 35 fs pulses at 800nm. UV pump pulses (267 nm) are produced as the third harmonic of the fundamental beam. The white light continuum probe is produced by focusing (f=100 mm) a small fraction of the amplifier output on a 2 mm thick CaF2 window. Pump probe delay is achieved with a translation stage (Thorlabs, DDS220) that allows a maximum range of 2 ns. Transient absorbance is measured with a fiber-coupled spectrometer (Avantes, Avaspec) as a function of the pump probe delay. The spot radii of the pump and probe beams are 0.35 mm and 0.1 mm, respectively. Pump pulse energies were varied in the range 2.5-

5µJ. The samples, prepared in a 2 mm quartz cuvette, were continuously stirred and scanned across the focal plane to avoid thermal effects on the sample.

The decay kinetics was analyzed by means of a two exponential decay including an offset to account for the longer-lived contributions. Global decay analysis was performed including decays at 10 different wavelengths in the range 550-725 nm. Average lifetimes were determined as the average of the decay constants weighted with the pre-exponential parameters resulting from the fitting averaged from all measured wavelengths.

Laser Flash Photolysis. Measurements were performed using a pulsed Nd:YAG L52137 V LOTIS TII at λ_{exc} = 266 nm. The single pulses were *ca*. 10 ns duration, and the energy was ~12 mJ/pulse. The LFP system consisted of the pulsed laser, a 77250 Oriel monochromator and an oscilloscope DP04054 Tektronix. The output signal from the oscilloscope was transferred to a personal computer. Absorbances of all solutions were adjusted at ~0.30 at the excitation wavelength in acetonitrile (HPLC grade). Measurements were done using 10 × 10 mm^2 quartz cuvettes at room temperature in argon atmosphere (25 min Ar bubbling). Control experiments indicated that the degree of decomposition of the samples after photolysis was lower than 2%. The LFP decay traces were fitted using a multi-exponential function following the Levenberg-Marquardt iteration algorithm (equation 1):

$$F(t,\lambda) = \sum_{i=1}^{n} a_i(\lambda) e^{\left(-\frac{t}{\tau_i}\right)} + y_0 \qquad (1)$$

with n = 2 or 3

Fluorescence spectroscopy. Steady-state fluorescence measurements (λ_{exc} = 266 nm) were performed on an Edinburgh FS5 spectrofluorometer, provided with a monochromator in the wavelength range of 200-900 nm. The absorbance of the samples was identical (*ca*. 0.1) at

the excitation wavelength. Time-resolved fluorescence measurements were performed using an EasyLife X system containing a sample compartment composed of an automated peltier cuvette holder to control the temperature at 24 ºC, a pulsed LED excitation source and a lifetime detector. The employed LED excitation source was 265 nm, with emission filter of WG305. The fluorescence lifetimes (τ_F) were obtained upon fitting the decay traces by a non-linear fitting/deconvolution procedure $F(t) = \Sigma a_i \cdot exp(-t/\tau_i)$ using a one- or two-exponential function, depending on the investigated system. All spectroscopic measurements (UV, fluorescence and LFP) were done using 10 × 10 mm^2 quartz cuvettes at room temperature.

The photoluminescence measurements were conducted using commercially available CdSe-ZnS core-shell quantum dots. A spectrofluorimeter was utilized to analyze the photoluminescence spectrum of either CdSe/ZnS core-shell QDs or RuO_xUiO-66(Zr/Ti)-NO_2-CdSe/ZnS upon excitation at 450 nm. To achieve this, 1 mg of CdSe/ZnS QDs was dissolved in 3 mL of CH_3CN for each case. Adding 2 mg of RuO_xUiO-66(Zr/Ti)-NO_2 for the analysis of the MOF. 40 μL of both solutions were applied dropwise onto 2 sets of quartz supports, where the solvent was evaporated using a heating plate at 90 ºC during 5 min. One of the samples for each material supported on a quartz holder were then heated for 5 min on a hot plate to the appropriate temperature (from 200 to 280 ºC) and then measured using the spectrofluorimeter. The other two samples were irradiated during 13 minutes at the reference temperature of the photocatalysis experiments (T= 200 ºC) under different light intensities (385, 220, 140, 85 mW/cm^2) with a xenon lamp containing a visible light filter.

ScanTemp410 Infrared-thermometer was used to monitor the temperature for PL experiments. The range to measure the temperature is within -3°C to 500°C.

S3. Photocatalytic activity

Photocatalytic experiments under batch reaction conditions were carried out using a quartz reactor (50 mL) equipped with a heating mantle and thermocouple. The UiO-66 based photocatalyst is placed on the bottom of the reactor and, then, the system purged with H_2 for 15 min and then CO_2 was introduced to obtain a H_2:CO_2 ratio 4:1 and 1.5 bar pressure. Irradiations were performed using a Hg-Xe lamp (150 W, Hamamatsu ref. L8253; Hamamatsu spot light source L9566-04 and light guide A10014-50–0110) with or without an AM 1.5G type filter (Lasing ref. 81094) to obtain simulated sunlight irradiation. The influence of radiation intensity on the photocatalytic activity was carried out by using transmittance filters (Newport, ref. FSQ-OD30, FSQ-OD15 or FSQ-QD05). The course of the reaction was followed by analyzing gas phase reaction aliquots in an Agilent 490 MicroGC equipped with a thermal conductivity detector. A MolSieve 5A column is used to analyse H_2, O_2, N_2, and CO while a Pore Plot Q column analyse to analyse the CO_2, CH_4, and short-chain hydrocarbons (ethane, ethylene, propane, propylene, and butane). Quantification was by calibration plots with commercially available gas mixtures.

Operando FTIR photocatalytic tests

Photocatalytic tests were performed using an *operando* FTIR setup, which allows to monitor through real time both the gas phase products and the surface of the catalyst during the reaction. For that, a "Sandwich" IR cell reactor was used and the photocatalytic experiments were conducted under the following conditions: 4:1 molar ratio of H_2 to CO_2 with a total flow rate of 10 cm^3 min^{-1}; visible light irradiation (Xe lamp LC8Hamamatsu with cut-UV filter λ >390 nm) irradiance= 71 mW/cm^2); and 20 mg of photocatalyst. The sample was preactivated under H_2 at 200 °C with a heating flow rate of 5 °C. min^{-1}. Then the reaction was conducted at different temperaturas in dark and under irradation The catalyst surface as

well as the composition of the output gas from the reactor were simultaneously analyzed by an IR spectrometer(ThermoNico-letNEXUS670FTIR) equipped with a mercury cadmium telluride (MCT) detector with a spectral resolution of 4 $cm^{-1}$and accumulating 64 scans. Mass spectrometry (MS, Quadru-pole Pfeiffer Omnistar GSD301) was also used to monitor the gas composition during the reaction. The produced gases were also confirmed by online gas chromatography (Compact-GC) with a Rtx-1-5u (30m - 0.32mm) capillary column equipped with a flame ionization detector (FID) and using commercially available samples (methane and ethane).

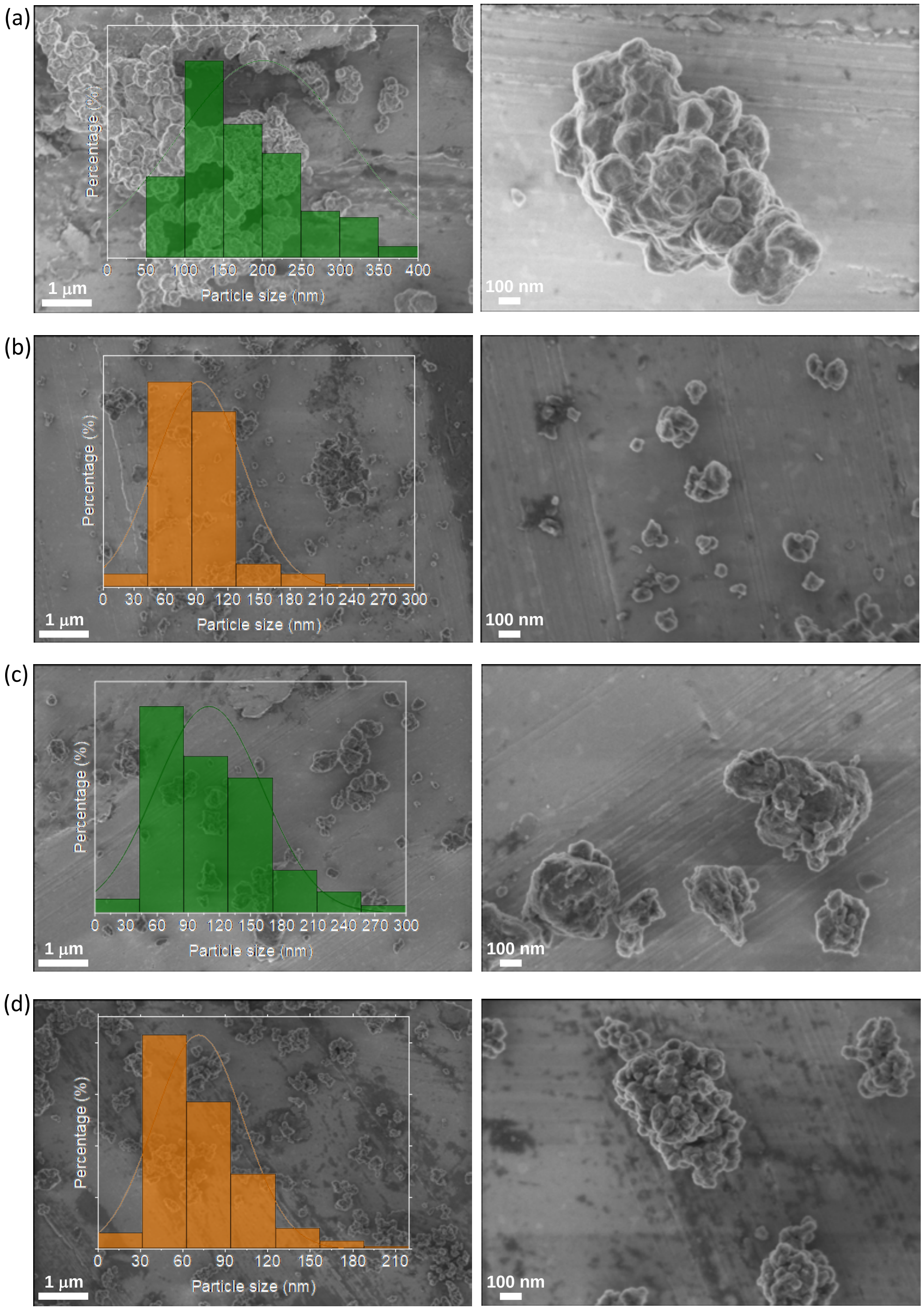

Figure S1. SEM images of (a) UiO-66(Zr)-NH_2, (b) UiO-66(Zr)-NO_2, (c) UiO-66(Zr/Ti)-NH_2, (d) UiO-66(Zr/Ti)-NO_2. Legend: MOF average particle size and standard deviation of UiO-66(Zr)-NH_2 (200 ± 111 nm), UiO-66(Zr/Ti)-NH_2 (110 ± 51 nm), UiO-66(Zr)-NO_2 (92 ± 42 nm), UiO-66(Zr/Ti)-NO_2 (71 ± 31 nm).

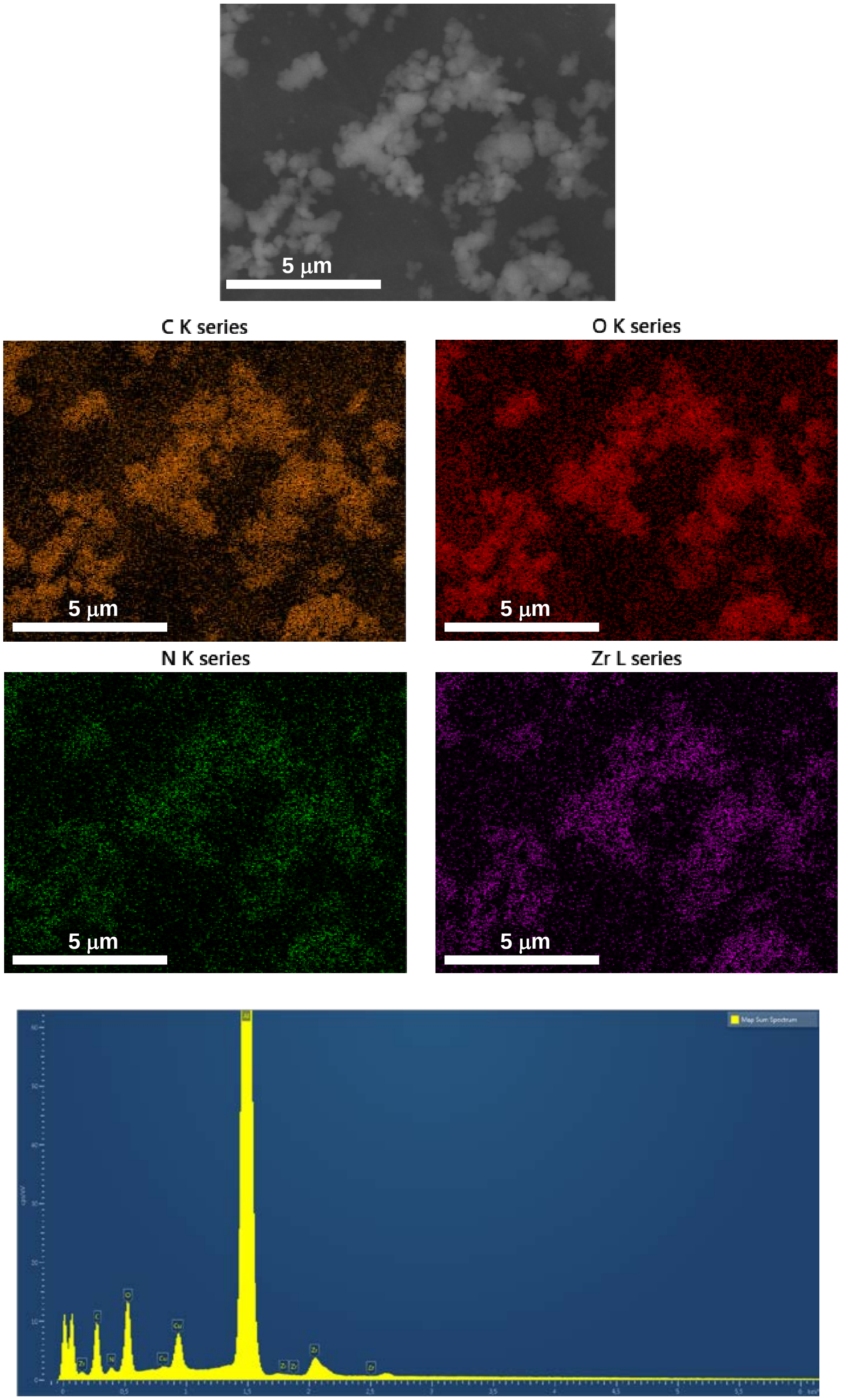

Figure S2. SEM-EDX of UiO-66(Zr)-NH_2.

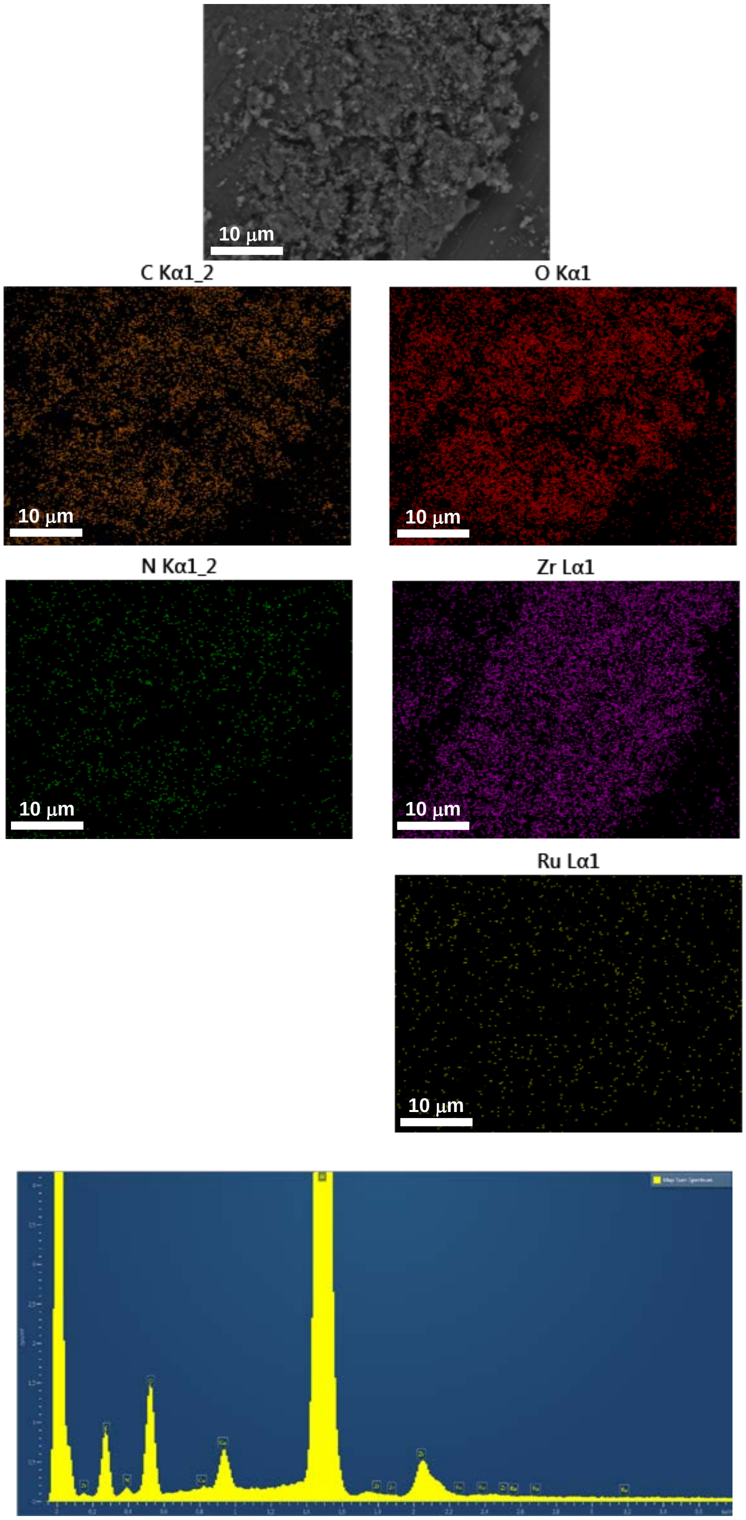

Figure S3. SEM-EDX of Ru@UiO-66(Zr)-NH_2.

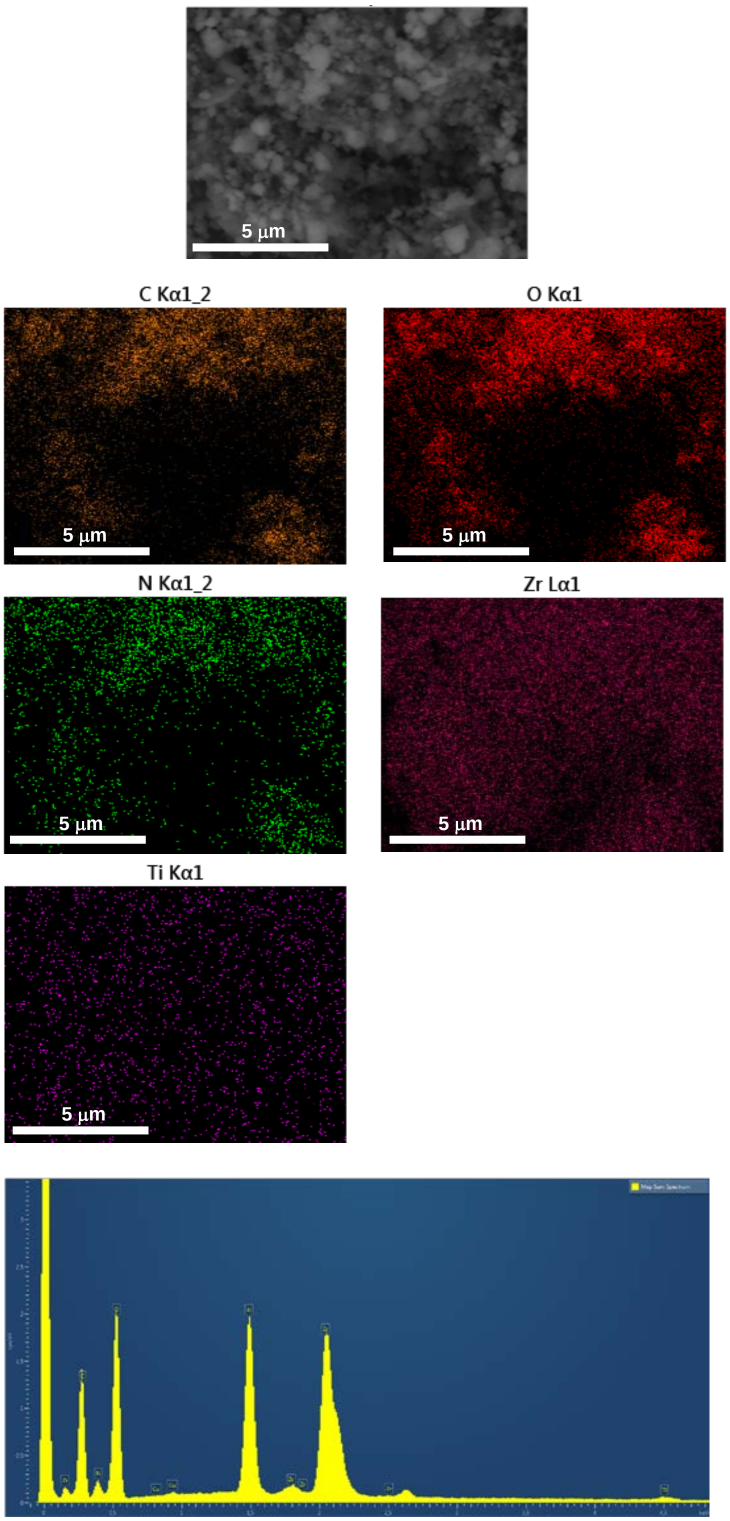

Figure S4. SEM-EDX of UiO-66(Zr/Ti)-NH_2.

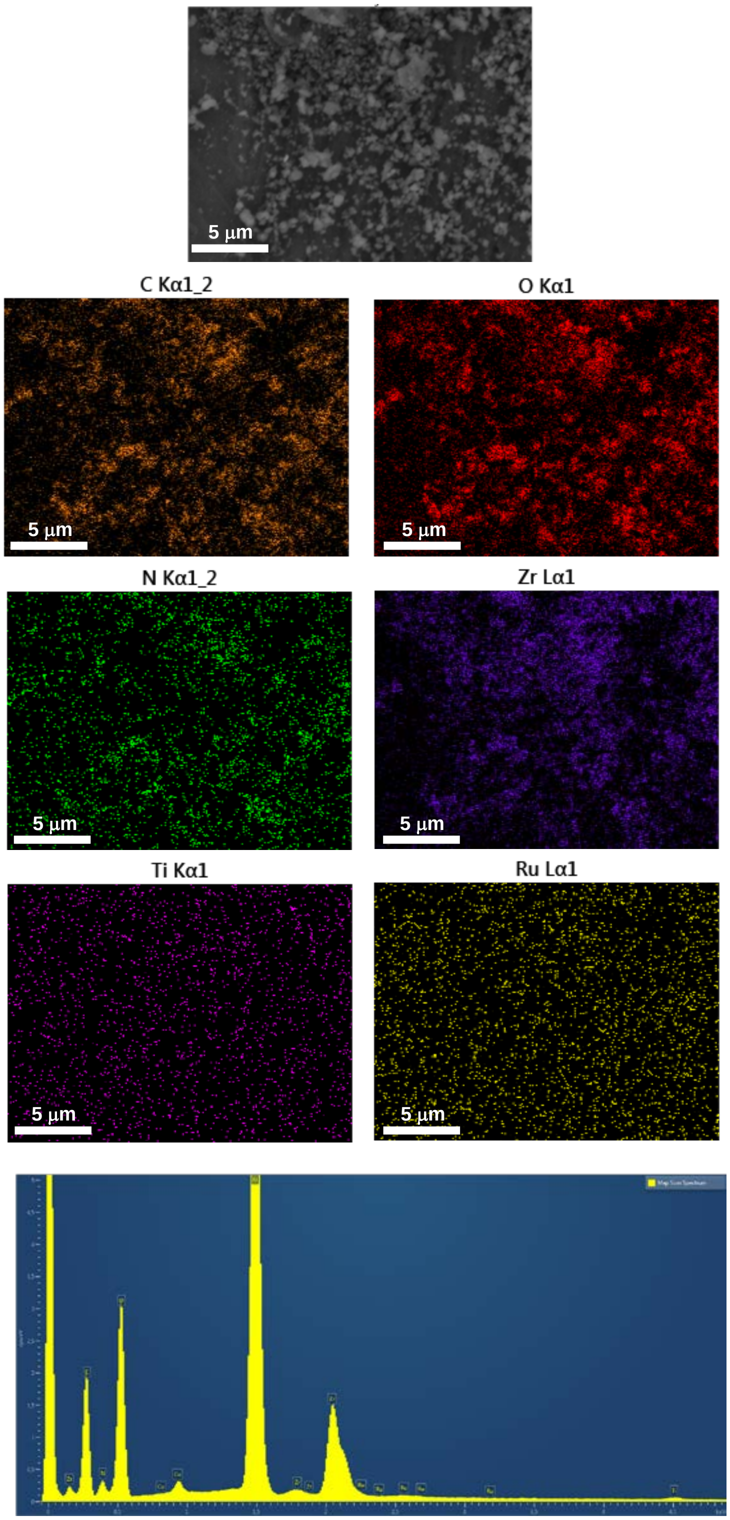

Figure S5. SEM-EDX of Ru@UiO-66(Zr/Ti)-NH_2.

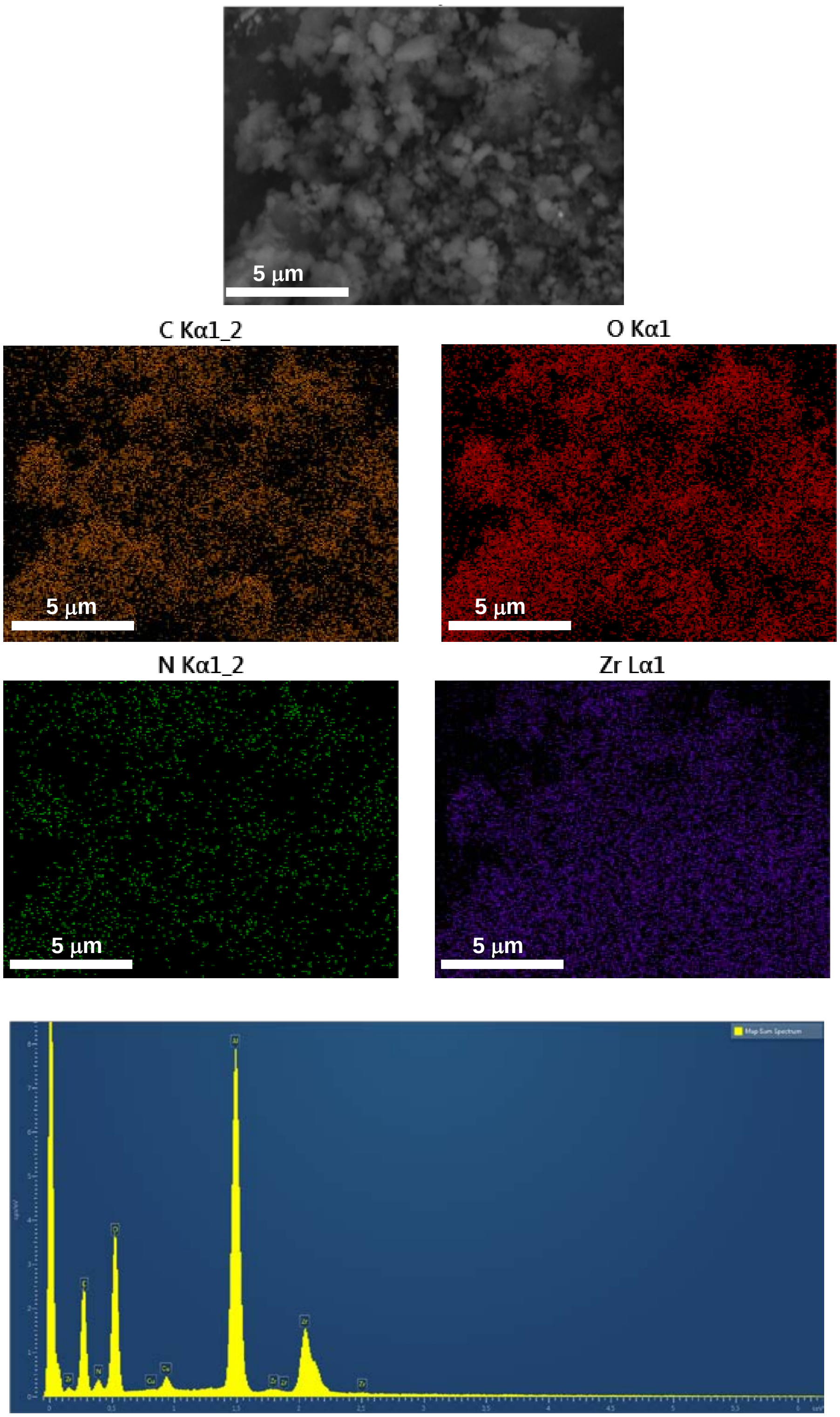

Figure S6. SEM-EDX of UiO-66(Zr)-NO_2.

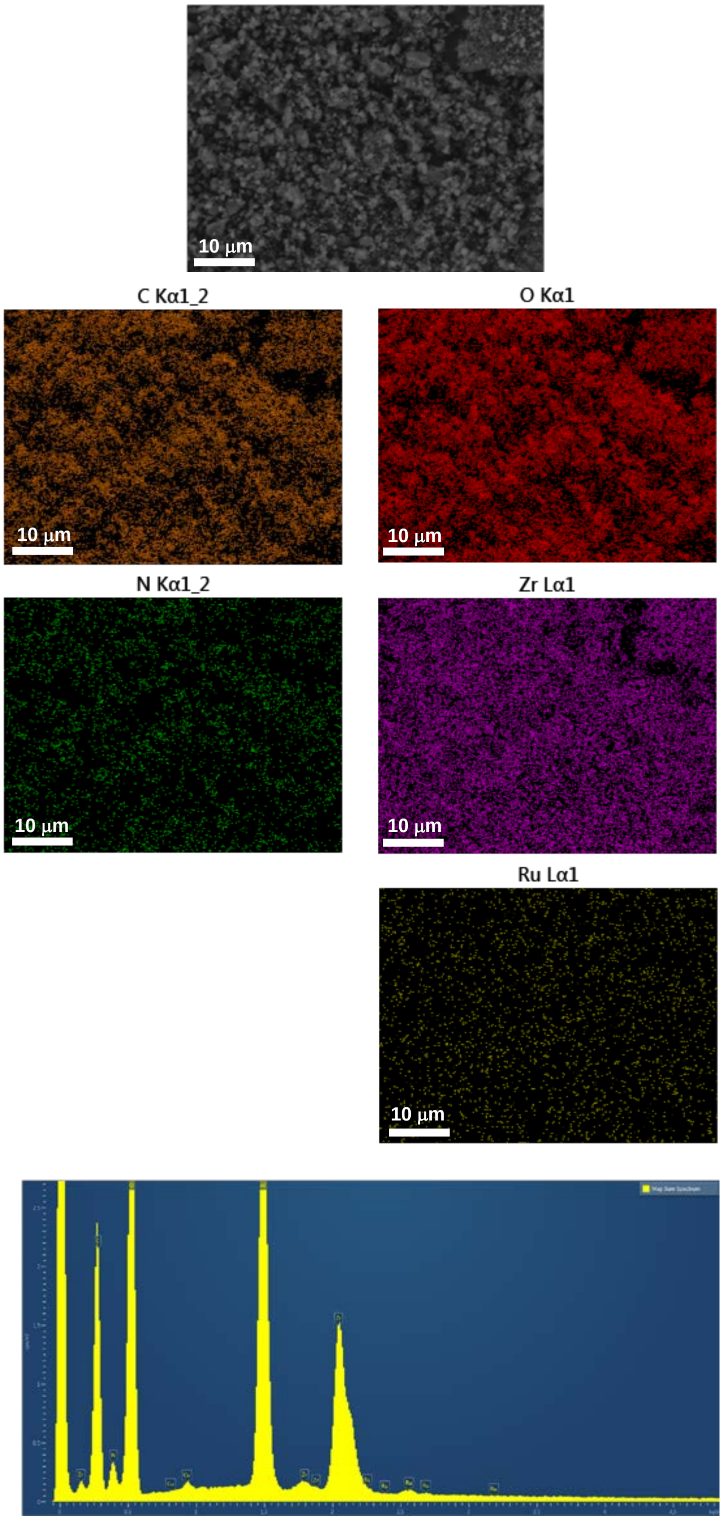

Figure S7. SEM-EDX of RuO_x@UiO-66(Zr)-NO_2.

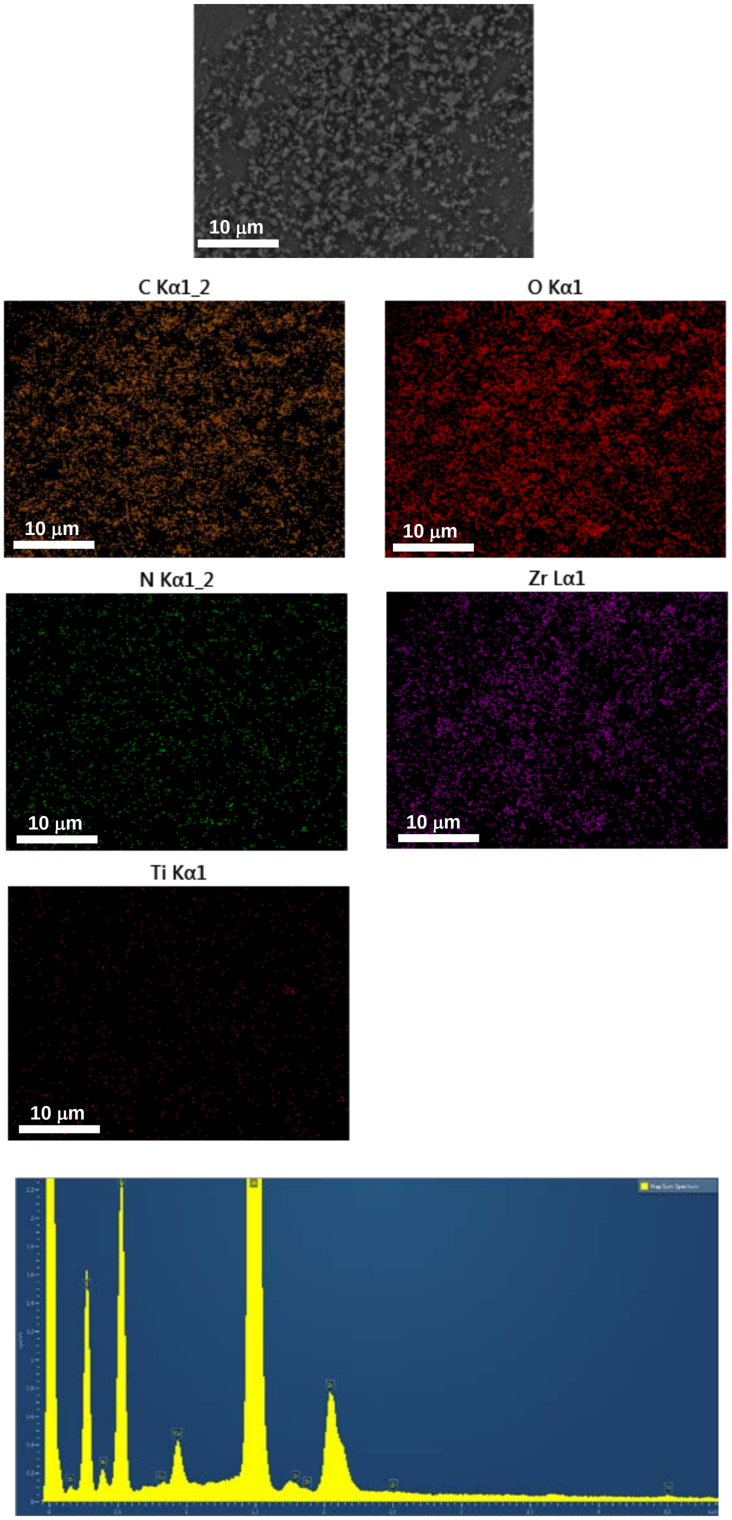

Figure S8. SEM-EDX of UiO-66(Zr/Ti)-NO_2.

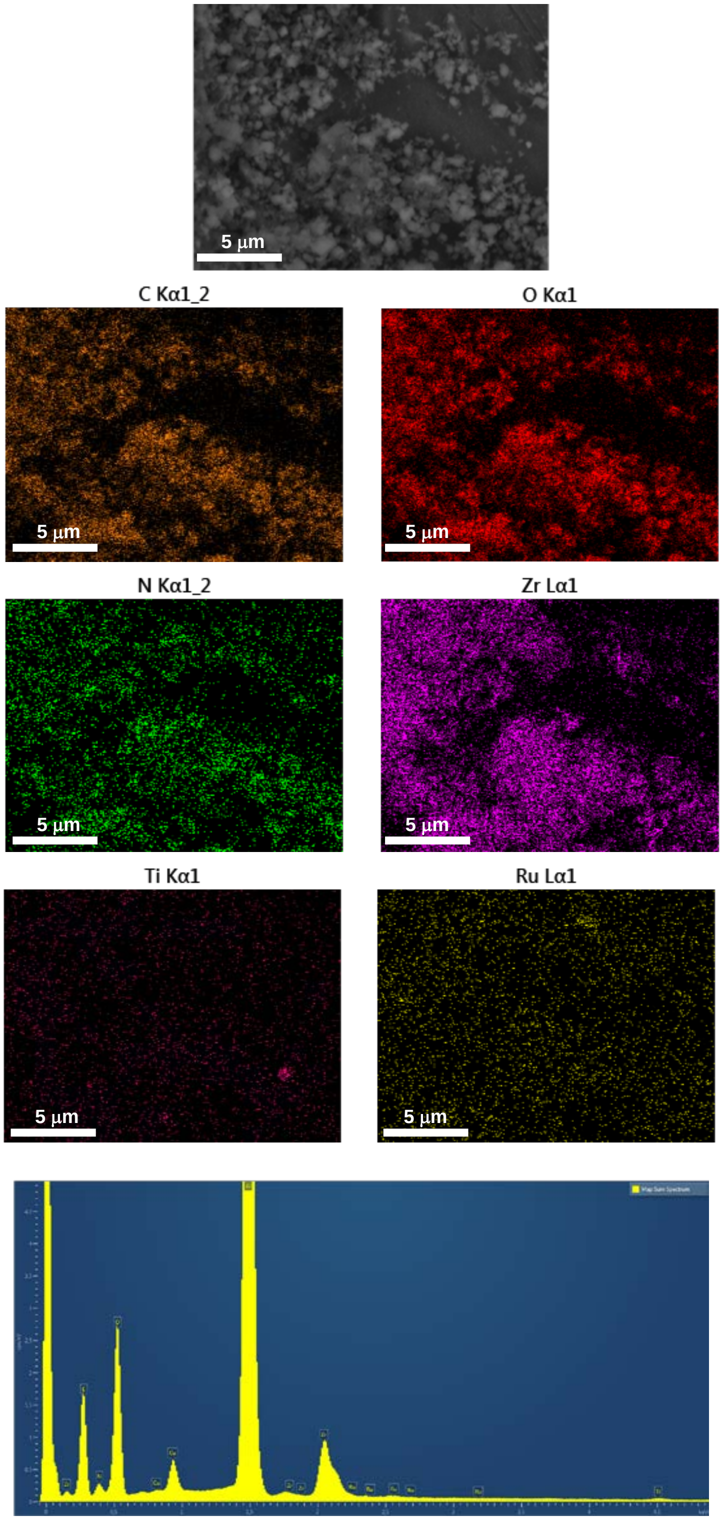

Figure S9. SEM-EDX of RuO_x@UiO-66(Zr/Ti)-NO_2.

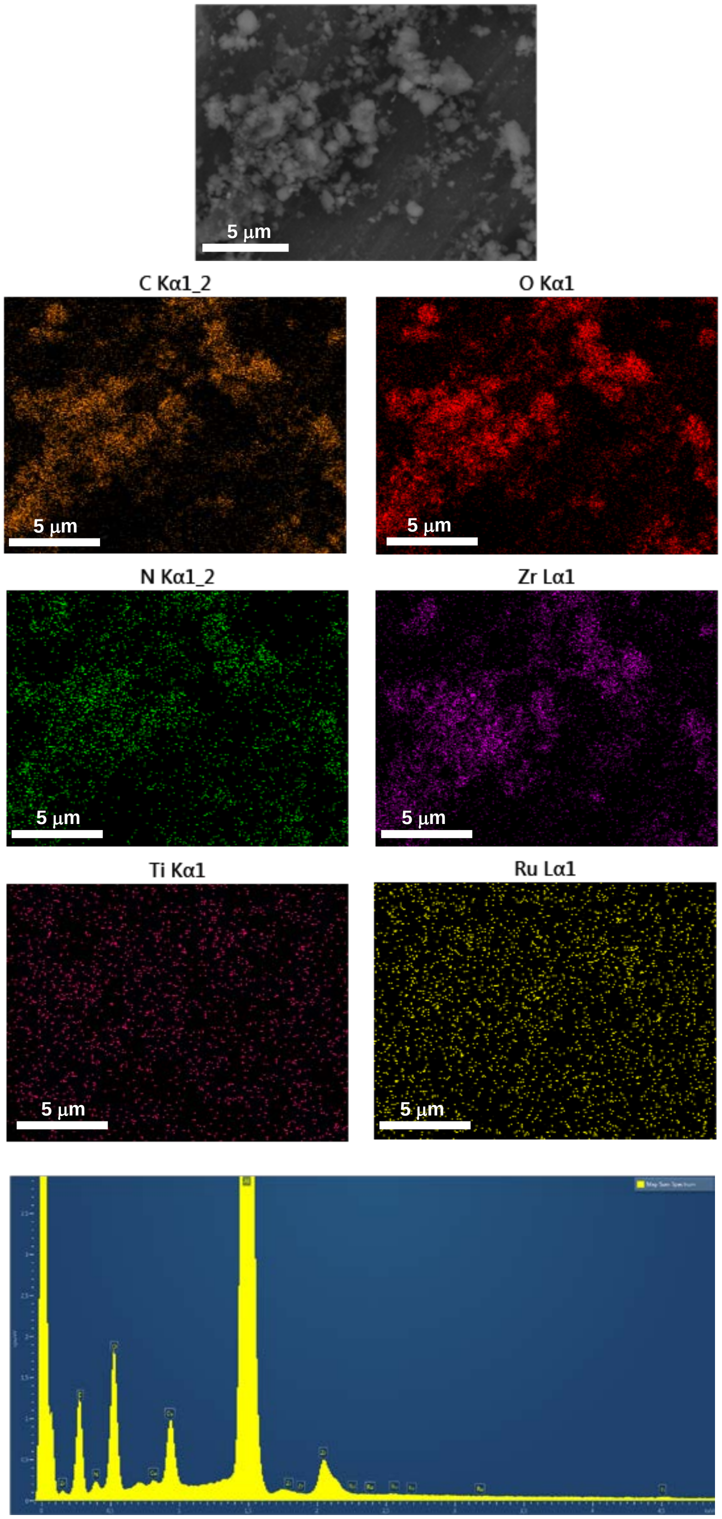

Figure S10. SEM-EDX of used RuO_x@UiO-66(Zr/Ti)-NO_2.

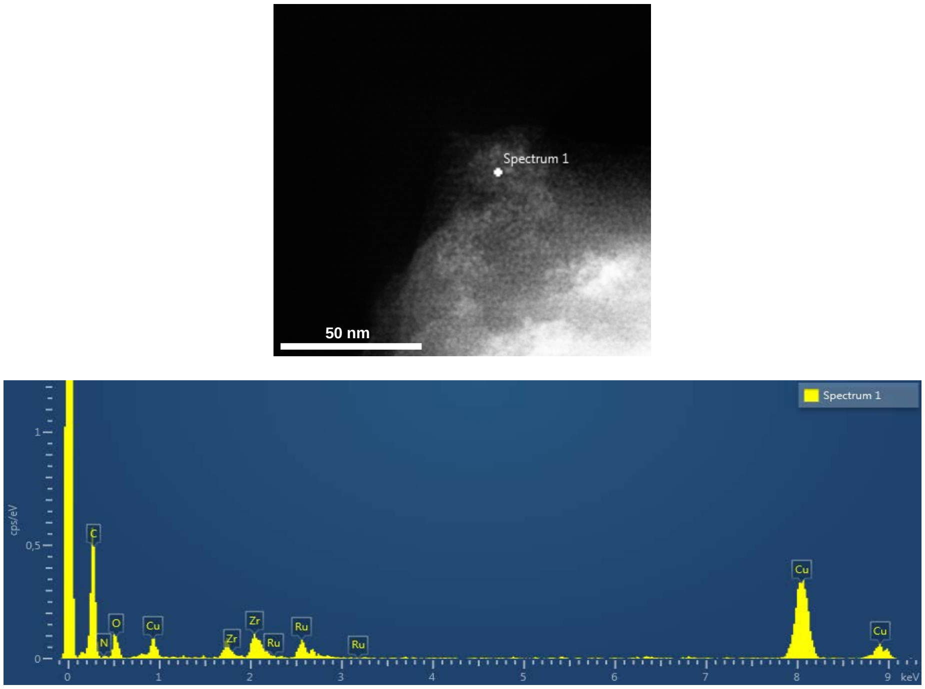

Figure S11. EDX image of RuO_x@UiO-66(Zr)-NH_2.

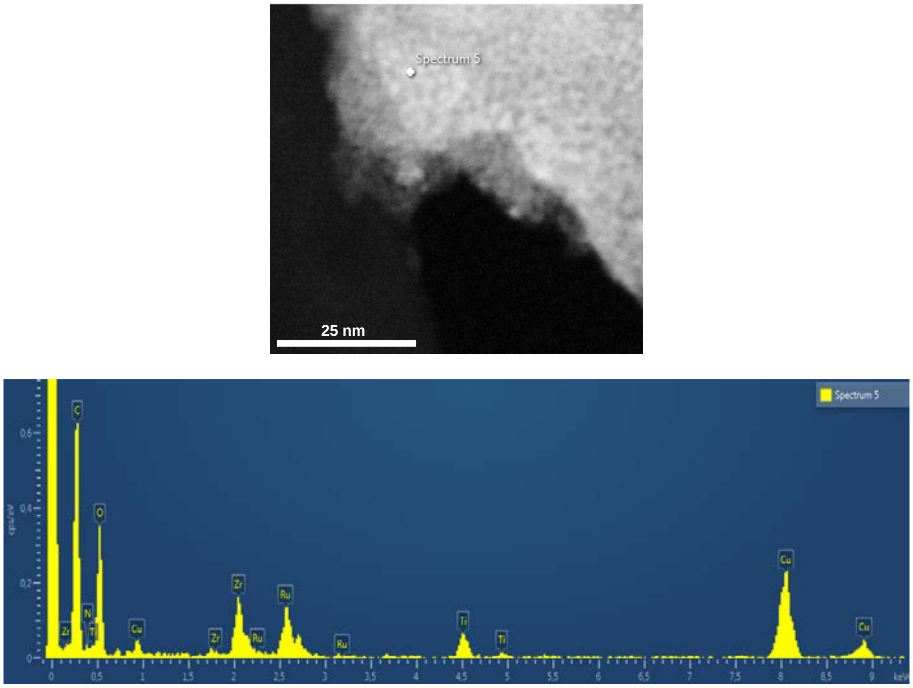

Figure S12. EDX image of RuO_x@UiO-66(Zr/Ti)-NH_2.

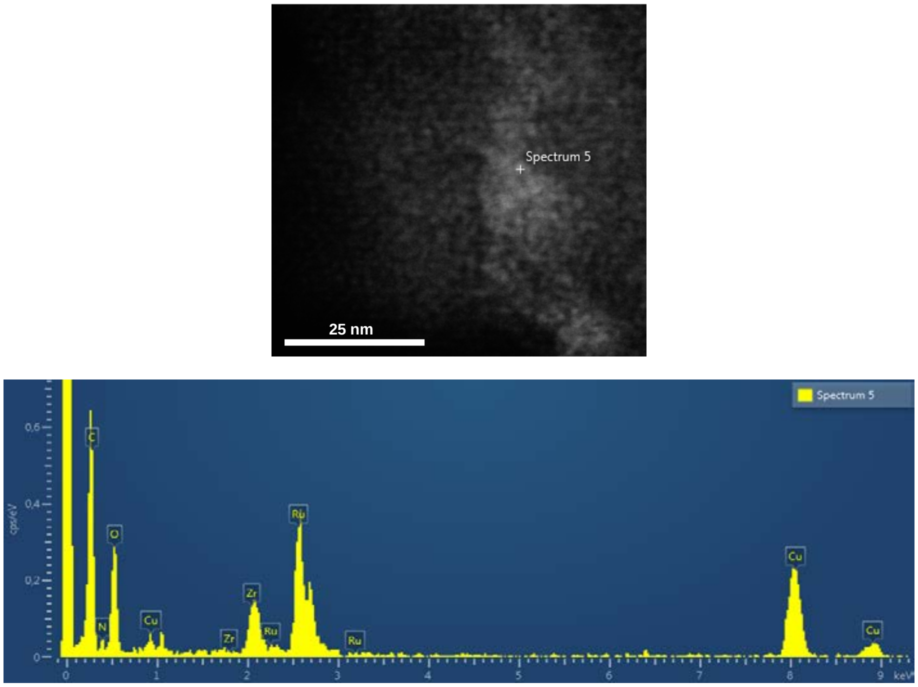

Figure S13. EDX image of RuO_x@UiO-66(Zr)-NO_2.

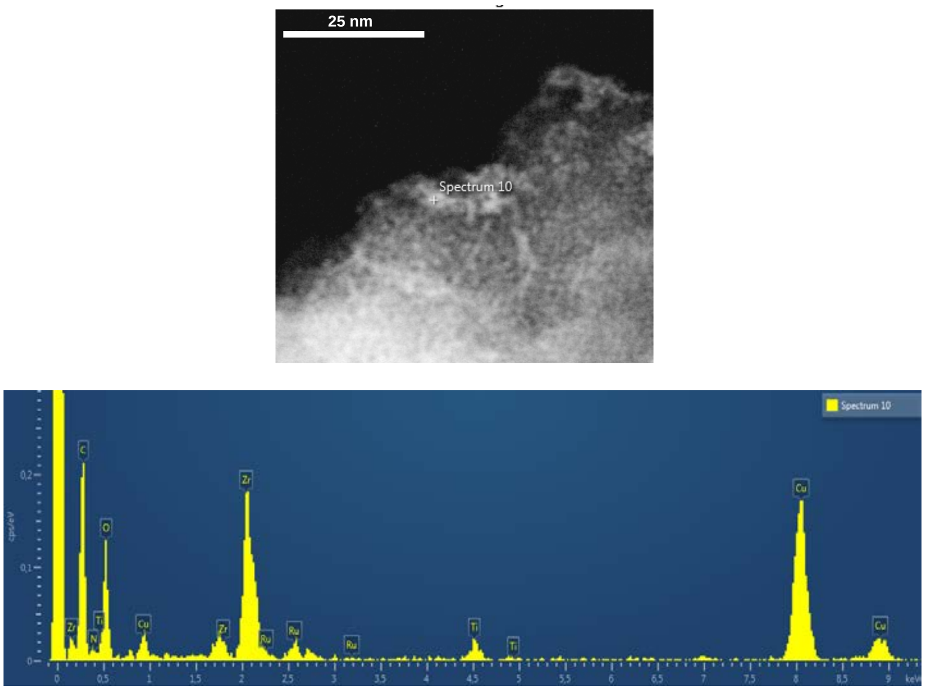

Figure S14. EDX image of RuO_xUiO-66(Zr/Ti)-NO_2.

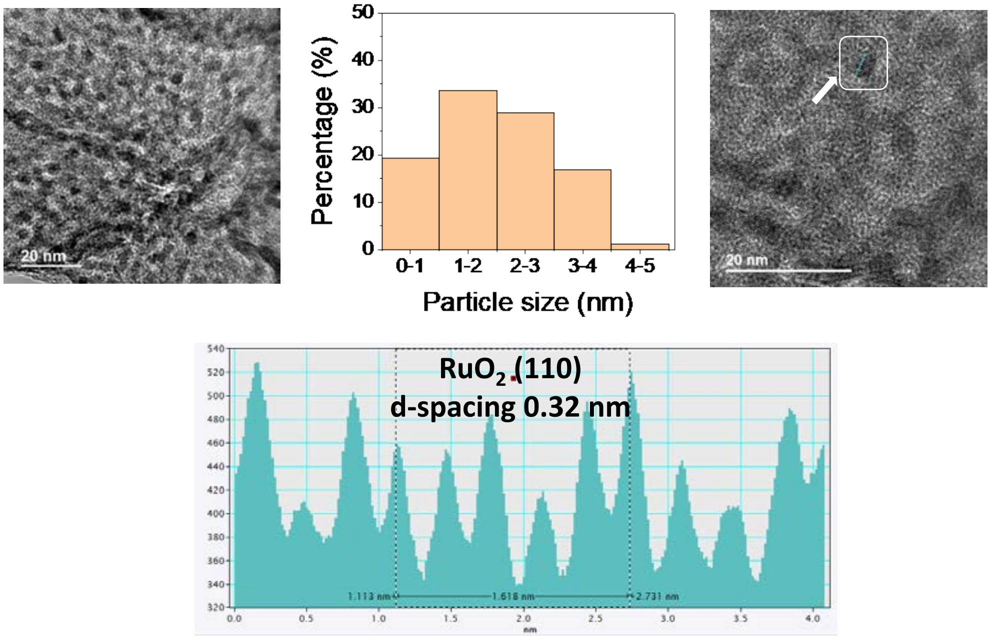

Figure S15. TEM image of RuO_xUiO-66(Zr)-NH_2 to show the selected region to measure the interplane distance. Average particle size and standard deviation of 2.17 ± 1.04 nm.

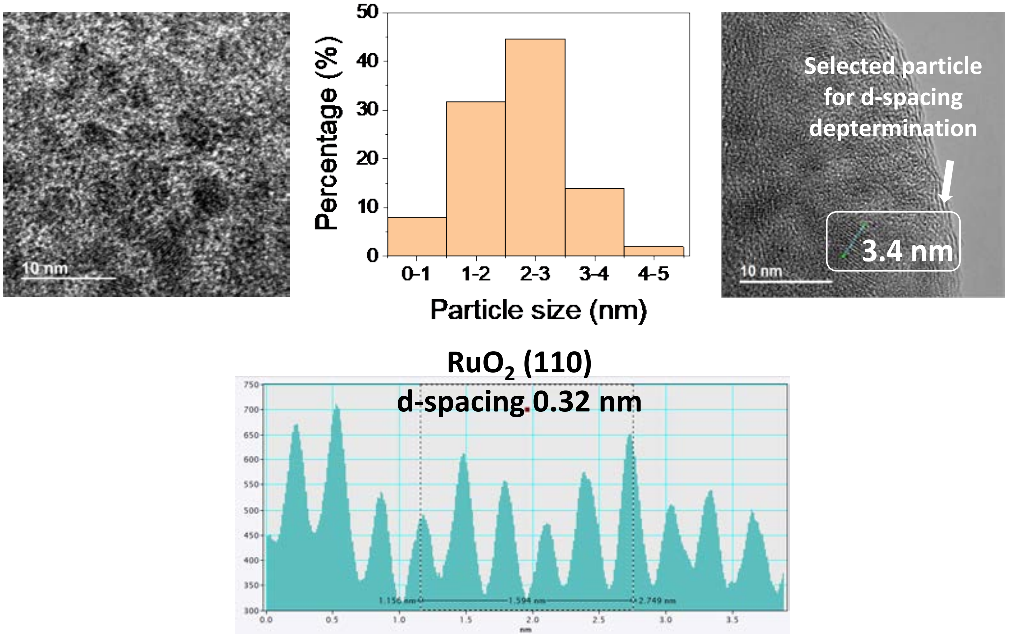

Figure S16. TEM image of RuO_xUiO-66(Zr)-NO_2 to show the selected region to measure the interplane distance. Average particle size and standard deviation of 2.16 ± 0.87 nm.

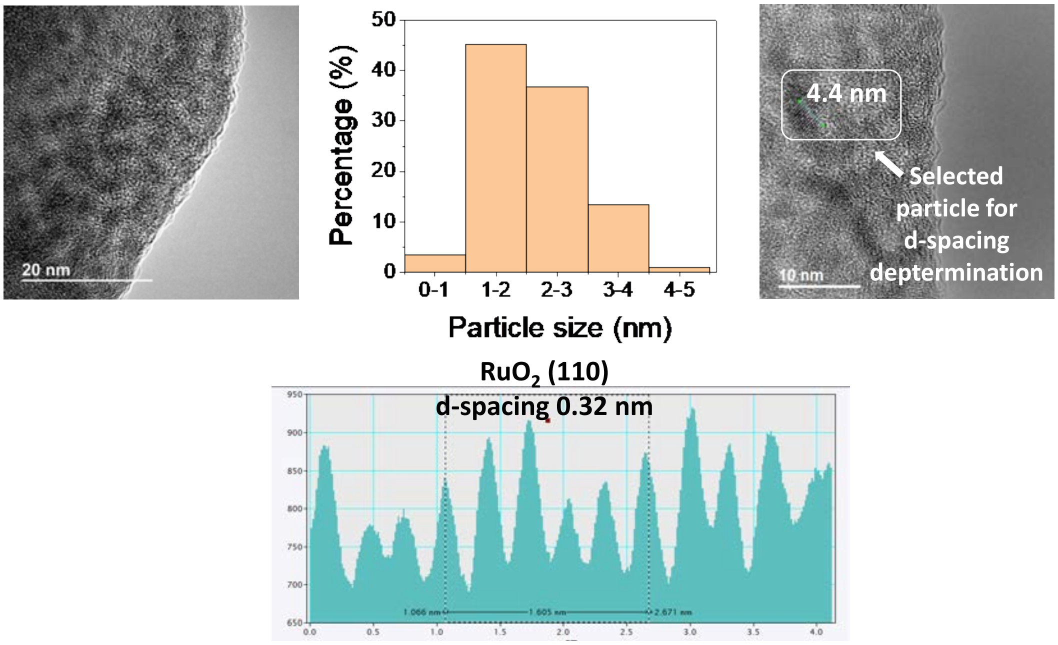

Figure S17. TEM image of $RuO_xUiO\text{-}66(Zr/Ti)\text{-}NH_2$ to show the selected region to measure the interplane distance. Average particle size and standard deviation of 2.15 ± 0.71 nm.

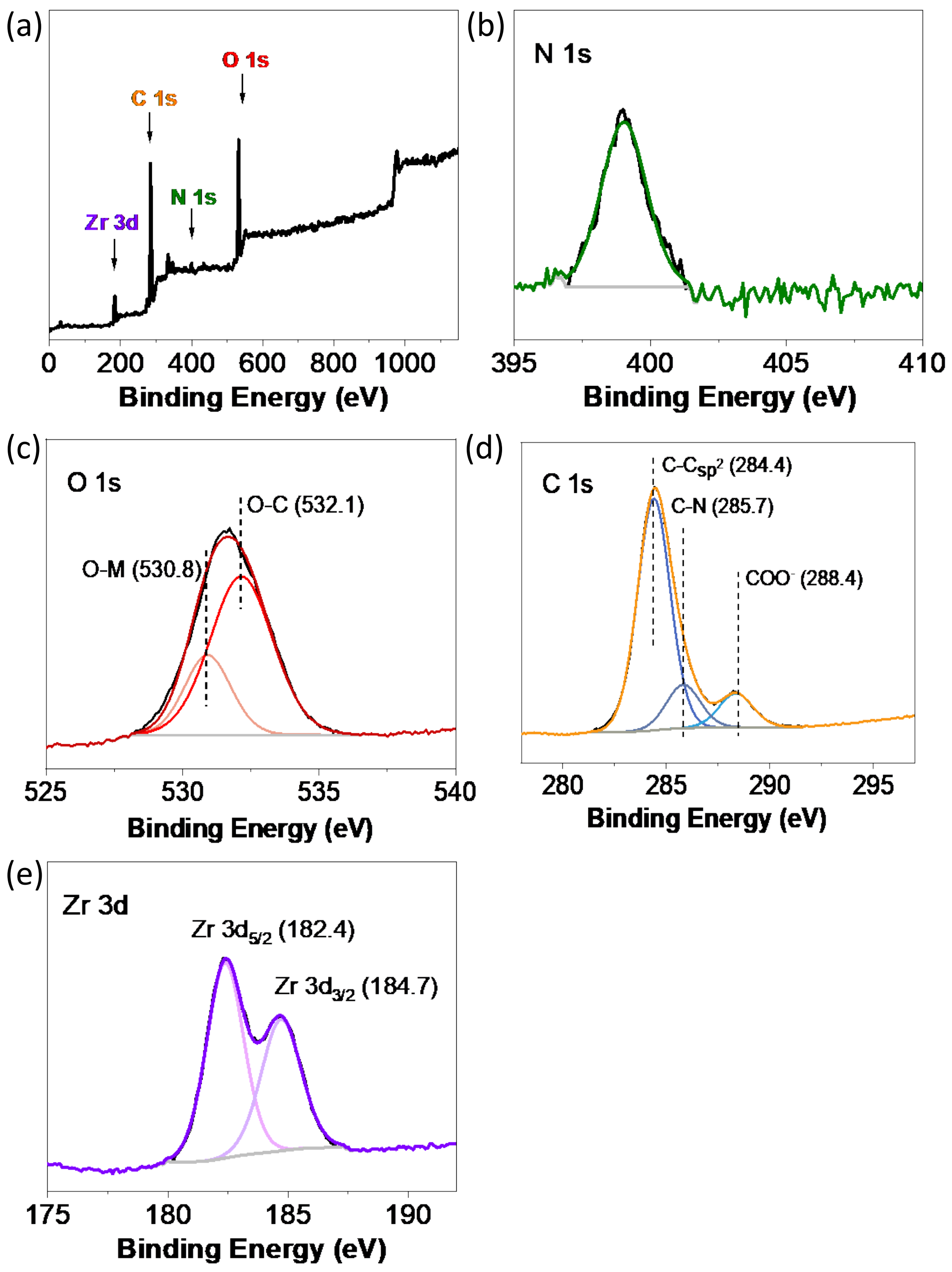

Figure S18. XPS Survey (a), C 1s (b), O 1s (c), N 1s (d) and Zr 3d (e) for UiO-66(Zr)-NH_2.

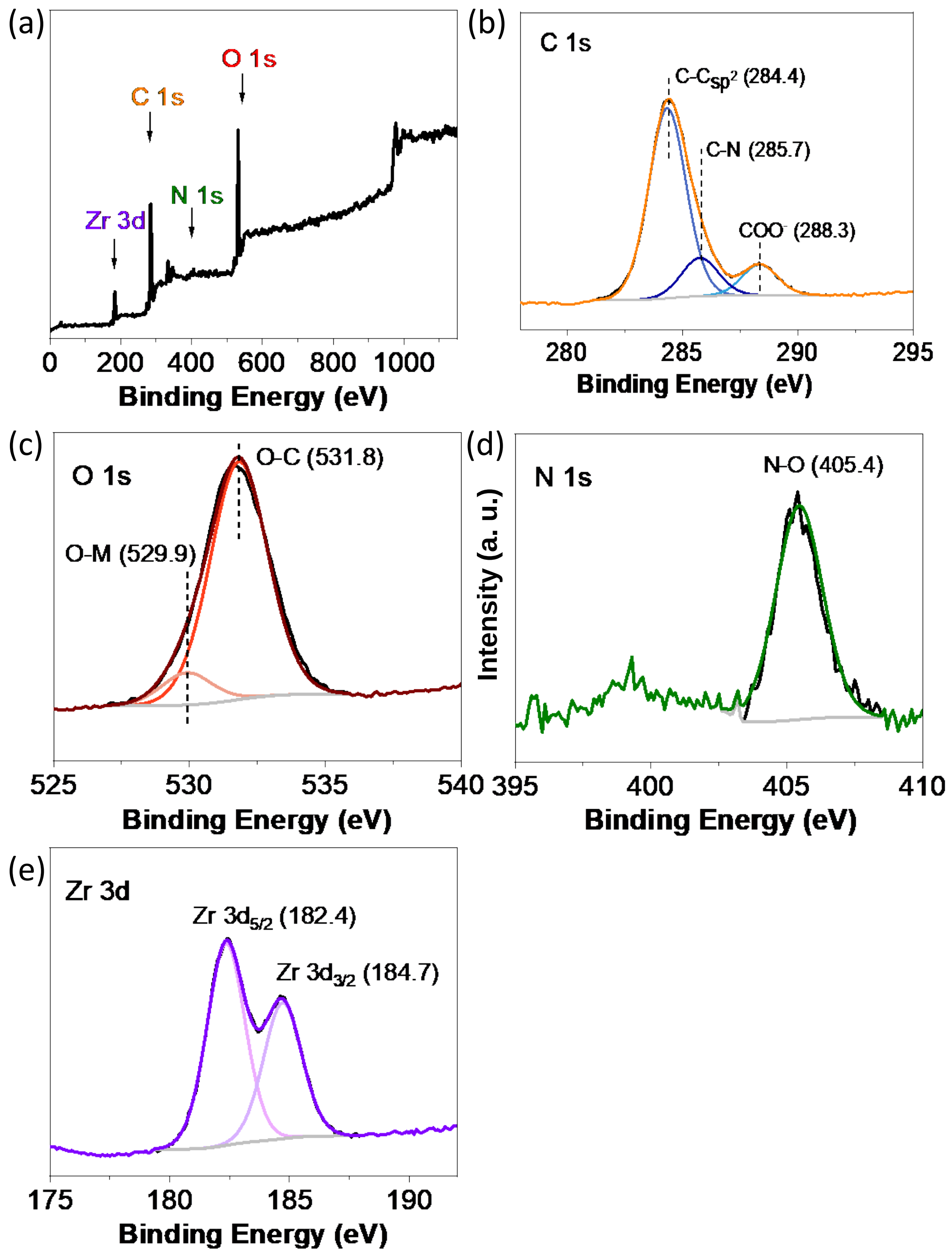

Figure S19. XPS Survey (a), C 1s (b), O 1s (c), N 1s (d) and Zr 3d (e) for UiO-66(Zr)-NO_2.

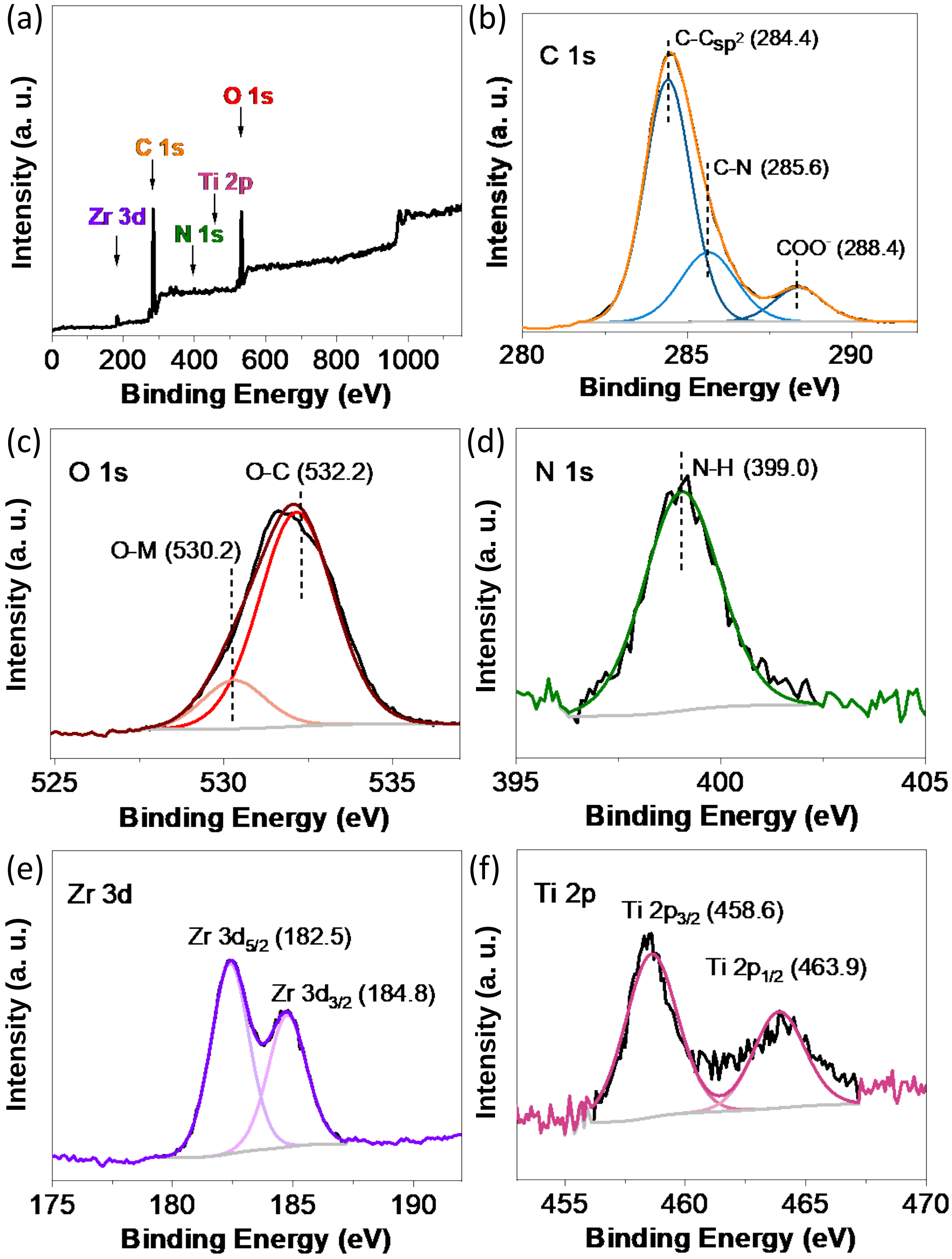

Figure S20. XPS Survey (a), C 1s (b), O 1s (c), N 1s (d) and Zr 3d (e) for UiO-66(Zr/Ti)-NH_2.

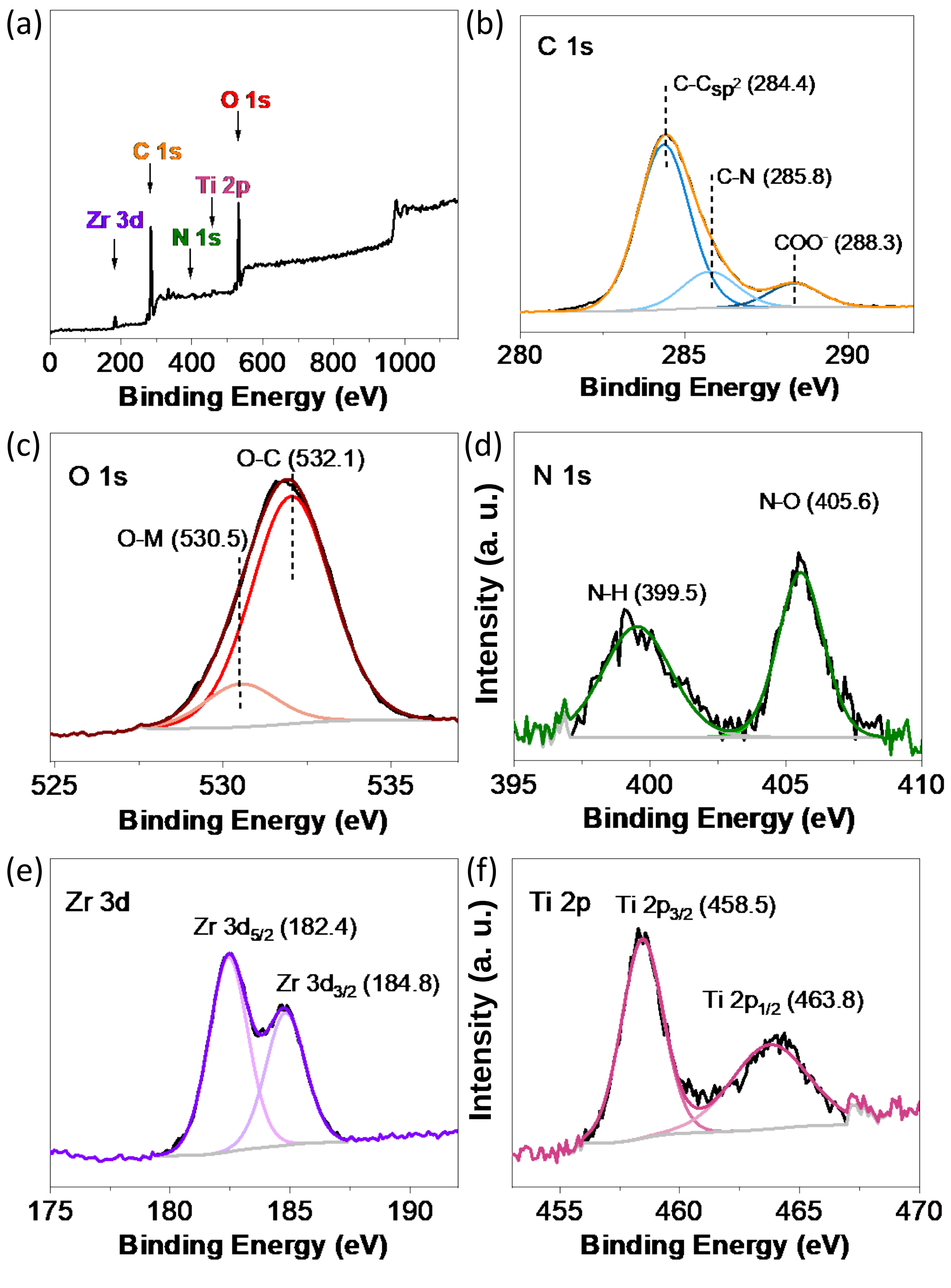

Figure S21. XPS Survey (a), C 1s (b), O 1s (c), N 1s (d) and Zr 3d (e) for UiO-66(Zr/Ti)-NO_2.

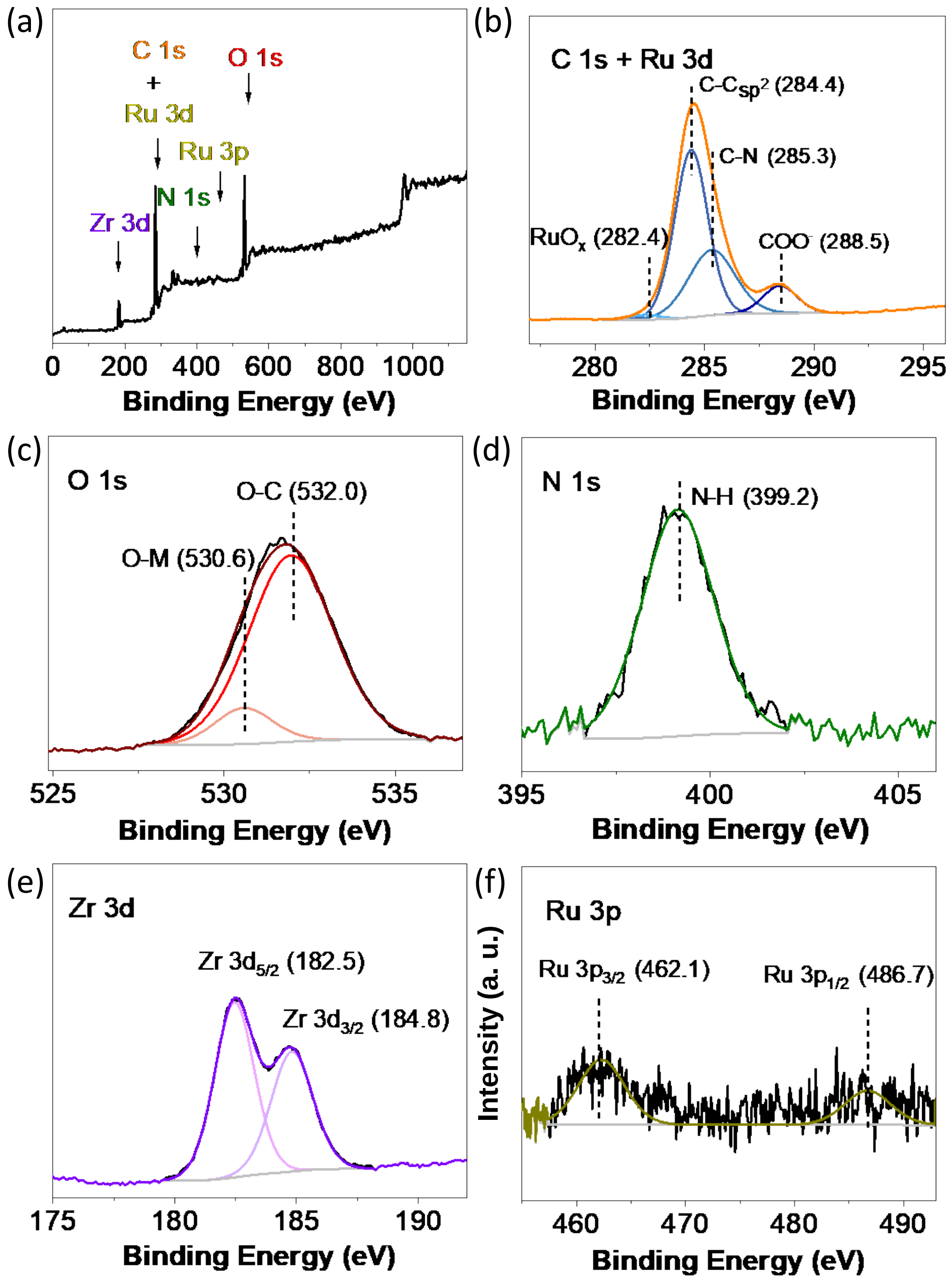

Figure S22. XPS Survey (a), C 1s (b), O 1s (c), N 1s (d) and Zr 3d (e) for RuO_x@UiO-66(Zr)-NH_2.

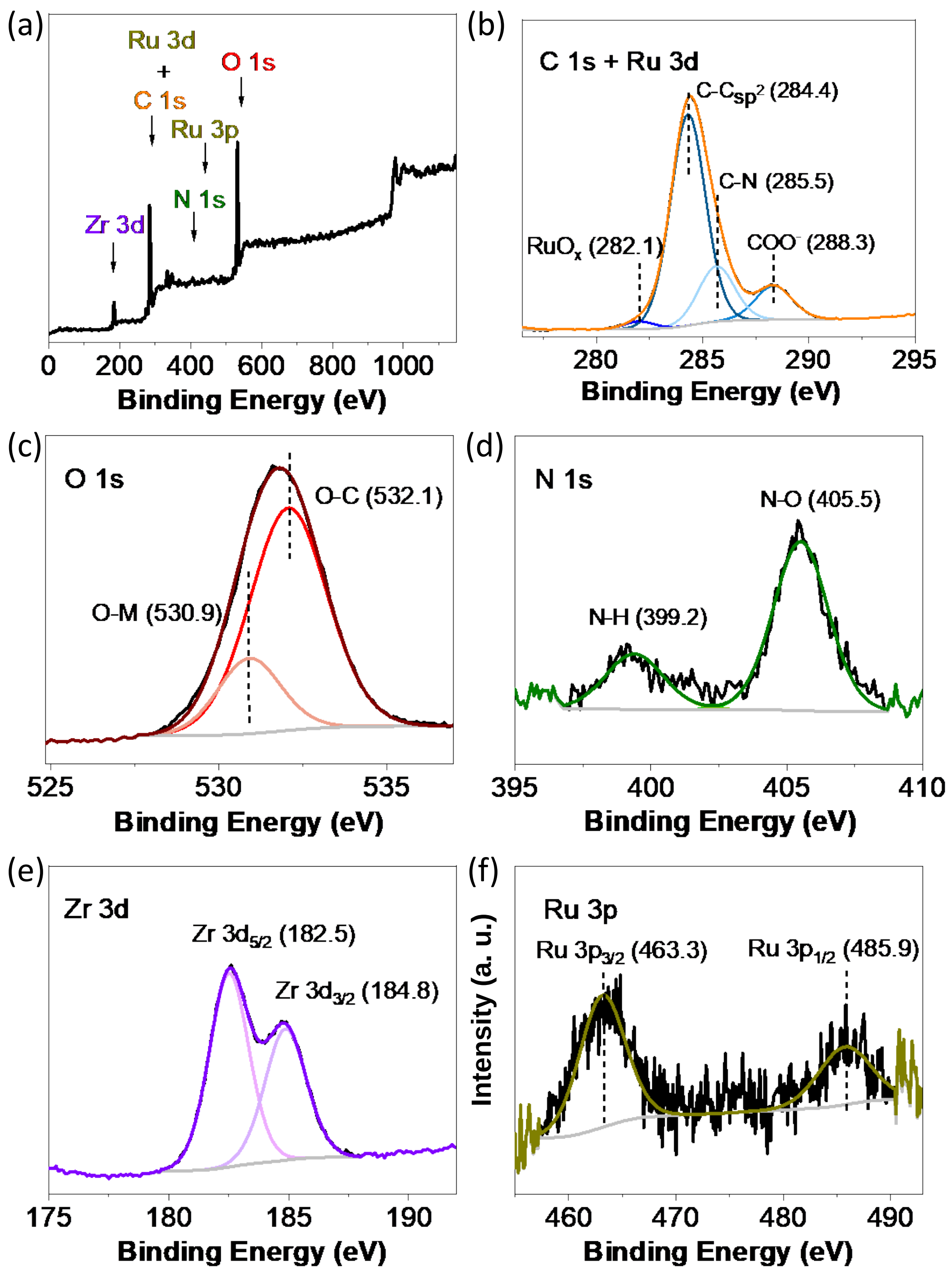

Figure S23. XPS Survey (a), C 1s (b), O 1s (c), N 1s (d) and Zr 3d (e) for RuO_x@UiO-66(Zr)-NO_2.

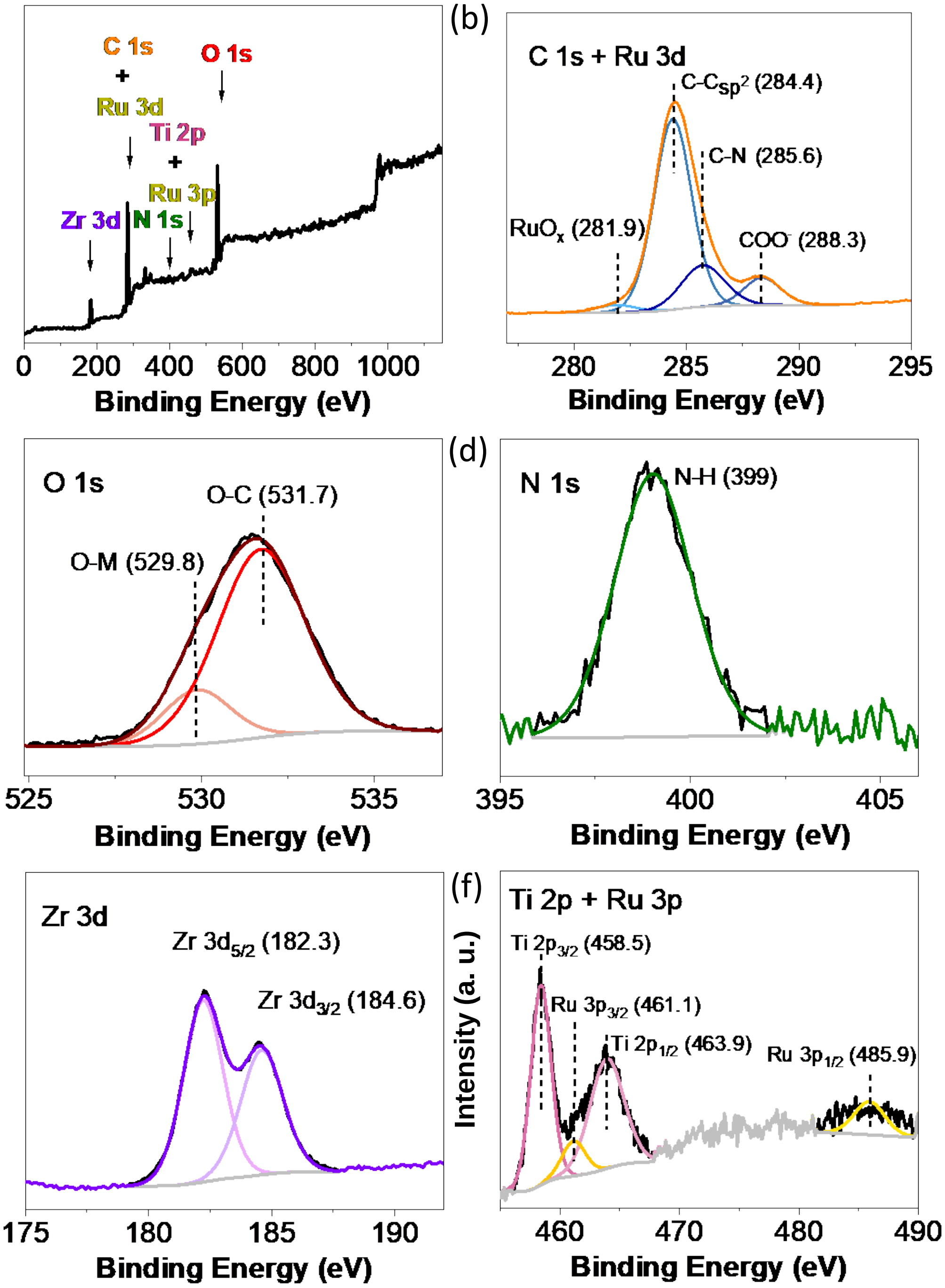

Figure S24. XPS Survey (a), C 1s (b), O 1s (c), N 1s (d) and Zr 3d (e) for RuO_x@UiO-66(Zr/Ti)-NH_2.

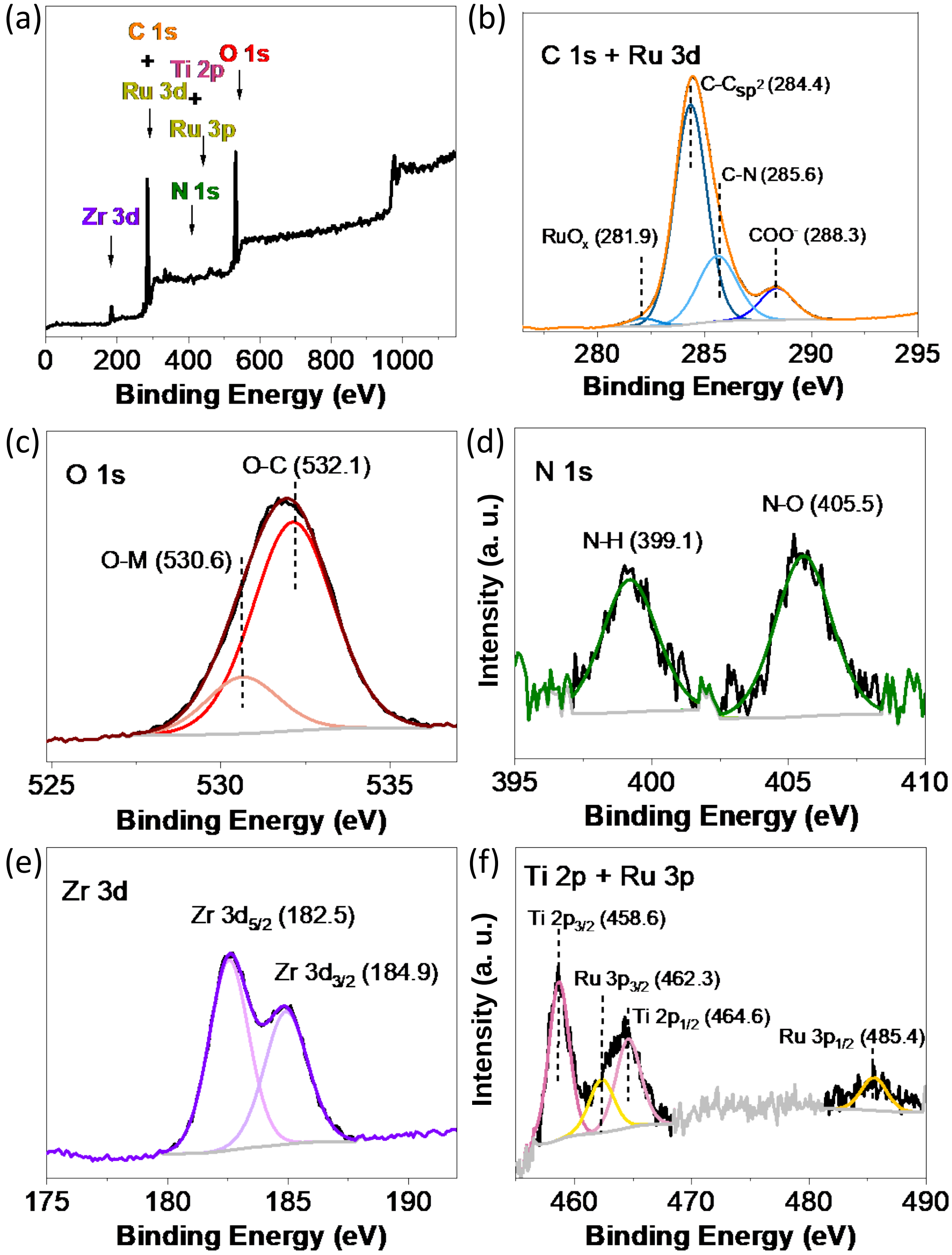

Figure S25. XPS Survey (a), C 1s (b), O 1s (c), N 1s (d) and Zr 3d (e) for RuO_x@UiO-66(Zr/Ti)-NO_2.

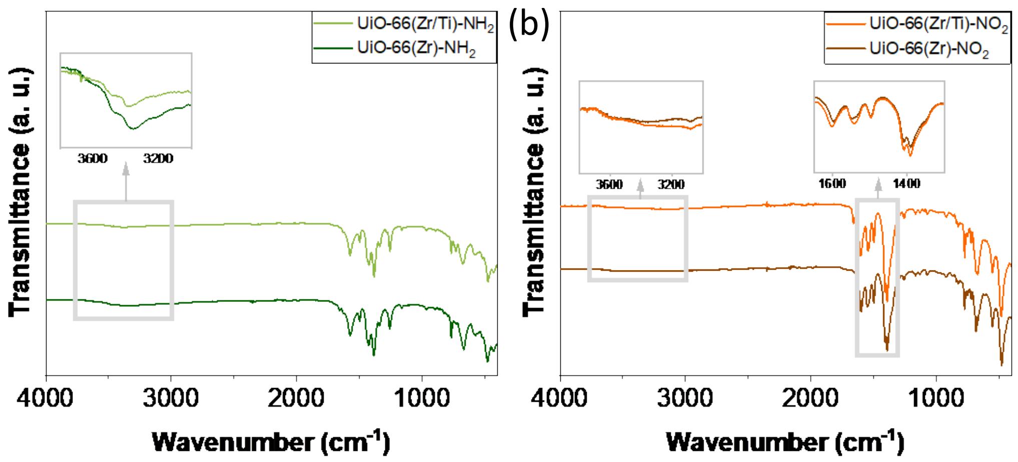

Figure S26. FT-IR of solids as indicated.

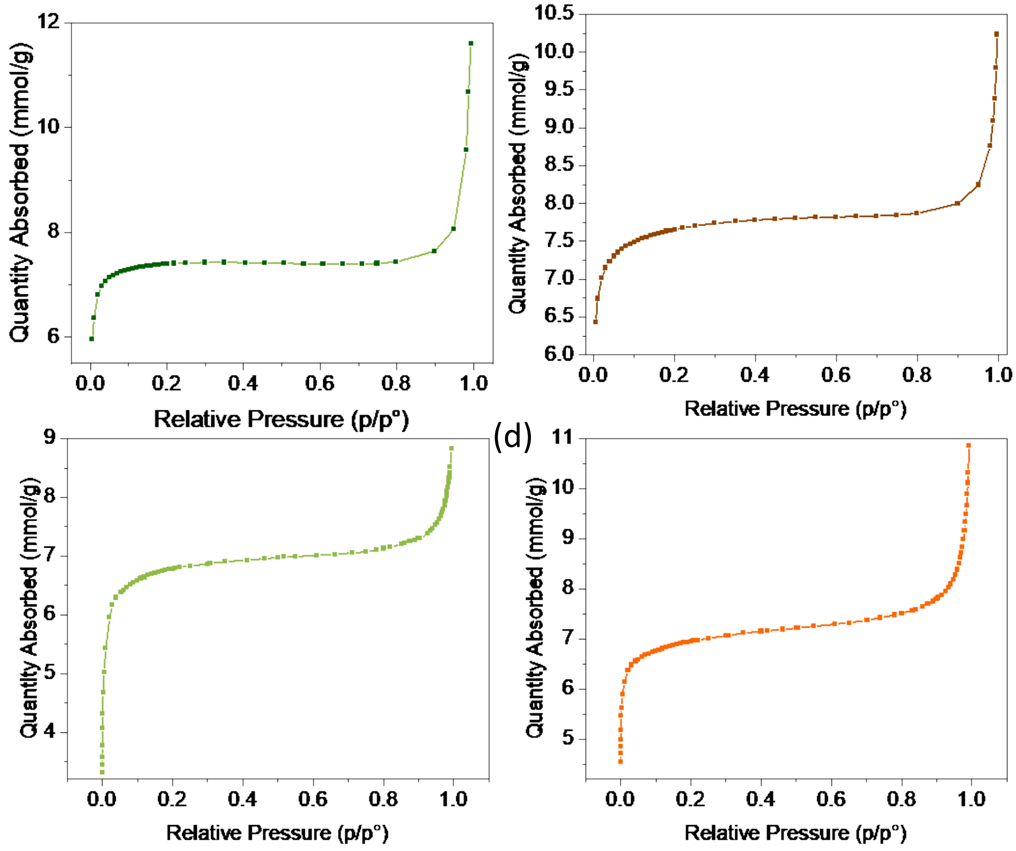

Figure S27. Isothermal N_2 adsorption curve of (a) UiO-66(Zr)-NH_2, (b) UiO-66(Zr)-NO_2, (c) UiO-66(Zr/Ti)-NH_2 and (d) UiO-66(Zr/Ti)-NO_2.

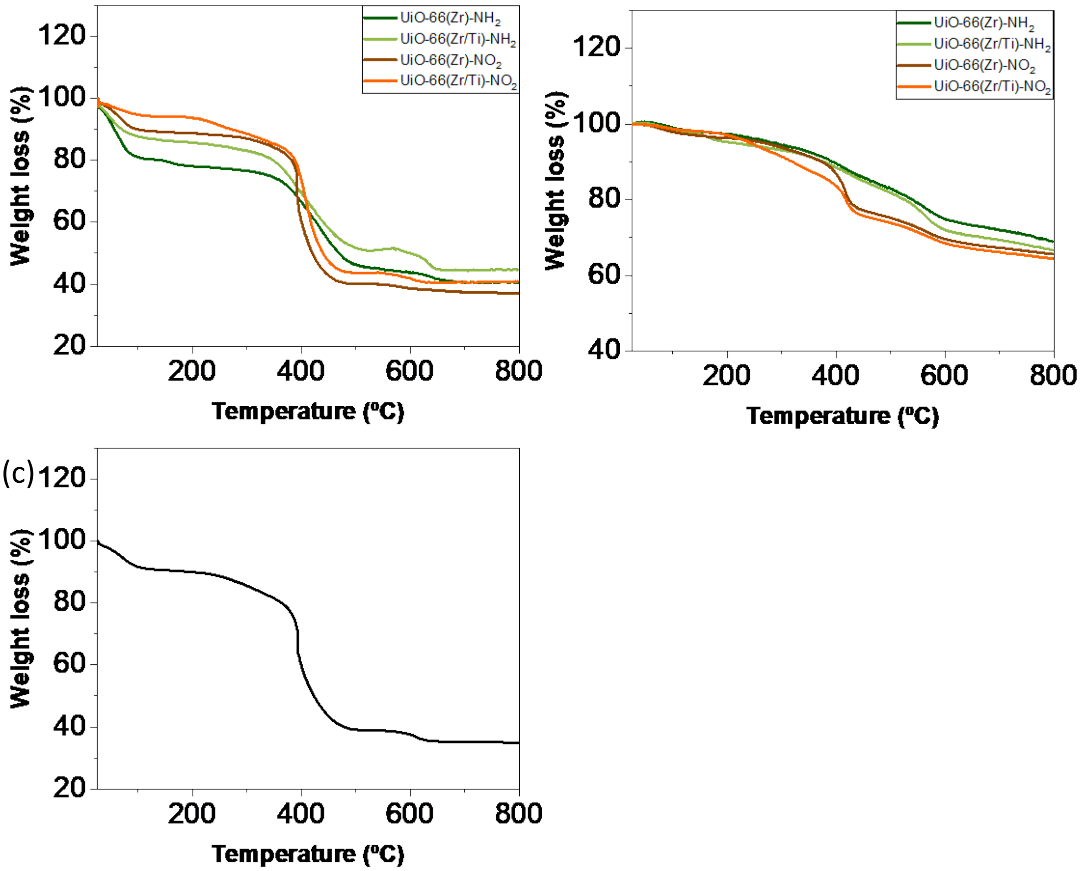

Figure S28. TGA analyses of UiO-66(Zr)-NH_2, UiO-66(Zr)-NO_2, UiO-66(Zr/Ti)-NH_2, UiO-66(Zr/Ti)-NO_2 as indicated under (a) air or (b) N_2 atmosphere. (c) TGA analysis of UiO-66(Zr/Ti)-NO_2, previously submitted to a photocatalytic treatment at 200 ºC under H_2:CO_2 molar ratio for 22 h, under air atmosphere.

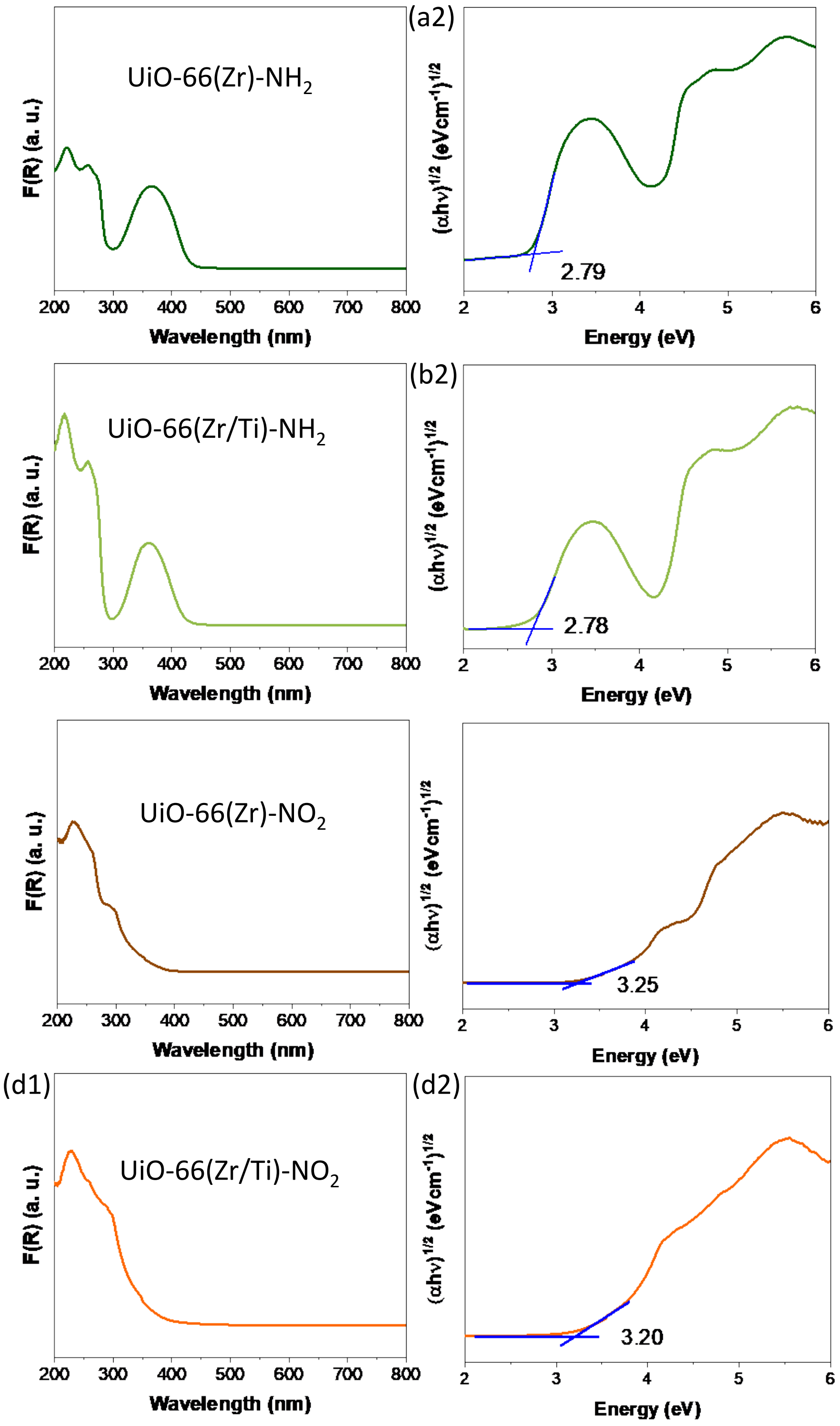

Figure S29. UV-Vis diffuse reflectance (left side) and Tauc plot (right side) of (a) UiO-66(Zr)-NH_2, (b) UiO-66(Zr/Ti)-NH_2, (c) UiO-66(Zr)-NO_2 and (d) UiO-66(Zr/Ti)-NO_2.

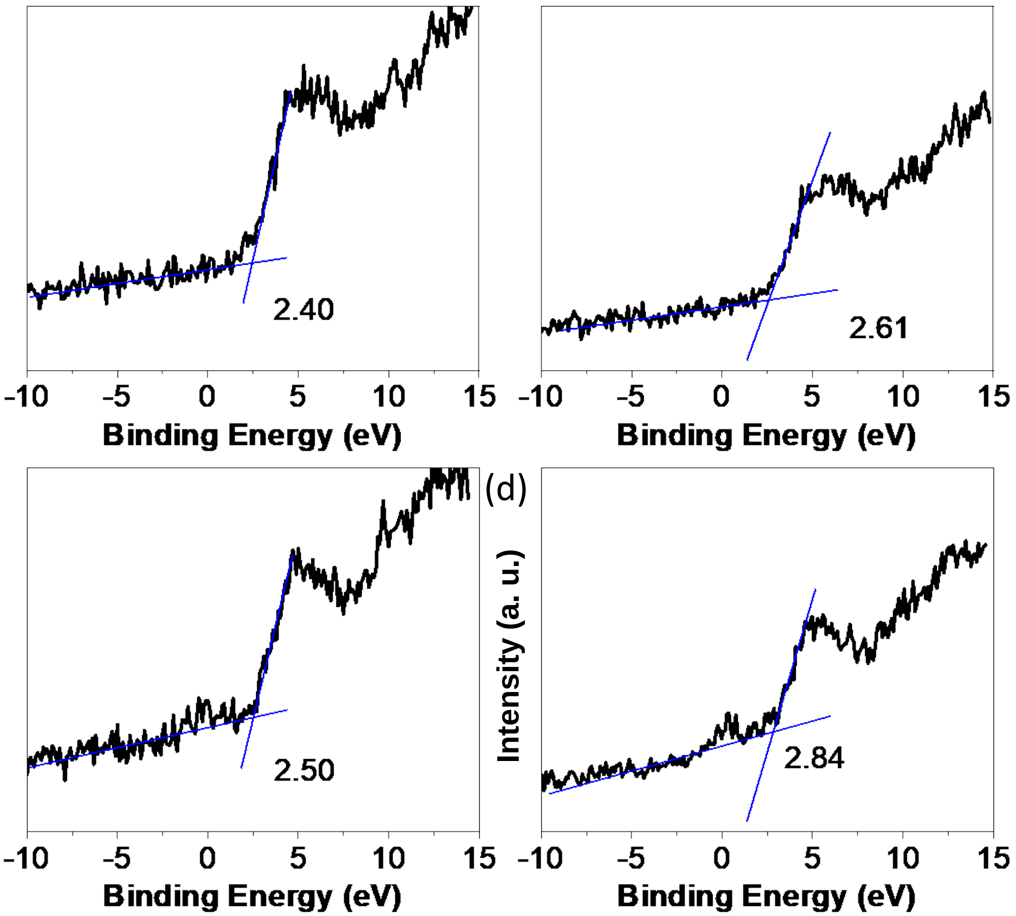

Figure S30. Valence band XPS for (a) UiO-66(Zr)-NH_2, (b) UiO-66(Zr)-NO_2, (c) UiO-66(Zr/Ti)-NH_2, (d) UiO-66(Zr/Ti)-NO_2.

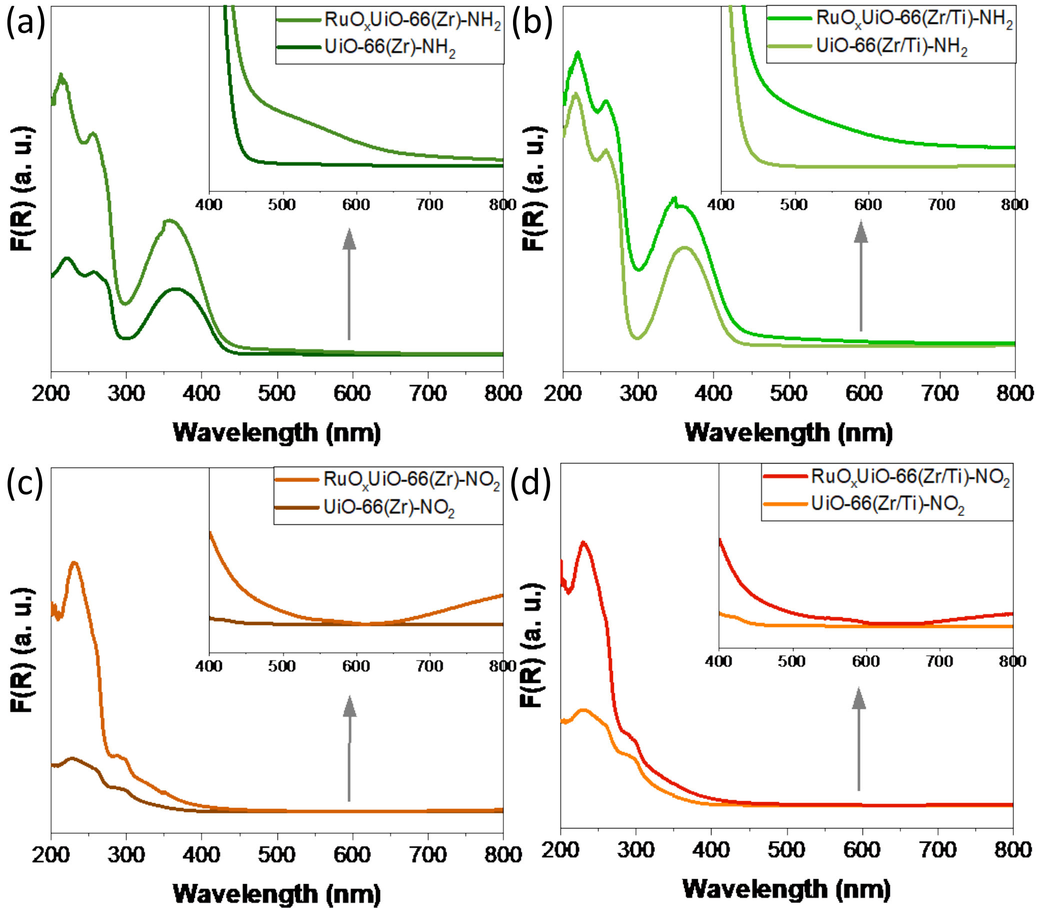

Figure S31. UV-Vis diffuse reflectance of RuO_x NPs on UiO-66 solids: (a) UiO-66(Zr)-NH_2, (b) UiO-66(Zr/Ti)-NH_2, (c) UiO-66(Zr)-NO_2 and (d) UiO-66(Zr/Ti)-NO_2. The inset shows the resonance plasmon band of these nanoparticles.

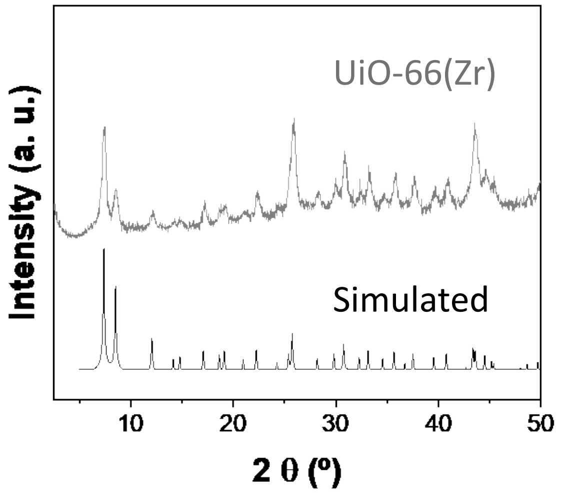

Figure S32. XRD of simulated UiO-66 and PXRD of UiO-66(Zr) material.

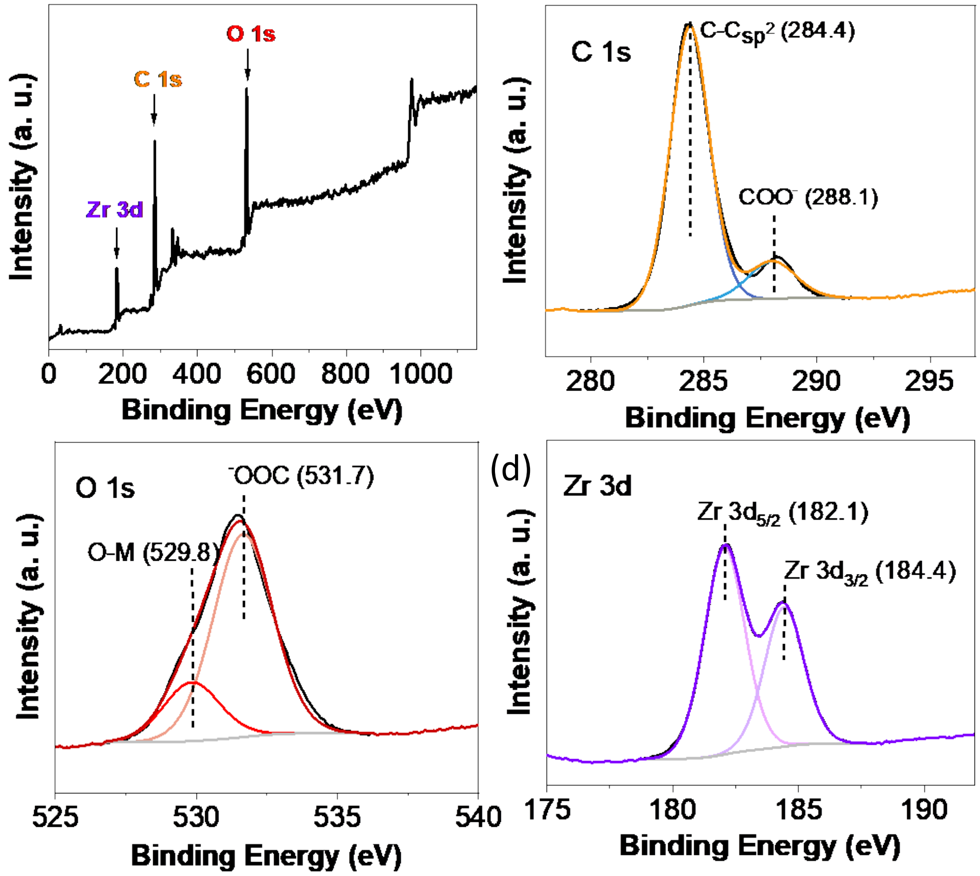

Figure S33. XPS survey (a), C 1s (b), O 1s (c), and Zr 3d (d) of UiO-66(Zr).

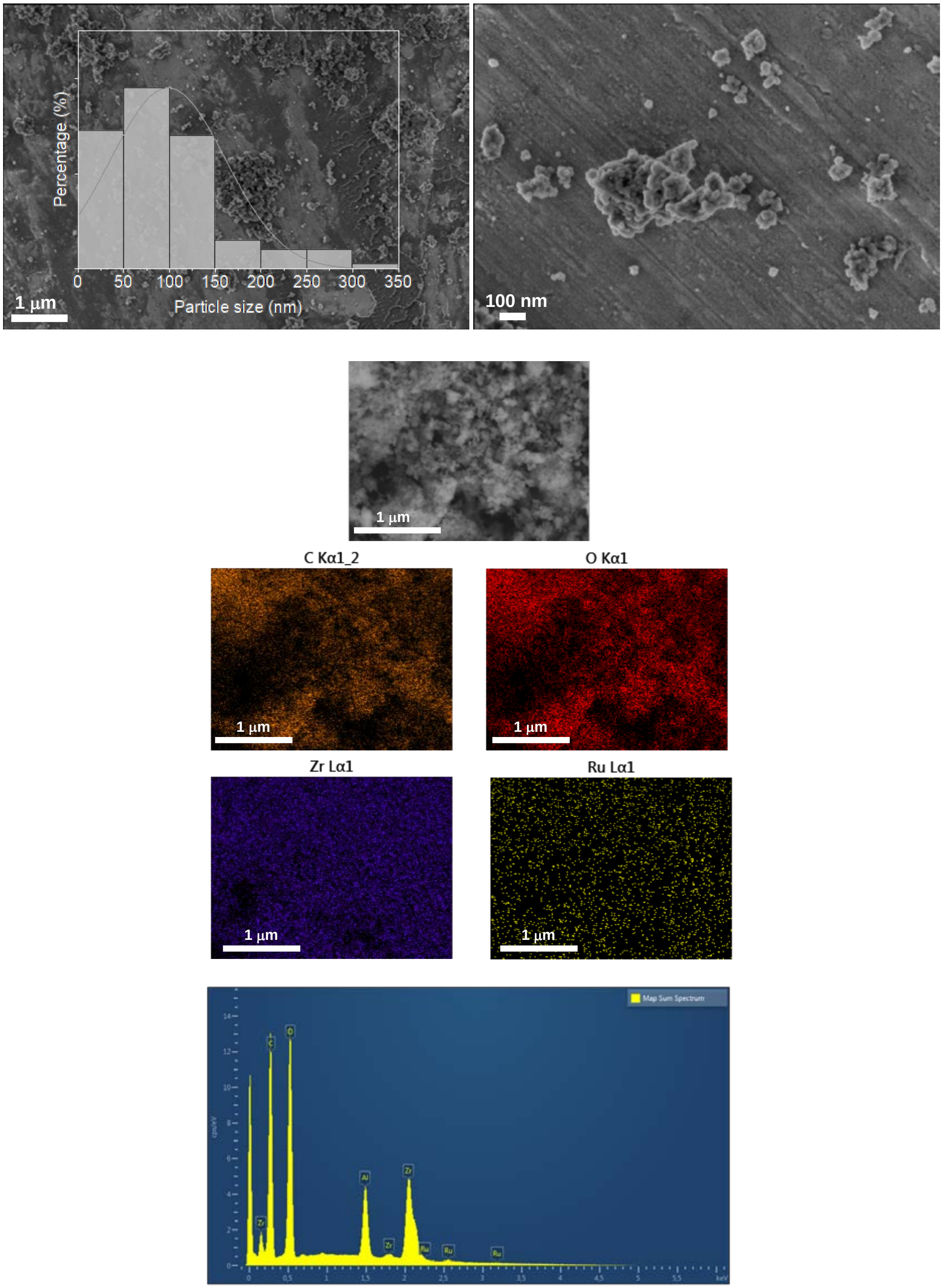

Figure S34. SEM-EDX of RuO_x@UiO-66. The aaverage particle size and standard deviation is 98 ± 63 nm.

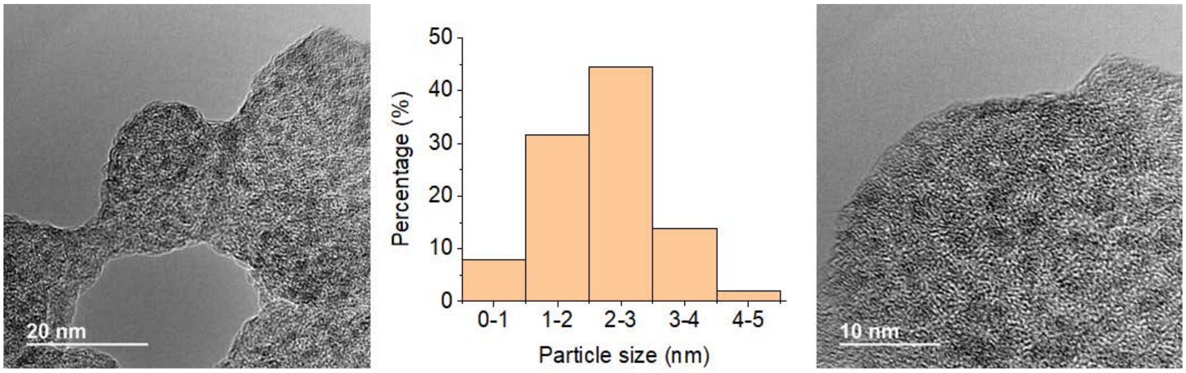

Figure S35. HRTEM image and RuO_x particle size distribution of RuO_x@UiO-66(Zr); average particle size and standard deviation of 2.4 ± 0.8 nm.

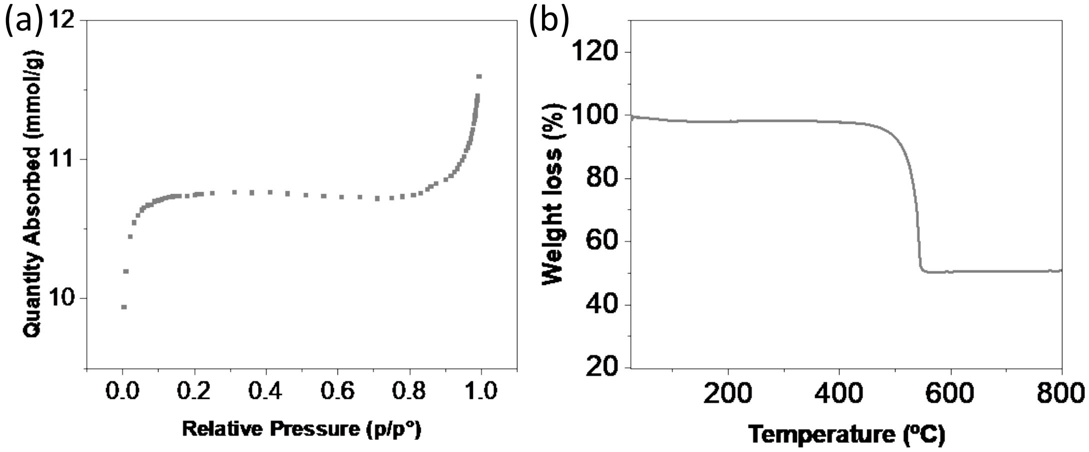

Figure S36. (a) Isothermal N_2 adsorption curve and (b) TGA of UiO-66(Zr).

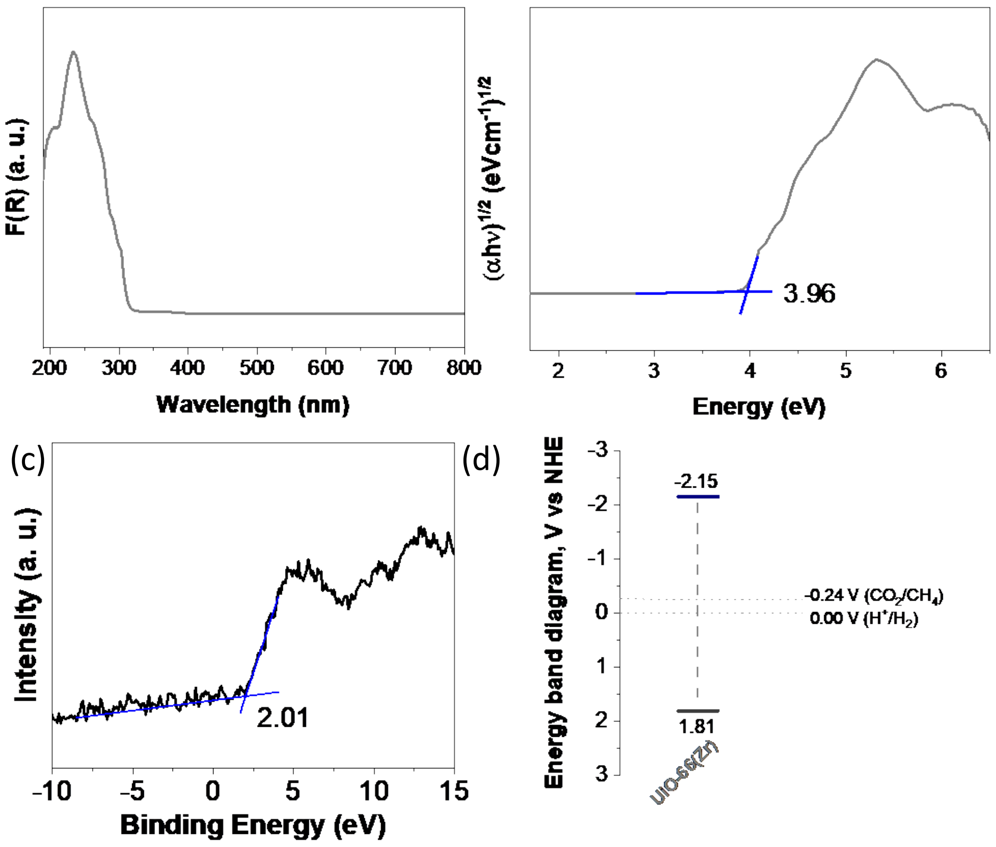

Figure S37. (a) UV-Vis DRS, (b) Tauc plot and, (c) XPS valence band and (d) Energy band level diagram of UiO-66 solid.

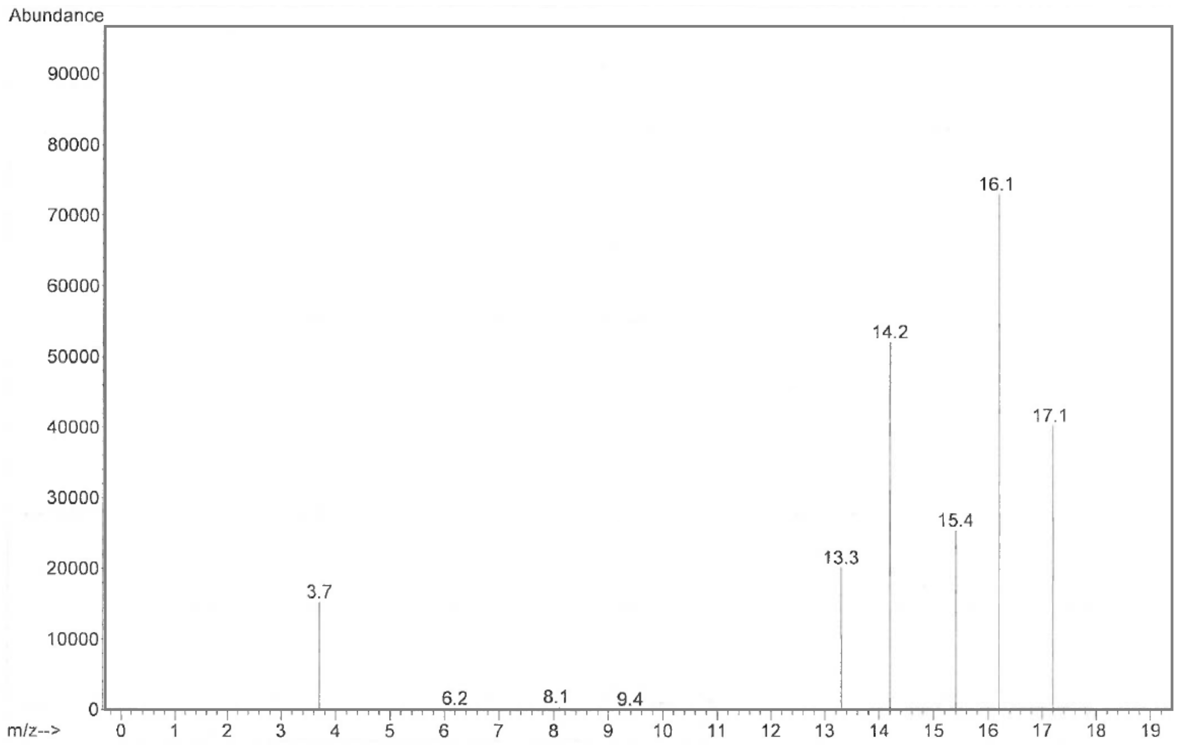

Figure S38. GC-MS obtained after the photocatalytic reaction using $^{13}CO_2$ and RuO_x@UiO-66(Zr/Ti)-NO_2.

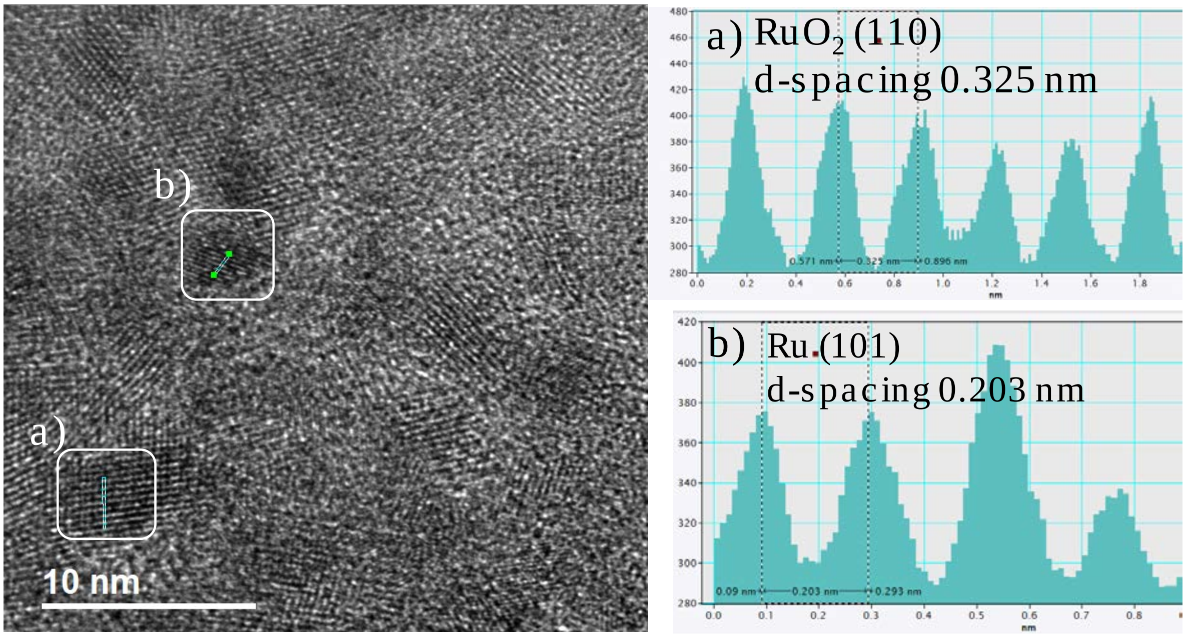

Figure S39. d-spacing estimation from HRTEM image show in main text of the manuscript (Figure 1d).

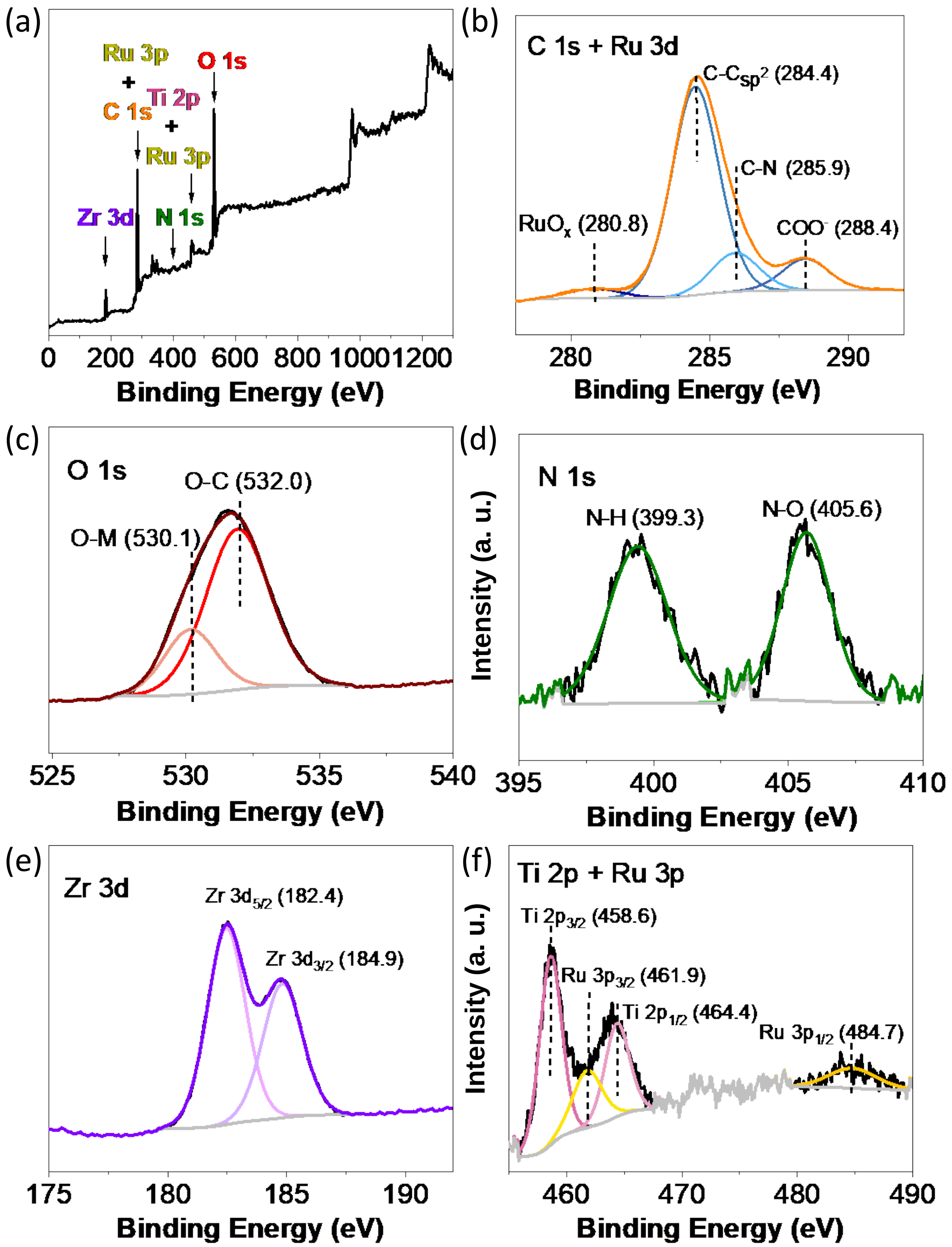

Figure S40. XPS Survey (a), C 1s (b), O 1s (c), N 1s (d) and Zr 3d (e) for used RuO_x@UiO-66(Zr/Ti)-NO_2.

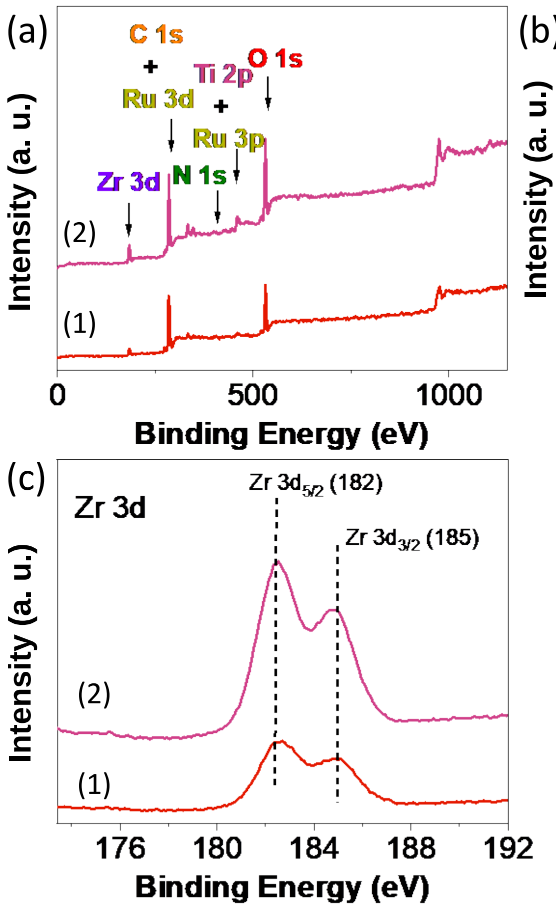

Figure S41. XPS comparison between fresh (red, below) and used (pink, up) Survey (a), O 1s (b), Zr 3d (c) RuO_x@UiO-66(Zr/Ti)-NO_2.

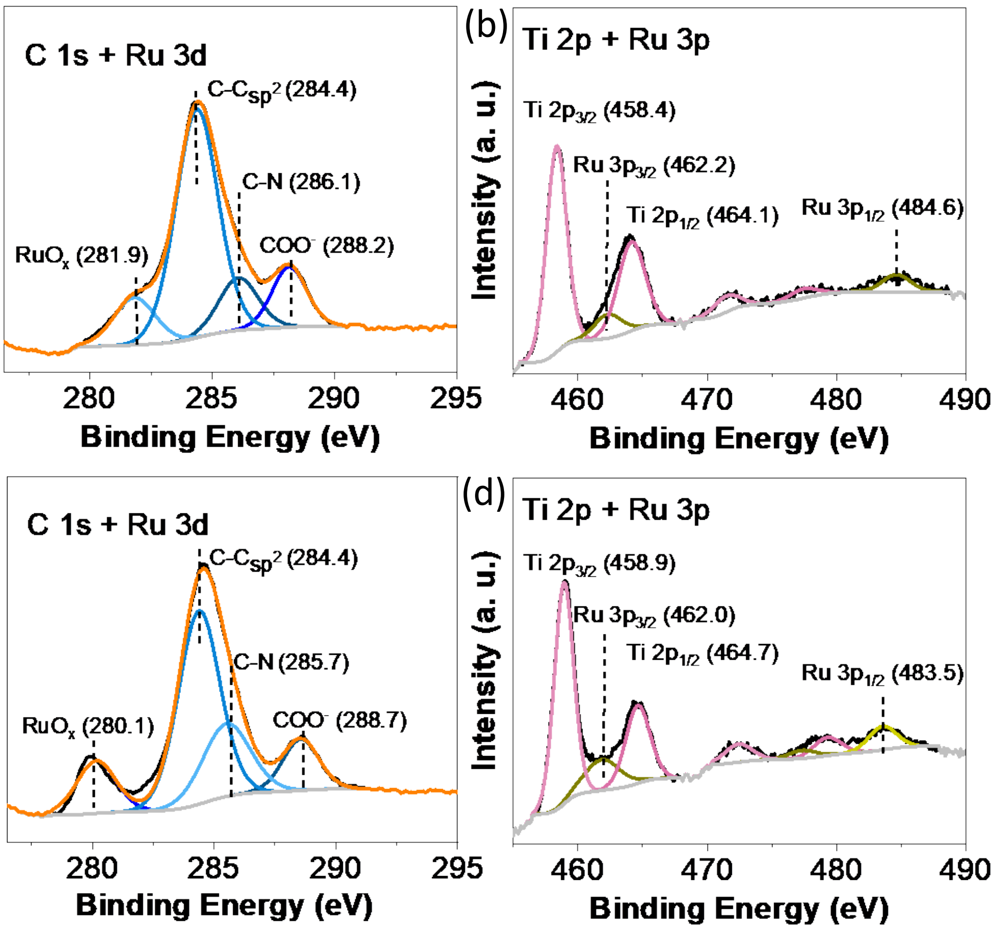

Figure S42. XPS C 1s + Ru 3d (a, c) and XPS Ti 2p + Ru 3p (b, d) of RuO_x@UiO-66(Zr/Ce/Ti) before (a, b) and after a H_2 treatment at 200 ºC for 1 h (c, d)

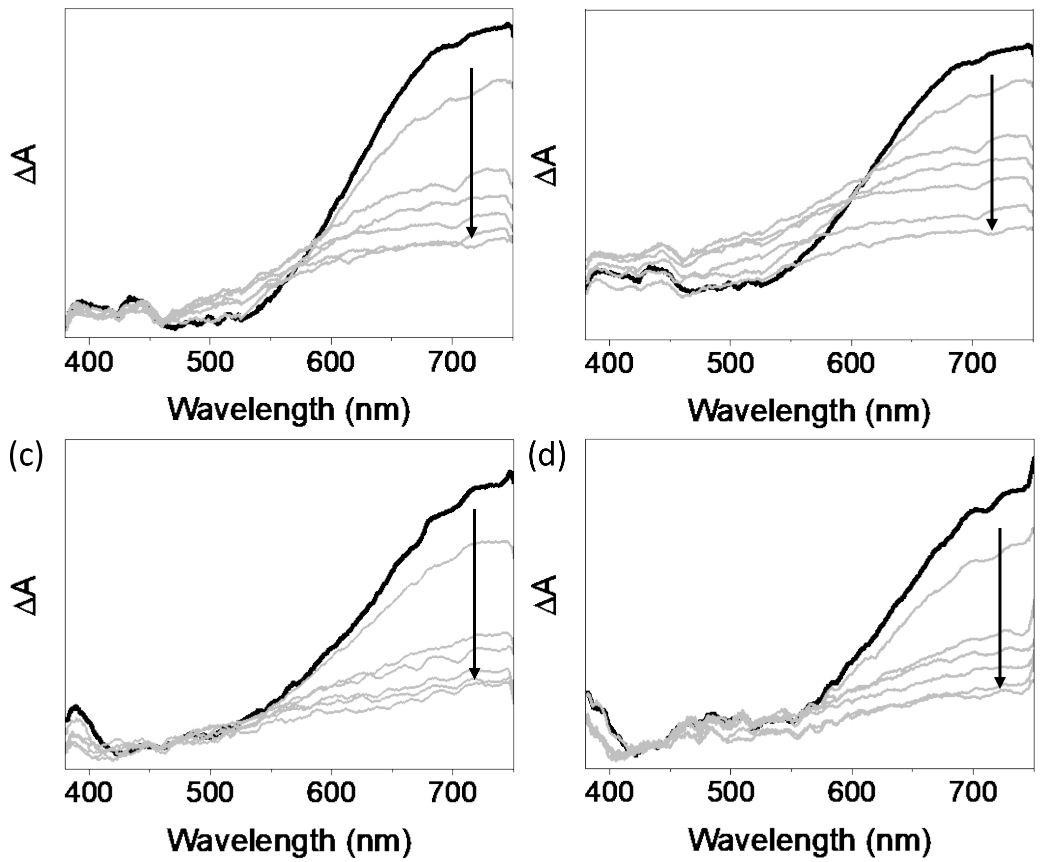

Figure S43. Femtosecond transient absorption spectra from 5 ps to 1 ns for (a) UiO-66(Zr)-NH_2, (b) UiO-66(Zr/Ti)-NH_2, (c) RuO_x@UiO-66(Zr/Ti)-NH_2 and (d) reused RuO_x@UiO-66(Zr/Ti)-NH_2 after excitation at 267 nm in MeCN.

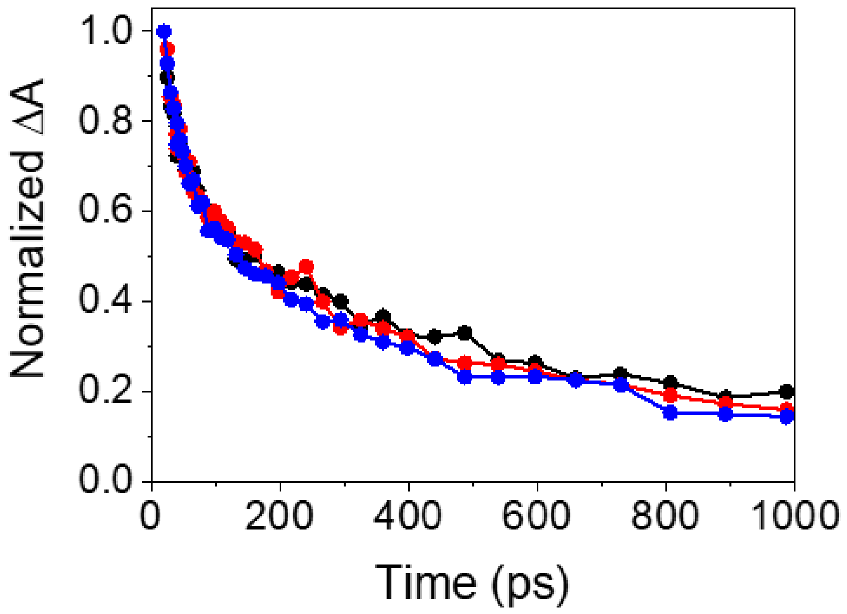

Figure S44. Femtosecond transient absorption decay traces for UiO-66(Zr)-NH_2 (black), @UiO-66(Zr/Ti)-NH_2 (red) and RuO_x@UiO-66(Zr/Ti)-NH_2 (blue) at λ_{exc} = 267 nm in aerated MeCN.

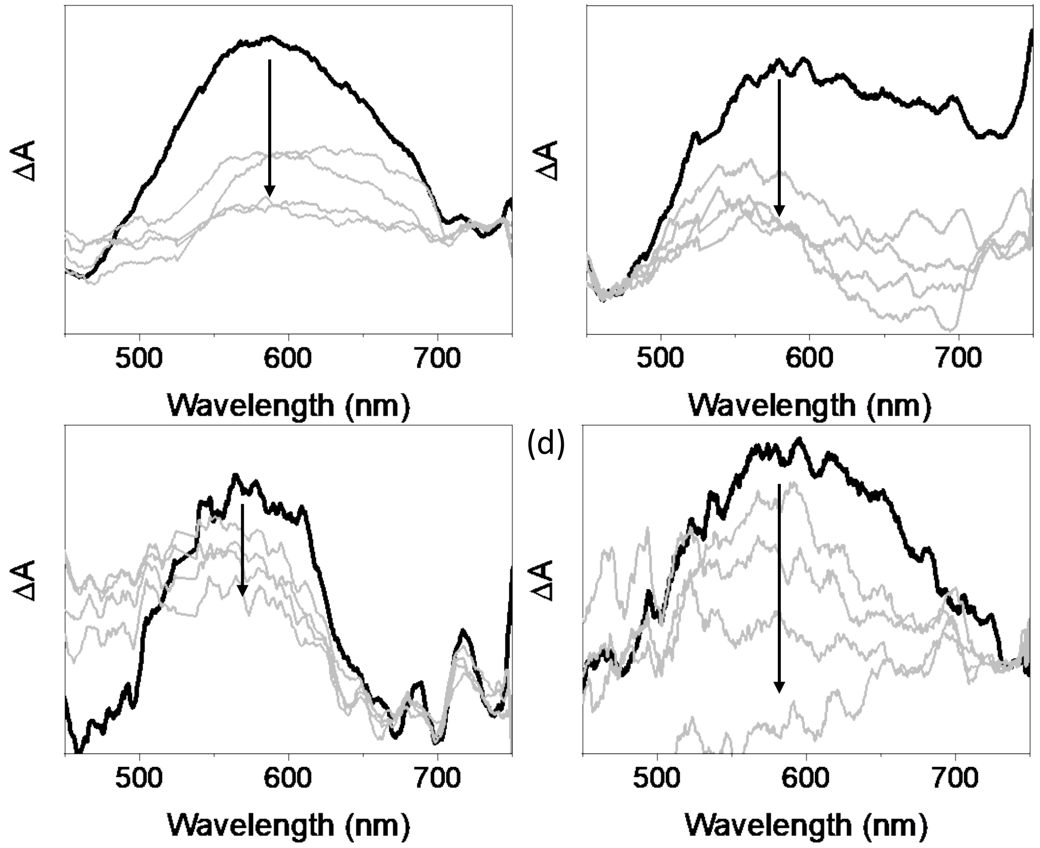

Figure S45. Femtosecond transient absorption spectra from 5 to 100 ps for (a) UiO-66(Zr)-NO_2, (b) UiO-66(Zr/Ti)-NO_2, (c) RuO_x@UiO-66(Zr/Ti)-NO_2 and (d) reused RuO_x@UiO-66(Zr/Ti)-NO_2 after excitation at 267 nm in MeCN.

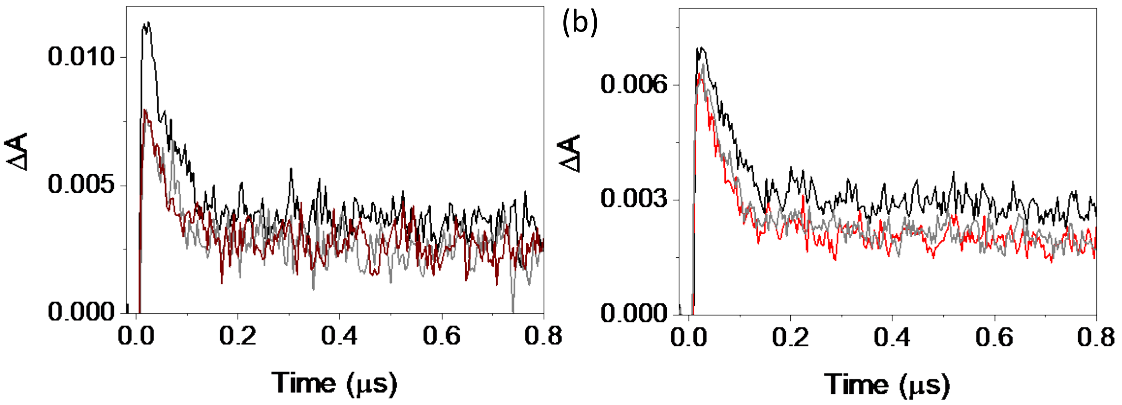

Figure S46. (a) LFP decay traces at 680 nm for UiO-66(Zr)-NO_2 (black), UiO-66(Zr/Ti)-NO_2 (gray) and RuO_x@UiO-66(Zr/Ti)-NO_2 (dark red) after excitation at 266 nm in MeCN under Ar atmosphere. (b) LFP decay traces at 520 nm for UiO-66(Zr/Ti)-NO_2 (black), RuO_x@UiO-66(Zr/Ti)-NO_2 (red) and reused RuO_x@UiO-66(Zr/Ti)-NO_2 (gray) after excitation at 266 nm in MeCN under Ar atmosphere.

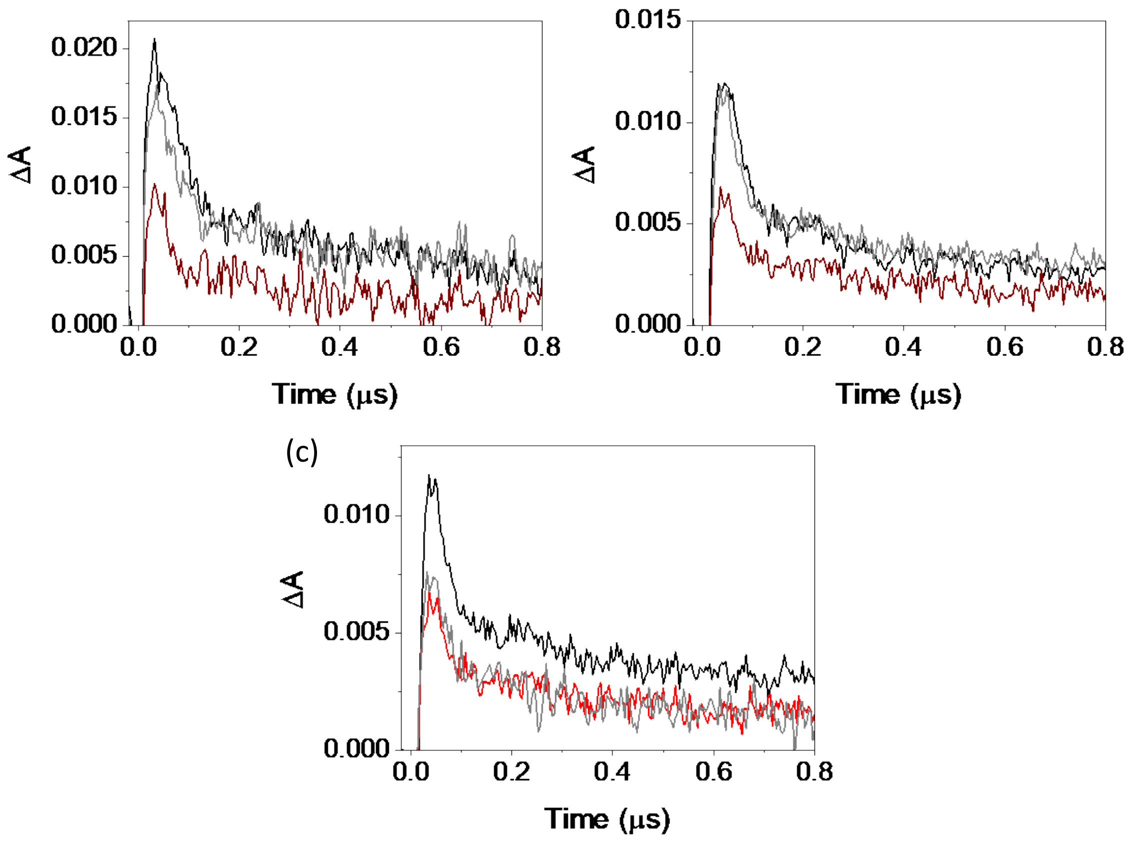

Figure S47. LFP decay traces at (a) 680 nm or (b) 520 nm for UiO-66(Zr)-NH_2 (black), UiO-66(Zr/Ti)-NH_2 (gray) and RuO_x@UiO-66(Zr/Ti)-NH_2 (dark red) after excitation at 266 nm in MeCN under Ar atmosphere, and (c) 520 nm for UiO-66(Zr/Ti)-NH_2 (black), RuO_x@UiO-66(Zr/Ti)-NH_2 (red) and reused RuO_x@UiO-66(Zr/Ti)-NH_2 (gray) after excitation at 266 nm in MeCN under Ar atmosphere.

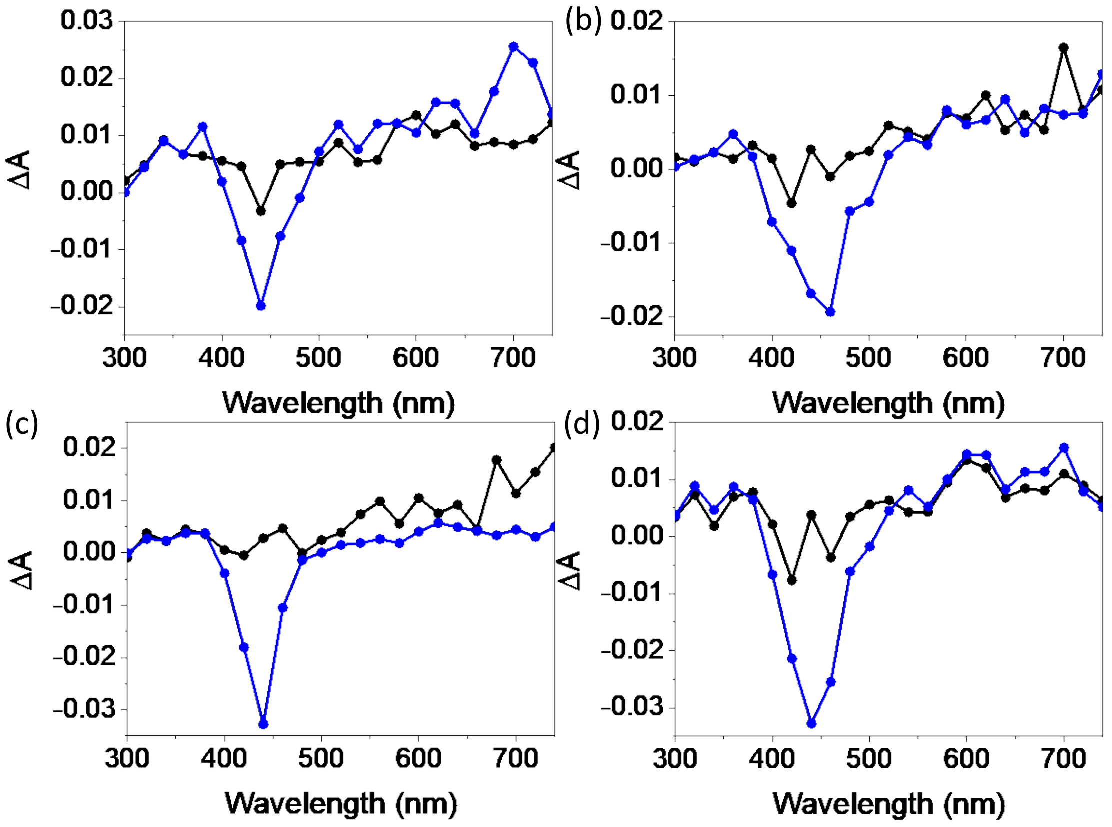

Figure S48. LFP spectra in acetonitrile (black) and in acetonitrile in the presence of 20% methanol (blue) recorded 32 ns after the laser pulse for (a) UiO-66(Zr)-NH_2, (b) UiO-66(Zr/Ti)-NH_2, (c) RuO_x@UiO-66(Zr/Ti)-NH_2 and (d) reused RuO_x@UiO-66(Zr/Ti)-NH_2. All measurements were performed in argon atmosphere at λ_{exc} = 266 nm.

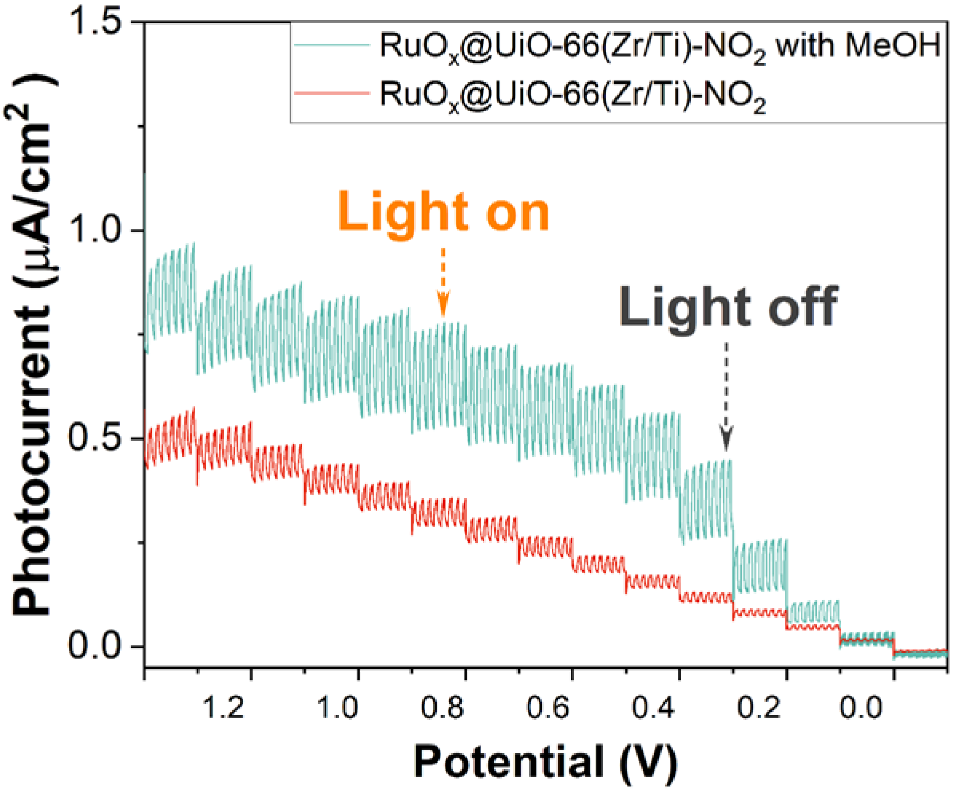

Figure S49. Current respond of RuO_x@UiO-66(Zr/Ti)-NO_2 supported on a carbon paper substrate electrode and immersed in acetonitrile (0.1 M, NBu_4PF_6) solution or in a mixture of acetonitrile and methanol (0.3 mL MeOH) solution upon polarization from 1.3 to -0.1 V vs. Ag/AgCl during consecutive on/off cycles with simulated sunlight.

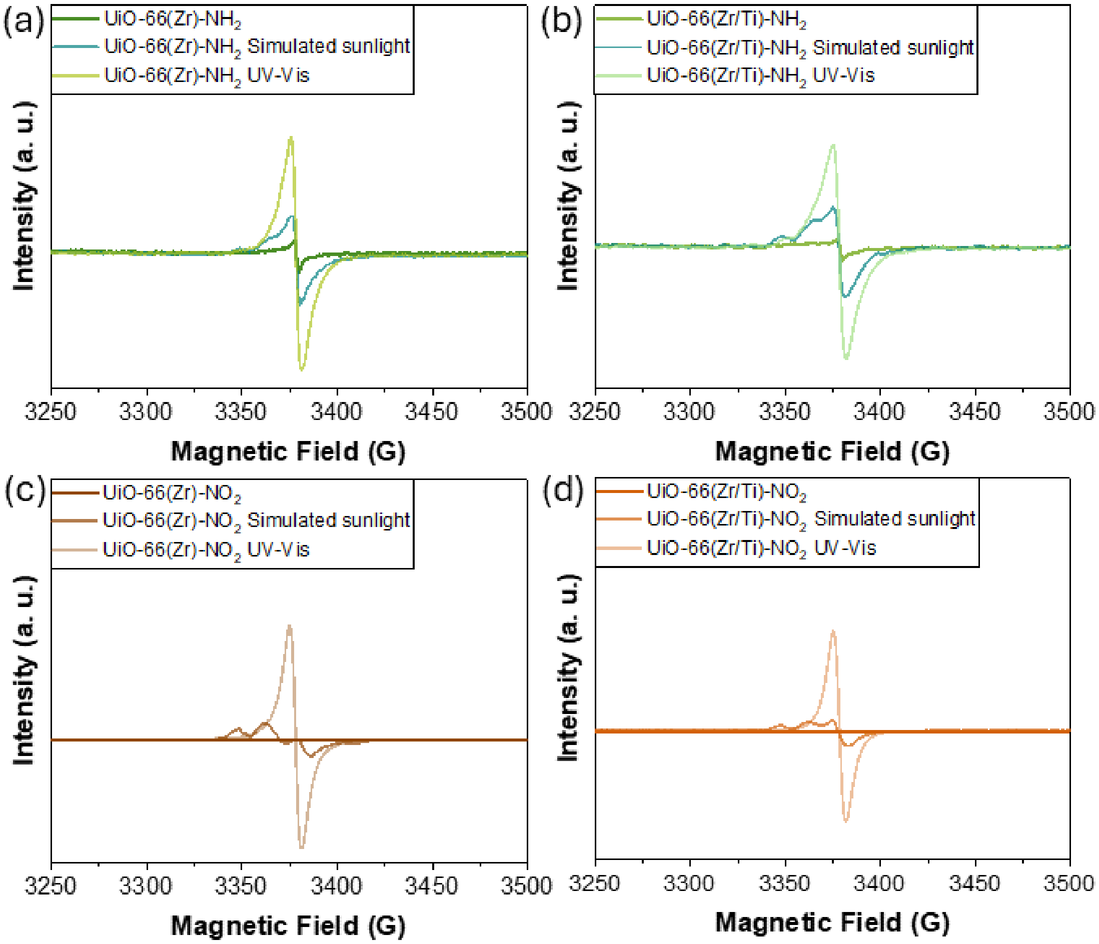

Figure S50. ESR spectra of UiO-66(Zr)-NH_2 (a), UiO-66(Zr/Ti)-NH_2 (b), UiO-66(Zr)-NO_2 (c) or UiO-66(Zr/Ti)-NO_2 (d) under dark or irradiation conditions as indicated.

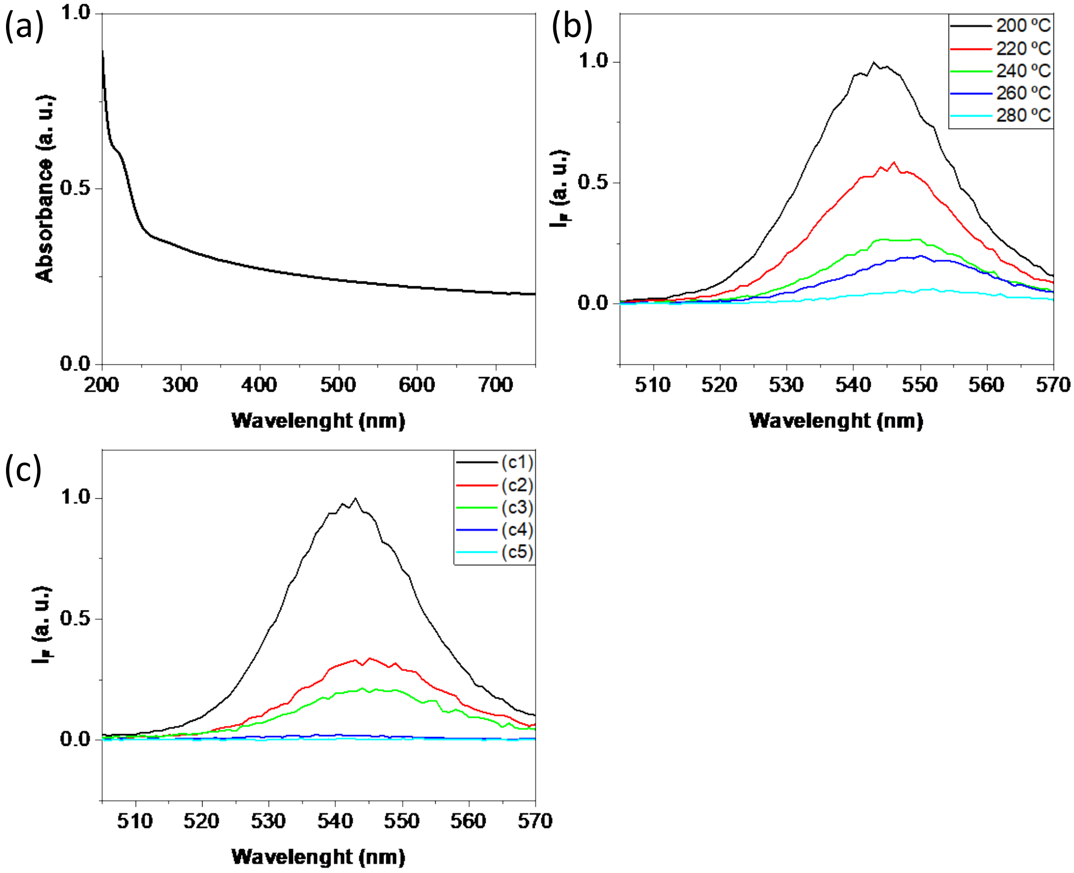

Figure S51. (a) UV-Vis spectrum of CdSe/ZnS QDs suspended in acetonitrile. (b) Photoluminescence spectra of CdSe/ZnS QDs supported on a quartz holder upon excitation at 450 nm and previously heated at different temperatures from 200 to 280 ºC as indicated. (c) Photoluminescence spectra CdSe/ZnS QDs supported on a quartz holder upon excitation at 450 nm and previously heated at 200 ºC under dark conditions (c1) or under simulated sunlight irradiation with an irradiance of (c2) 85, (c3) 140, (c4) 220, (c5) 385 mW/cm^2.

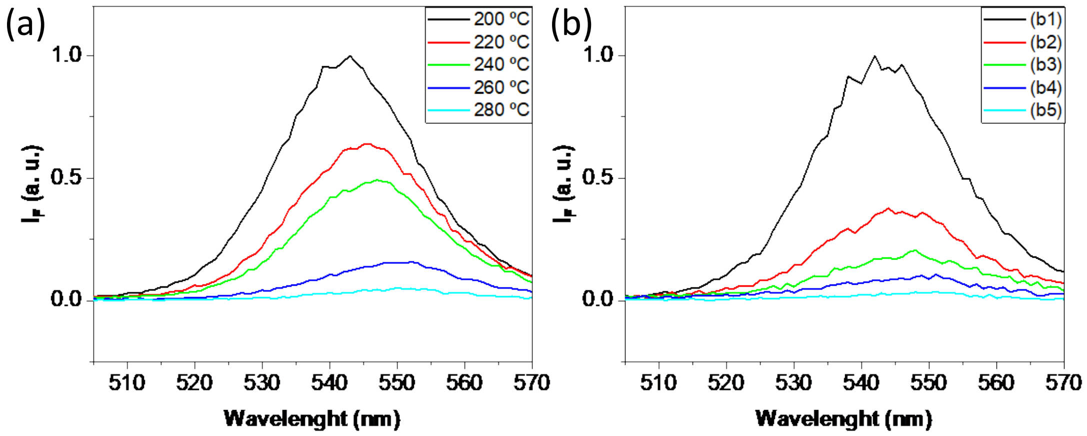

Figure S52. (a) Photoluminescence spectra of used RuO_x@UiO-66(Zr/Ti)-NO_2 supported CdSe/ZnS QDs deposited on a quartz holder upon excitation at 450 nm and previously heated at different temperatures from 200 to 280 ºC as indicated. (b) Photoluminescence spectra of used RuO_x@UiO-66(Zr/Ti)-NO_2 supported CdSe/ZnS QDs deposited on a quartz holder upon excitation at 450 nm and previously heated at 200 ºC under dark conditions (b1) or under simulated sunlight irradiation with an irradiance of (b2) 85, (b3) 140, (b4) 220, (b5) 385 mW/cm^2.

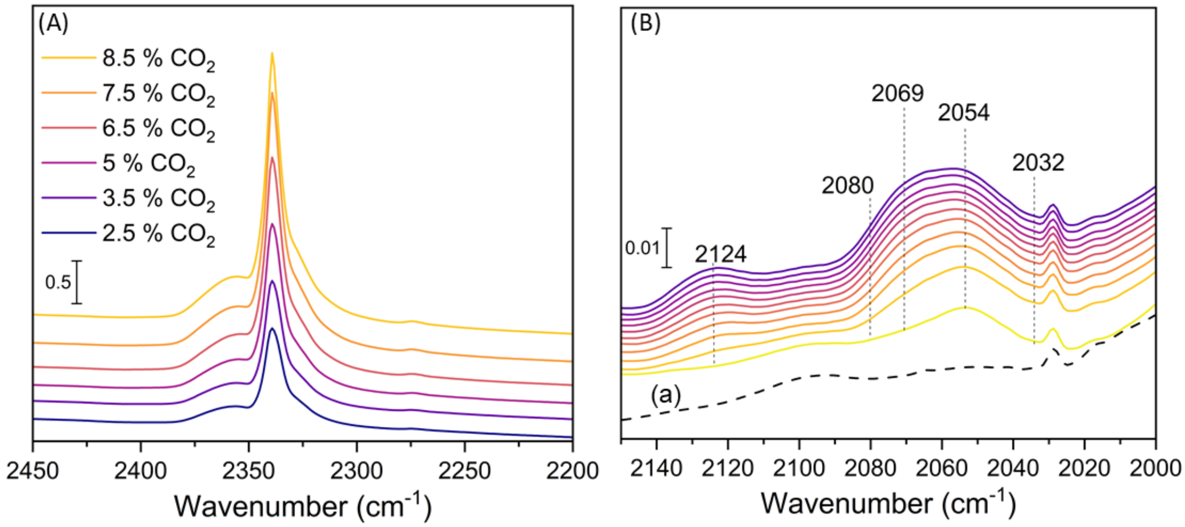

Figure. S53 (a) Direct spectra of chemisorbed CO_2 over RuO_x@UiO-66(Zr/Ti)-NO_2 versus diffrent concentration of CO_2 in Argon and (b) the direct spectra of asdsorbed CO (0.05 %) over RuO_x@UiO-66(Zr/Ti)-NO_2 versus time (1) is the reference spectrum recorded after activation uncer H_2 at room temperature. The assigments of the different bands are summarized in Table S3.

Table S1: Photocatalytic CO_2 methanation by H_2 using MOF-based photocatalysts reported in the literature.

Entry	Photocatalyst	Co-catalyst	Irradiation source	Reaction conditions	CH_4 production (mmol g^{-1})	Ref.
1	UiO-66(Zr/Ti)-NO_2	RuO_x (1 wt%)	Simulated solar light irradiation (150 W Hg-Xe lamp equipped with an AM 1.5 filter)	$P(H_2)$=1.3bar, $P(CO_2)$=0.2 bar, 200ºC, 22 h	5.03	Present work
2	UiO-66(Zr/Ce/Ti)	RuO_x (1 wt%)	Simulated solar light irradiation (150 W Hg-Xe lamp equipped with an AM 1.5 filter)	$P(H_2)$=1.05bar, $P(CO_2)$=0.25 bar, 200ºC, 22 h	1.80	7
3	MIL-125(Ti)-NH_2	RuO_x (1 wt%)	Simulated solar light irradiation (150 W Hg-Xe lamp equipped with an AM filter)	$P(H_2)$=1.05bar, $P(CO_2)$=0.25 bar, 200ºC, 22 h	0.92	6
4	MIL-125(Ti)-NH_2	RuO_x (2 wt%)	Simulated solar light irradiation (150 W Hg-Xe lamp equipped with an AM 1.5 filter)	$P(H_2)$=1.05bar, $P(CO_2)$=0.25 bar, 200ºC, 22 h	2.20	6
5	MIP-208(Ti)	RuO_x (0.76 wt%)	Simulated solar light irradiation (150 W Hg-Xe lamp equipped with an AM filter)	$P(H_2)$=1.05bar, $P(CO_2)$=0.25 bar, 200ºC, 22 h	0.79	8
6	MOF-Zn(1)	Cu_2O (1 wt%)	UV-Vis light irradiation (300 W Xe lamp)	$P(H_2)$=1.05bar, $P(CO_2)$=0.25 bar, 215ºC, 24 h	0.046	9

**Table S2.** Global fit analysis			
	τ_1 (ps)	τ_2 (ps)	τ_{avg} (ps)
UiO-66(Zr)-NH_2	18	597	408
UiO-66(Zr/Ti)-NH_2	27	830	407
RuO_x@UiO-66(Zr/Ti)-NH_2	27	419	338
RuO_x@UiO-66(Zr/Ti)-NH_2 Used	15	736	353
UiO-66(Zr)-NO_2	10	896	369
UiO-66(Zr/Ti)-NO_2	11	557	358
RuO_x@UiO-66(Zr/Ti)-NO_2	99	448	309
RuO_x@UiO-66(Zr/Ti)-NO_2 Used	7	464	317

Table S3. Different vibrational modes of adsorbed CO over RuO_x@UiO-66(Zr/Ti)-NO_2 based on literature. [10, 11]			
Species	Frequencies (cm^{-1})	Support	Reference
$Ru^{3+}(CO)_2$	2124, 2054, 2069	ZrO_2	1, 2, 3
$Ru^{2+}(CO)$	2079, 2032	SiO_2	4, 5
$Ru^{2+}(CO)_2$	2070, 2004, 1970	ZrO_2	2, 3, 6
$Ru^{\sigma+}(CO)$	1995, 1987, 1955	TiO_2	5
$Ru^0(CO)$	2023, 2080	SiO_2	5

Table S4. Vibrational modes of different adsorbed species over RuO_x@UiO-66(Zr/Ti)-NO_2 during the CO_2 methanation reaction based on literature.[12-15]				
Band position with $^{12}CO_2$ (cm^{-1})	Band position with $^{13}CO_2$ (cm^{-1})	Δ(cm^{-1})	Possible Assignment	Ref.
1175	1171	4	H-CO bend. (formyl)	1
1172	1169	3	H-CO bend. (formyl)	1
1160	1056	4	Stretching (CO) of linear methoxys on Zr^{4+}	2
1147	1144	3	rocking of methoxys	3
1130	1127	3	Dioxymethylene	4
1060	1057	3	Bridged methoxys on Zr^{4+}	2,3

References

(1) Rueda-Navarro, C. M.; Cabrero-Antonino, M.; Escamilla, P.; Díez-Cabanes, V.; Fan, D.; Atienzar, P.; Ferrer B.; Vayá, I.; Maurin, G.; Baldoví, H. G.; et al. Solar-assisted photocatalytic water splitting using defective UiO-66 solids from modulated synthesis. *Nano Res.* **2024**, 17, 4134–4150

(2) Shearer, G. C.; Chavan, S.; Bordiga, S.; Svelle, S.; Olsbye, U.; Lillerud, K. P. Defect engineering: tuning the porosity and composition of the metal−organic framework UiO-66 via modulated synthesis. *Chem. Mater.* **2016**, *28*, 3749−3761.

(3) Cavka, J. H.; Jakobsen, S.; Olsbye, U.; Guillou, N.; Lamberti, C.; Bordiga, S.; Lillerud, K. A new zirconium inorganic building brick forming metal organic frameworks with exceptional stability. *J. Am. Chem. Soc.* **2008**, *130*, 13850-13851.

(4) Santiago-Portillo, A.; Navalón, S.; lvaro, M.; García, H. Generating and optimizing the catalytic activity in UiO-66 for aerobic oxidation of alkenes by post-synthetic exchange Ti atoms combined with ligand substitution. *J. Catal.* **2018**, *365*, 450-463.

(5) Sun, D.; Liu, W.; Qiu, M.; Zhang, Y.; Li, Z. Introduction of a mediator for enhancing photocatalytic performance via post-synthetic metal exchange in metal–organic frameworks (MOFs). *Chem. Commun.* **2015**, *51*, 2056-2059.

(6) Cabrero-Antonino, M.; Ferrer, B.; Baldoví, H. G.; Navalón, S. Toward solar-driven photocatalytic CO2 methanation under continuous flow operation using benchmark MIL-125(Ti)-NH2 supported ruthenium nanoparticles. *Chem. Eng. J.* **2022**, *445*, 136426.

(7) Cabrero-Antonino; M.Melillo, A.; Montero-Lanzuela, E.; Álvaro, M.; Ferrer, B.; Vayá, I.; Baldoví, H. G.; Navalón, S. Solar-driven gas phase photocatalytic CO2 methanation by multimetallic UiO-66 solids decorated with RuOx nanoparticles. *Chem. Eng. J.* **2023**, *468*, 143553.

(8) Wang, S.; Cabrero-Antonino, M.; Navalón, S.; Chen-chen Cao, C.-c.; Tissot, A.; Dovgaliuk, I.; Marrot, J.; Martineau-Corcos, C.; Yu, L.; Wang, H.; et al. A robust titanium isophthalate metal-organic framework for visible-light photocatalytic CO2 methanation. *Chem* **2020**, *6*, 3409-3427.

(9) Cabrero-Antonino, M.; Remiro-Buenamañana, S.; Souto, M.; García-Valdivia, A. A.; Choquesillo-Lazarte, D.; Navalón, S.; Rodríguez-Diéguez, A.; Mínguez-Espallargas, G.;

García, H. Design of cost-efficient and photocatalytically active Zn-based MOFs decorated with Cu_2O nanoparticles for CO_2 methanation. *Chem. Commun.* **2019**, *55*, 10932-10935.

(10) Hadjiivanov, K.; Lavalley, J.-C.; Lamotte, J.; Maugé, F.; Saint-Just, J.; Che, M. FTIR Study of CO Interaction with Ru/TiO_2Catalysts. *J. Catal.* **1998**, *17*, 415-425.

(11) Guglielminotti, E.; Boccuzzi, F.; Manzoli, M.; Pinna, F.; Scarpa, M. Ru/ZrO_2 Catalysts: I. O_2, CO, and NO Adsorption and Reactivity. *J. Catal.* **2000**, *192*, 149-157.

(12) Morgan Jr, G. A.; Sorescu, D. C.; Zubkov, T.; Yates Jr, J. T. The formation and stability of adsorbed formyl as a Possible Intermediate in Fischer− Tropsch chemistry on ruthenium. *J. Phys. Chem. B* **2004**, *108*, 3614-3624.

(13) Finocchio, E.; Daturi, M.; Binet, C.; Lavalley, J.; Blanchard, G. Thermal evolution of the adsorbed methoxy species on $Ce_xZr_{1-x}O_2$ solid solution samples: a FT-IR study. *Catal. Today* **1999**, *52*, 53-63.

(14) Schild, C.; Wokaun, A.; Baiker, A. On the mechanism of CO and CO_2 hydrogenation reactions on zirconia-supported catalysts: a diffuse reflectance FTIR study: Part I. Identification of surface species and methanation reactions on palladium/zirconia catalysts. *J. Mol. Catal.* **1990**, *63*, 223-242.

(15) Panayotov, D. A.; Burrows, S. P.; Morris, J. R. Photooxidation mechanism of methanol on rutile TiO_2 nanoparticles. *J. Phys. Chem. C* **2012**, *116*, 6623-6635.
